# The Tetraspanin-Associated Uroplakins Family (UPK2/3) Is Evolutionarily Related to PTPRQ, a Phosphotyrosine Phosphatase Receptor

**DOI:** 10.1371/journal.pone.0170196

**Published:** 2017-01-18

**Authors:** Javier U. Chicote, Rob DeSalle, José Segarra, Tung-Tien Sun, Antonio García-España

**Affiliations:** 1 Research Unit, Hospital Universitari de Tarragona Joan XXIII, Institut d’Investigació Sanitària Pere Virgili, Universitat Rovira i Virgili, Tarragona, Spain; 2 Sackler Institute for Comparative Genomics, American Museum of Natural History, New York, New York, United States of America; 3 Department of Urology, Hospital Universitari de Tarragona Joan XXIII, Institut d’Investigació Sanitària Pere Virgili, Universitat Rovira i Virgili, Tarragona, Spain; 4 Departments of Cell Biology, Dermatology, Biochemistry and Molecular Pharmacology, and Urology, New York University School of Medicine, New York, New York, United States of America; Universita degli Studi di Roma Tor Vergata, ITALY

## Abstract

Uroplakins are a widespread group of vertebrate integral membrane proteins that belong to two different families: UPK1a and UPK1b belong to the large tetraspanin (TSPAN) gene family, and UPK3a, UPK3b, UPK3c, UPK3d, UPK2a and UPK2b form a family of their own, the UPK2/3 tetraspanin-associated family. In a previous study, we reported that uroplakins first appeared in vertebrates, and that uroplakin tetraspanins (UPK1a and UPK1b) should have originated by duplication of an ancestor tetraspanin gene. However, the evolutionary origin of the UPK2/3 family remains unclear. In this study, we provide evidence that the UPK2/3 family originated by gene duplication and domain loss from a protoPTPRQ-like basal deuterostome gene. PTPRQs are members of the subtype R3 tyrosine phosphatase receptor (R3 PTPR) family, which are characterized by having a unique modular composition of extracellular fibronectin (FN3) repeats, a transmembrane helix, and a single intra-cytoplasmic phosphotyrosine phophatase (PTP) domain. Our assumption of a deuterostome protoPTPRQ-like gene as an ancestor of the UPK2/3 family by gene duplication and loss of its PTP and fibronectin (FN3) domains, excluding the one closest to the transmembrane helix, is based on the following: (i) phylogenetic analyses, (ii) the existence of an identical intron/exon gene pattern between UPK2/3 and the corresponding genetic region in R3 PTPRs, (iii) the conservation of cysteine patterns and protein motifs between UPK2/3 and PTPRQ proteins and, (iv) the existence in tunicates, the closest organisms to vertebrates, of two sequences related to PTPRQ; one with the full subtype R3 modular characteristic and another without the PTP domain but with a short cytoplasmic tail with some sequence similarity to that of UPK3a. This finding will facilitate further studies on the structure and function of these important proteins with implications in human diseases.

## Introduction

Uroplakins (UPKs) are integral membrane proteins belonging to two families: UPK1a (UPIa) and UPK1b (UPIb) are members of the four-transmembrane domain tetraspanin (TSPAN or TM4SF) family [1,2]; and the single-spanning transmembrane uroplakins UPK2a (also known as UPII, encoded by the UPK2 gene), UPK2b (non-mammalian vertebrates), UPK3a (UPIIIa, UPK3A), UPK3b (UPIIIb, UPK3B), UPK3c (UPK3BL; present in mammals, reptiles and birds) and UPK3d (bony fish) form the tetraspanin-associated uroplakin family, UPK2/3 [3–7].

UPKs play important roles in the expansion and stabilization of the urothelial apical surface and contribute to the urothelium permeability barrier function in mammals [8]. Their protein structure, function and involvement in urinary tract development and malformations are the subject of several reports and reviews [5,7,9–16].

In a previous report, we showed that both the tetraspanin uroplakins and the UPK2/3 families first appeared in the common ancestor of vertebrates [7]. While the origin of UPK1a and UPK1b can be explained by gene duplication from an ancestor invertebrate tetraspanin gene [2,5,17], the origin of the UPK2/3 family is not yet established as it does not appear to be related to any known protein family [7].

PTPRQ is a member of the large family of phospho-tyrosine phosphatases that, along with the protein tyrosine kinases, regulates the level of phospho-tyrosine modifications in cells [18,19]. Specifically, PTPRQ belongs to subtype R3 phospho-tyrosine phosphatase receptors (R3 PTPRs), which like UPK2/3 uroplakins are single-spanning membrane proteins. However they are much larger than UPK2/3 proteins, which consist of an extracellular moiety of less than 200 amino acids and a short cytoplasmic tail, absent in UPK2a and UPK2b [3,7,20]. R3 PTPRs have a single cytoplasmic catalytic phophotyrosine phosphatase (PTP) domain and an extracellular region composed exclusively by fibronectin type III (FN3) domains (9 to 19 in humans). In vertebrates, R3 PTPRs comprise five members: PTPRB (VE-PTP), PTPRH (SAP-1), PTPRJ (DEP-1), PTPRO (GLEPP1) and PTPRQ (PTPS31) [21–23].

In this study, we show that UPK2/3 proteins are related to and are probably derived from an ancestor R3 PTPRQ-like gene in invertebrates. This finding is based on the results of phylogenetic analyses with UPK2/3 and R3 PTPR DNA and protein sequences of vertebrates and invertebrates, and the identity of the intron/exon gene structures and the conservation of several cysteines pairs and protein motifs.

## Materials and Methods

### Data mining and sequence analysis

Data mining was performed as described [2,21,24]. Sequences with the R3 PTPR characteristics of only FN3 domains and a single PTP catalytic domain were retrieved from major taxonomic groups of organisms by searching in online genomic databases and scientific reports [23–25]. The SMART server [26] and the NCBI CDD database [27] were used to determine the domain composition of the proteins. Since evolutionarily related genes have more conserved intron positions than merely similar sequences [28,29], intron-exon borders were determined as detailed in Garcia-España *et al*. [30] using the “align two sequences” option of the NCBI BLAST program [31]. Splice consensus signals were then manually annotated. All R3 PTPRs sequences used in this study plus organisms’ scientific names, accession numbers and structural characteristics are shown in S1 File. The UPK2/3 sequences used in this study have been reported elsewhere [5,7].

### Protein sequence alignments

All protein sequence alignments were performed using the MAFFT server [32] or the ClustalW and the Multalin programs at the NPS@: Network Protein Sequence Analysis [33]. The NCBI TBLASTN program was used in protein profiling [31].

### Phylogenetic analyses

We compared the sequences from representative vertebrates (*Homo sapiens*, *Mus musculus*, *Gallus gallus*, *Xenopus tropicalis*, and *Danio rerio*), invertebrate deuterostomes (*Strongylocentrotus purpuratus*, *Saccoglossus kowalevskii*, *Ciona intestinallis* and *Ciona savignyi*), protostomes (*Capitella teleta*, *Drosophila melanogaster* and *Caenorhabditis elegans*), and a porifera (*Amphimedon queenslandica*). In addition, we compared the corresponding regions of representative R2A subtype leukocyte common antigen-related (PTPRF) phosphatases and PTPRF from *Monosiga brevicollis* as outgroup. Alignments were generated using TranslatorX (with the MAFFT option and default settings) [34]. In this way, both DNA and protein sequence matrices were produced.

Examination of the DNA sequence matrices suggested extreme saturation of the data. Hence, we focused on using the amino acid sequences to infer the phylogenetic relationships of the various gene family members. We also coded the molecular motifs and intron positions for these genes as characters. This resulted in six binary characters (all scored as present = G, absent = T) C1–C2 (cysteine 1, 2), C3–C4 (cysteine 3, 4), intron 1, intron 2, intron 3 and intron 4. The present/absent characters were coded as G and T respectively to allow Bayesian and maximum parsimony analysis of combined amino acid sequence and molecular motif data. We generated Bayesian trees using MrBayes [35] for the amino acid sequences alone, the molecular motif (MM) data alone and for the two partitions combined (using a partitioned analysis with parameters for the amino acid sequences as below and the parsimony setting in MrBayes for the MM data). For Bayesian analysis of amino acid sequences, the mixed model using the “prset aamodelpr = mixed” command with 500000 generations (at which point the 2 analyses of MrBayes converged; split standard deviation = 0.0105) was used. Parsimony bootstraps were conducted using 1000 replicates of random taxon addition and TBR branch swapping in PAUP* [36]. The tree we present is from a Bayesian analysis of the combined matrix. Other trees generated using protein sequences alone, MM characters alone and bootstrap maximum parsimony analyses for combined and MM alone and protein alone are shown in S1 Fig. All matrices used in the analyses are given in S2 File.

### Tyrosine phophorylation and furin cleavage sites prediction

Putative phosporylation residues and furin cleavage sites were predicted with NetPhos 2.0 and ProP 1.0 Servers [37,38].

## Results and Discussion

### UPK2/3 uroplakins conserve intron/exon and cysteine patterns

**Fig 1:**
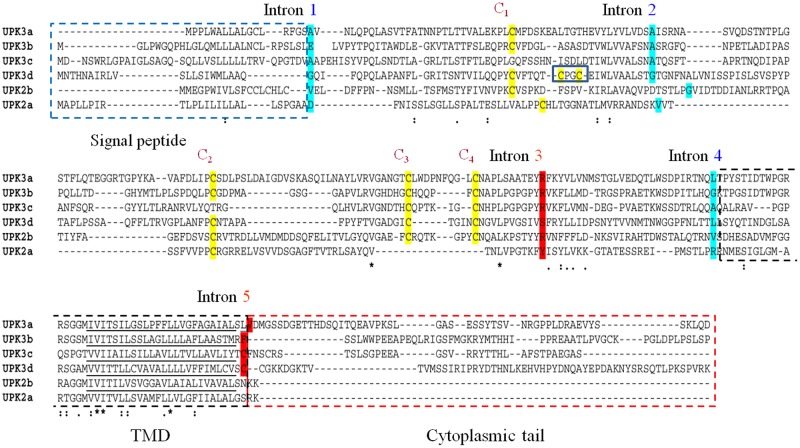
Conservation of UPK2/3 uroplakins intron/exon pattern and cysteine pairs in vertebrates. The alignment of the full-length sequences of human UPK3a, UPK3b, UPK3c, UPK2a, lizard UPK2b and zebrafish UPK3d was generated by the MAFFT server with default parameters, except for the position of intron one, which was manually curated and aligned. Introns 1 to 5 are indicated above the sequences. The amino acids split by the introns are shaded in blue and red according to phase 1 and 2, respectively. Conserved cysteines are shaded in yellow, and numbered 1 to 4 from the N-terminus. Note that UPK3d has two additional cysteines (blue box) and that C3–C4 in UPK2a and C1–C2 in UPK3c are not conserved in all the organisms (for an alignment of a full vertebrate set of UPK2/3 proteins see S2 Fig). The signal peptide, transmembrane domain and cytoplasmic tail are enclosed in blue, black and red dashed boxes, respectively. The amino acids in the transmembrane helices are underlined. Note that although the sequences of UPK2/3 members are only weakly similar, their intron/exon conjunctions and several key cysteine residues are highly conserved, strengthening their shared evolutionary origin. Amino acid identity: identical (*); strongly similar (:); weakly similar (.).

Our earlier phylogenetic analyses showed that UPK2/3 uroplakins probably originated from an unknown ancestor gene by a major duplication event in the first stages of vertebrate evolution [5,7]. UPK2/3 overall amino acid sequence identity is low. Their orthology is also shown by the following: (i) the location of their intron/exon junctions and the phases of the introns are conserved (phases 1, 1, 2, 1, 2 in the marked introns 1 to 5 in Fig 1 and S2 Fig; phase 1 and 2 denotes that the intron is located between the 1st and 2nd or 2nd and 3rd nucleotides of the codon, respectively), with the exception of intron 5, which is lost in UPK2 genes since they lack the exon coding for the cytoplasmic tail (Fig 1 and S2 Fig); and (ii) they share a set of highly conserved extracellular cysteine residues (C1–C4 in Fig 1 and S2 Fig). Thus UPK3a, UPK3b, UPK3c, UPK3d and UPK2b of reptiles and birds contain the four cysteines, but only UPK3c of birds and reptiles contains C1 and C2, UPK2a contains only C1 and C2 and UPK3d contains an additional pair (Fig 1 and S2 Fig). The above observations indicate that UPK2/3 uroplakins are variations on a common structural theme with UPK2a and UPK2b genes losing the cytoplasmic tail, UPK3d gaining a pair of cyteines, and UPK2a and UPK3c losing cysteine pairs C3 and C4 and C1 and C2, respectively.

### UPK2/3s and R3 PTPRs share similar extracellular juxtamembrane and transmembrane regions

**Fig 2:**
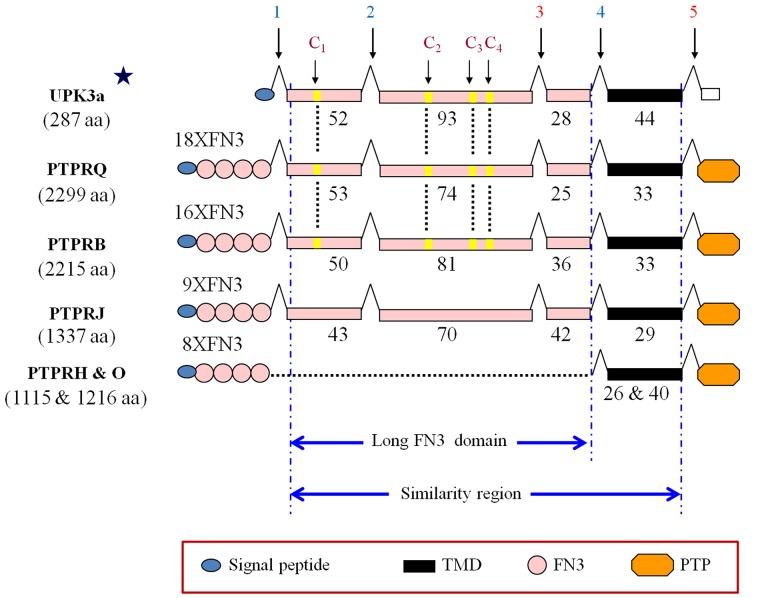
Shared region between UPK2/3 uroplakins and human PTPRQ, PTPRB and PTPRJ R3 PTPR members. Symbols include: long FN3-like juxtamembrane domain exons, pink boxes; transmembrane domain, black box; numbers below the boxes indicate the number of amino acid residues; introns (vertical arrows numbered 1 to 5); blue and red color denote phase 1 (between the 1st and 2nd positions) and phase 2 (2nd and 3rd); star above the human UPK3a sequence indicates that although only human UPK3a sequence is shown, all UPK2/3 human proteins share the same intron/exon pattern with the exception of exon 5, which is absent in UPK2a and UPK2b (see Fig 1 and S2 Fig); C1–C4 indicate the four cysteines of UPK3a conserved in PTPRQ and PTPRB, but not in PTPRJ. Note the absence of the long FN3-like juxtamembrane domain in PTPRH and PTPRO (dotted line). See box for additional symbols for signal peptides, catalytic PTP domains and FN3 domains (number indicated). The size of the proteins is indicated in parenthesis below the protein names.

To study the evolutionary origin of the UPK2/3 genes, we performed searches in protein and genomic databases with several UPK2/3 sequences as bait. We first found that the UPK3a human sequence (except for the N-terminal signal peptide and the C-terminal cytoplasmic tail) was similar to the juxtamembrane long FN3-like and transmembrane domains of the human R3 phosphatase receptor PTPRQ between amino acids 1744 and 1928 (S3 Fig) [39]. This similarity was also evident in PTPRB and to a lesser extent in PTPRJ, but not in the other human R3 PTPR members PTPRO and PTPRH, which lacked the longer FN3-like domain located between introns 1 and 4 (Fig 2).

Moreover, these regions are encoded by 4 exons of similar lengths flanked by introns in phases 1, 1, 2, 1, 2 in PTPRQ, PTPRB and PTPRJ as in UPK2/3 genes (Fig 2) In addition, PTPRQ and PTPRB have also the four characteristic UPK2/3 C1–C4 cysteine residues (Fig 2).

### UPK2/3 and R3 PTPR long juxtamembrane FN3 domain is conserved in vertebrate and invertebrate organisms

By searching genomic databases and scientific reports (see Materials and Methods section), we retrieved full sequences of R3 PTPR genes, which can be differentiated from other PTPR receptor subtypes (R1, R2, R4–R8) by their unique domain composition consisting of only FN3 domains in the extracellular moiety and a single catalytic (PTP) domain in the cytoplasmic region [21]. We obtained R3 PTPR sequences from representative organisms covering a broad evolutionary range, from sponges to mammals (S1 File).

Interestingly, in tunicates, the closest living organisms to vertebrates [40], we retrieved one R3-related sequence without a PTP domain, (CionaNocat.1 and CionaNocat.2 from *Ciona intestinalis* and *Ciona savignyi*, respectively) in addition to another sequence with the full R3 specific modular characteristics (Ciona.2 and Ciona from *Ciona intestinalis* and *Ciona savignyi*, respectively).

All these invertebrate R3 PTPR-related sequences contained similar regions to the UPK2/3 genes with the exception of only Ciona1 and Oikopleura1 sequences (S1 File).

**Fig 3:**
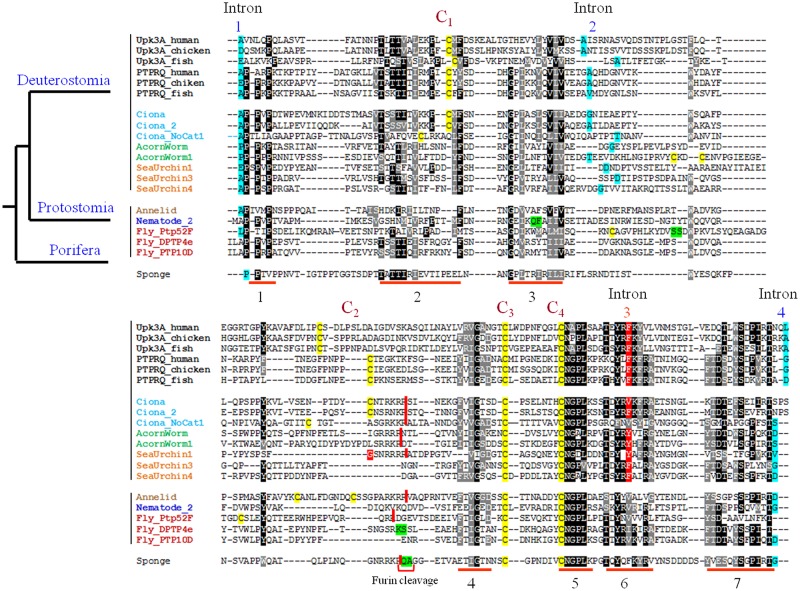
Characteristics of the long FN3-like juxtamembrane region in vertebrate and invertebrate R3 PTPR sequences. The long FN3-like domain of UPK3a, UPK3b, PTPRQ and PTPRB from representative vertebrates and R3 PTPR-related sequences from representative invertebrates were aligned using the MAFFT server with default parameters and printed out with BoxShade (identical and similar residues are represented by different grey shadings). The positions of the introns 1 to 4 are marked and the amino acids split between two exons (phase 0, 1 and 2) are shaded in green, blue and red, respectively; cysteine residues 1 to 4 are shaded in yellow; putative furin cleavage sites are marked with vertical red bars between the cleaved amino acids and high conserved short signature sequences are underlined in red numbered 1 to 7. The sequences cover vertebrates (black); tunicates (*Ciona*, blue); hemichordates (acorn worm, green); echinoderms (sea urchin, orange); lophotrochozoa (annelid, light brown); edquisozoan (nematode, blue and fruit fly, magenta); and porifera (sponge, purple). Organisms’ scientific names are provided in S1 File. PTPRB, UPK3b sequences were removed from the alignment and are depicted in S4 Fig along with UPK3a and PTPRQ sequences for comparison. UPK2 sequences were not included in the analysis since they lack the C3–C4 sequence region.

The alignment of these long FN3-like regions of vertebrate and invertebrate R3 sequences revealed several highly conserved features between R3 PTPRs and UPK2/3 uroplakins: (i) the flanking introns 1 and 4 were already present in sponges (porifera), while introns 2 and 3 are a deuterostome acquisition (Fig 3 and S4 Fig); (ii) cysteines C3 and C4 are present in all organisms from sponges to mammals and cysteines C1 and C2 only from tunicates (Fig 3); (iii) seven short fragments (marked 1 to 7 in Fig 3) are conserved in all sequences; of these, motif 5 (C3X(n)C4NGPL signature where X represents any amino acid) is particularly highly conserved and was found only in UPK2/3 and R3 PTPR proteins. These results indicate that the long FN3-like domain close to the TMD, as defined by several highly conserved sequence and genomic features, is present in the R3 PTPR sequence of sponges and in almost all invertebrates including the sequences without PTP domain in tunicates.

**Fig 4:**
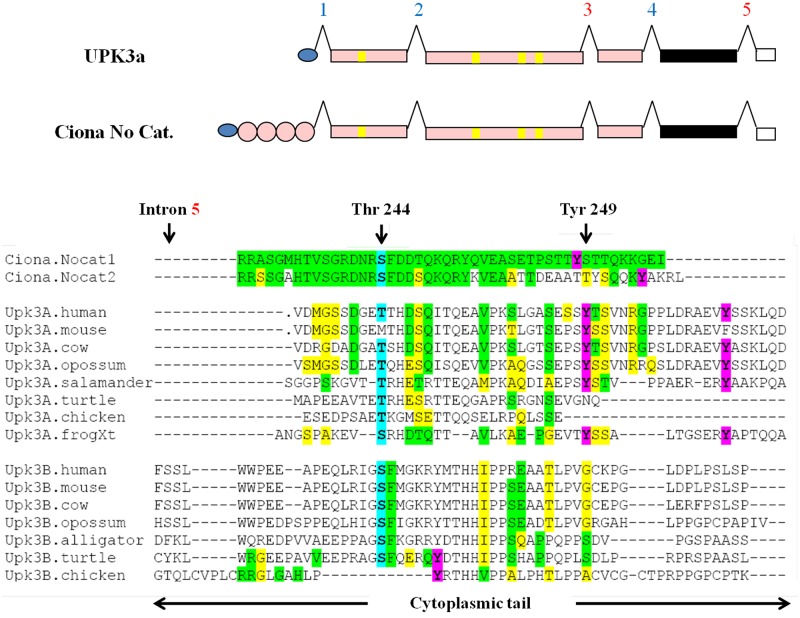
Similarity between the cytoplasmic tails of UPK3a and the *Ciona* R3 PTPR sequences without a catalytic domain (CionaNocat.1 and CionaNocat.2). Upper panel: cartoon of UPK3a and either one of the *Ciona* sequences with no catalytic domain (symbols are the same as in the legend to Fig 2). Lower panel: alignment of UPK3a, UPK3b, CionaNocat.1 and CionaNocat.2 cytoplasmic tail sequences. Code for shaded regions: residues identical and similar to the CionaNocat.1 sequence are highlighted in green and yellow, respectively. Predicted phosphoritable tyrosine residues are colour in magenta. The frog tyrosine (Tyr249), which is transiently phosphorylated upon gamete fertilization, and the human threonine (Thr244), which is phosphorylated upon adhesion of uropathogenic *E*. *coli* to the urothelium, are marked with vertical arrows and Thr/Ser244 are coloured in blue. Intron 5 position just after the TMD domain is marked by a vertical arrow.

Moreover, the cytoplasmic tails of these sequences without PTP domains (CionaNocat.1 and CionaNocat.2) have retained some sequence similarity with those of UPK3a genes (Fig 4), although vertebrates and tunicates diverged over 700 MYA [41]. This similarity includes two putatively phosphorylatable residues highly conserved in UPK3 genes (Fig 4): the Tyr249 in *Xenopus* that is highly conserved in vertebrate UPK3a genes and is transiently phosphorylated during *Xenopus* oocyte fertilization [16], and the human Thr244 that is highly conserved as a Ser/Thr in vertebrate UPK3a and 3b genes and is phosphorylated by a serine/threonine kinase following uropathogenic *Escherichia coli* adhesion to the urothelium [42] (Fig 4).

### Phylogenetic analyses indicate that UPK3 genes are evolutionarily related to PTPRQ

**Fig 5:**
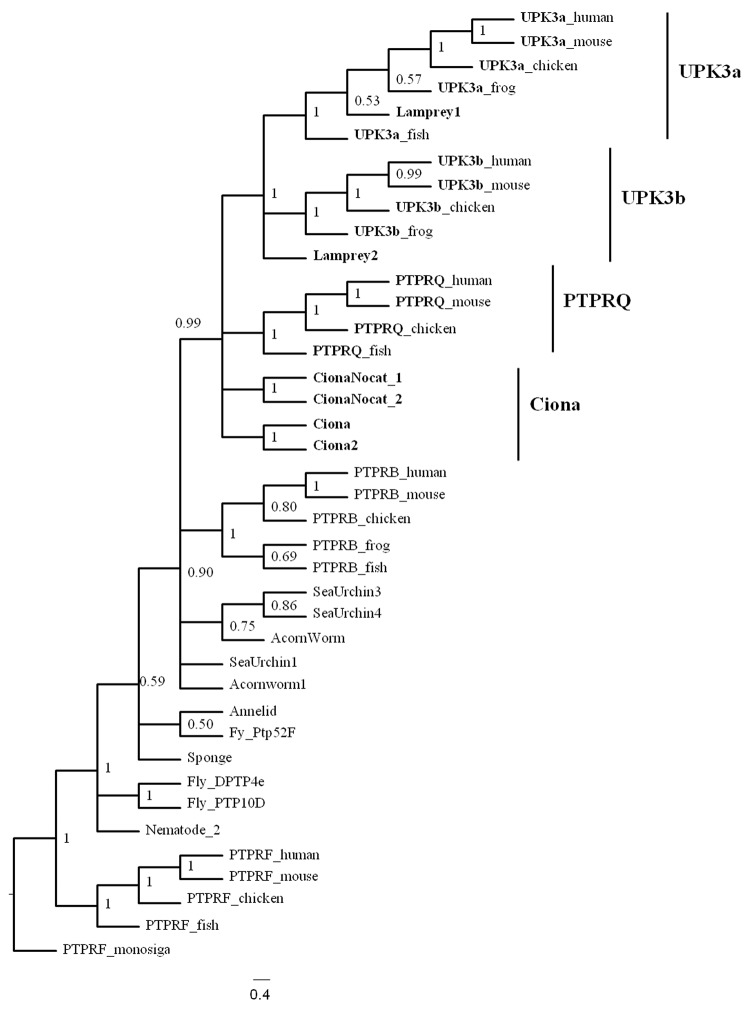
Evolutionary relationship between UPK3 and PTPRQ genes. The evolution of the similarity region in a broad spectrum of species spanning sponge to mammals was analyzed using a combined protein sequence and molecular morphology matrix (see text for details). Bayesian phylogenetic analysis was used to generate a tree with posterior probabilities given at the nodes in the tree (see Materials and Methods section for details of Bayesian analysis). *Monosiga* R5 PTPRF was used as outgroup since R5 PTPRF is the closest to R3 PTPR.

In view of the above observations, we examined the evolutionary relationship between UPK2/3 and R3 PTPR genes by phylogenetic analyses using the long FN3-like and TMD domain, shared regions between these proteins. Using the protein matrix, the molecular morphology matrix and a matrix that combines the two and doing bootstrap analysis on all parsimony based analyses resulted in eight separate trees that we present in S4 Fig. Of these eight trees, six support the inclusion of UPKs as embedded in the PTPR family and one is unresolved (protein only with MP and bootstrap). One analysis (protein only with MP) pushed the UPKs outside of the PTPRs. Of all the analyses in the table this one would be most prone to the simplification of the parsimony model. Since the Bayesian analysis of the combined matrix (Fig 5) incorporated a model of sequence change we suggest that for the most part it is the most appropriate type of analysis for the data set we used. Fig 5 and S4 Fig summarize the phylogenetic analyses of these regions of vertebrate PTPRB, PTPRQ, UPK3a, and UPK3b and invertebrate R3 PTPR genes from representative groups of organisms. We present only the analysis performed with protein sequences combined with the molecular motifs (MM) characters since DNA sequences were saturated and could not be resolved in the analysis (see Materials and Methods section). The amino acid sequences combined with MM characters (presence/absence of introns and cysteines) placed the UPK3 and PTPRQ genes as sister groups in Bayesian analysis (Fig 5 and S1 Fig). Other analyses were ambiguous as to which PTPR was sister to the UPK3 genes except for the protein plus MM maximum parsimony analysis. The important result with respect to UPKs is that all trees ruled out PTPRF (from subtype R2A PTPR) as a potential sister group (Fig 5 and S1 Fig) except for the analysis of protein with MP bootsrap.

In addition to showing that UPK3a and UPK3b are most likely sister groups of the PTPRQ genes, the phylogenetic analysis demonstrated that UPK3 genes are also closely related to the *Ciona* sequence with a PTP catalytic domain (ciona and ciona2) and the *Ciona* sequence without a PTP domain (CionaNocat.1 and 2). These sequences without the catalytic domain and with only five FN3 domains closely resemble the intermediate stage in the transition of a PTPRQ-like gene to a UPK2/3 gene in the common ancestor of tunicates and vertebrates.

Taken together, the data suggest that UPK2/3 uroplakins originated by gene duplication, a common mechanism for the generation of new genes [43,44], coupled with domain loss from a PTPRQ precursor gene in the common ancestor of vertebrates over 700 MYA when vertebrates radiated from tunicates [41].

### Structure and function of the juxtamembarene FN3-like domain

Phosphotyrosine phosphatase receptors (PTPR) play important roles in cell-to-cell interaction and signaling [18,19]. The diversity of these proteins is achieved principally through changes in the extracellular, non-catalytic region, of the receptors [19]. Although the number of the FN3 domains in R3 PTPR genes is variable (8 to 18) [42], the long FN3-like region close to the transmembrane helix in R3 PTPRs is highly conserved from sponges to mammals, suggesting that this domain performs essential functions. Indeed, the biological significance of this domain is demonstrated by the observation that in angiosarcomas, 36% of the inactivating mutations of the recessive cancer gene PTPRB are over-represented in the long FN3-like region, which represents only 7.5% of the protein sequence [25].

An interesting result from these analyses is that since cysteines that form disulfide bridges are almost always gained or lost as in pairs during protein evolution [45], the emergence of C1 and C2 in the R3 PTPR sequences of *Ciona* (urochordates) coupled to the specific loss of C3 and C4 in UPK2a and of C1 and C2 in UPK3c (Figs 1 and 3 and S2 Fig), suggest that C1 forms a disulfide bridge with C2 and C3 forms a disulfide bridge with C4, *in situ*. Additionally, an important feature of UPK2a is its prosequence that is cleaved after cysteine C2, at an Rx(R/K)R furin motif. It has been suggested that this prosequence blocks the oligomerization of the six UPK1a/2a-UPK1b/3a heterotetramers, thus preventing them from forming a 16-nm particle [3,11,46]. This UPK2a furin site is also conserved in some UPK2b proteins and in many invertebrate R3 PTPR proteins (ciona, ciona 2, cionanocat, acornworm, acornworm1, sea urchin1, annelid, PTP52 F from *Drosophila melanogaster* and the sponge protein from *Amphimedon queenslandica* (S5 Fig). These UPK2a cleaved fragments probably remain bound to the tetraspanin uroplakin UPK1a partner [12]. Proteolytic cleavage of proteins close to the transmembrane domain without the release of the cleaved fragment could introduce flexibility in the protein proximal region and has been observed in FN3 domains of other receptors PTPs including PTPRμ and PTPRκ that are processed in the trans-Golgi network by subtilin-like convertases [47].

Tetraspanins and many kinases involved in cell signaling are enriched in lipid rafts [48]. It is possible that the common ancestral protein of PTPR and UPK2/3 genes were clustered in lipid raft domains where they interacted with a UPK1a/1b tetraspanin precursor, followed by co-evolution between these two uroplakin families. This idea is supported by the reported association of an unknown tyrosine phosphatase with CD53 and CD63 in lymph node cells [49].

## Conclusions

The discovery that UPK2/3 proteins are most likely derived from an ancestral PTPRQ gene by gene duplication and domain loss and that UPK2/3 and R3 PTPR genes share a juxtamembrane long FN3 domain with conserved structural characteristics will facilitate further studies on the structure and function of these important proteins with implications in human disease.

## Supporting Information

S1 Fig Phylogenetic trees of the similarity region sequences from R3 PTPR and UPK2/3 proteins generated using parsimony analysis and Bayesian analysis. 1A) Trees generated from Protein + MM matrix using maximum parsimony. The tree on the left is the maximum parsimony tree and the tree on the right is a bootstrap tree using maximum parsimony. 1B) Trees generated from the MM matrix alone. The tree on the left is the maximum parsimony tree and the tree on the right is a bootstrap tree using maximum parsimony. 1C) Trees generated from the Protein sequences alone matrix. The tree on the left is the maximum parsimony tree and the tree on the right is a bootstrap tree using maximum parsimony. 1D) Trees generated using Bayesian analysis. The tree on the left is the protein only Bayesian analysis and the tree on the right is the MM + Protein Bayesian analysis.
(PDF)

S2 Fig Cysteine and intron positions in UPK2/3 proteins. Sequences of UPK2/3 proteins from mammals to lamprey were aligned with MAFFT using default parameters. The alignment does not include the UPK2/3 first exon sequence, which encodes the signal peptide, and the last exons, which encode the cytoplasmic tail in UPK3 genes but it is absent in UPK2s. The UPK2/3 cysteines (C1–C4) are highlighted in yellow and the two additional UPK3d cysteines in green. Exons are displayed in alternate colors. Amino acids in red indicate that their codons are split between adjacent exons. The intron positions 1–5 are indicated above the sequences in blue and red numbers for intron phases 1 and 2, respectively. The transmembrane helix (TMD) is underlined in blue. Note the specific loss of C1–C2 in marsupial and mammalian UPK3c genes and C3–C4 in UPK2a genes.
(PDF)

S3 Fig Human PTPRQ and UPK3A shared protein regions. Alignment of the similarity region of human UPK3a amino acids 17 to 235 (upper line) and PTPRQ amino acids 1744 to 1928 (lower line). Transmembrane helices and cysteine residues are underlined and highlighted in yellow, respectively, in the sequence. Amino acid identity: identical (*); strongly similar, (:); weakly similar, (.).
(TIF)

S4 Fig Conservation of the long FN3-like juxtamembrane region in UPK3b, UPK2a and PTPRB vertebrate sequences that were omitted in Fig 3 due to space constraints. (TIF)

S5 Fig Predicted furin cleavage sites in UPK2/3 and R3 PTPR long FN3-like domains. Cleavage sites are indicated by a vertical arrow. C2 cysteine residues are highlighted in yellow.
(TIFF)

S1 File List of all R3 PTPR sequences and their accession numbers used in this study. The similar region and transmembrane helix are highlighted in green and red, respectively. PTP catalytic domains are underlined. Exons are displayed in alternate colours. Amino acids in bold red colour indicate they are split between adjacent exons by a phase 1 or 2 intron. The C1–C4 cysteine residues and the CNGPL motif are highlighted in yellow.
(PDF)

S2 File All protein and DNA sequences matrices used in the phylogenetic analyses. (PDF)
