# Correction: The Tetraspanin-Associated Uroplakins Family (UPK2/3) Is Evolutionarily Related to PTPRQ, a Phosphotyrosine Phosphatase Receptor

**DOI:** 10.1371/journal.pone.0172416

**Published:** 2017-02-13

**Authors:** Javier U. Chicote, Rob DeSalle, José Segarra, Tung-Tien Sun, Antonio García-España

The following information is missing from the Funding section: This work was supported by Spanish MINECO and the European Regional Development Fund (ERDF) grant CONSOLIDER INGENIO 2010 CSD00065 (A.G.E).
