# Supplementary figures and images for: The Tetraspanin-Associated Uroplakins Family (UPK2/3) Is Evolutionarily Related to PTPRQ, a Phosphotyrosine Phosphatase Receptor
Source: PLoS One. 2017 Jan 18;12(1):e0170196. doi: 10.1371/journal.pone.0170196 (PMC5242461; doi:10.1371/journal.pone.0170196)

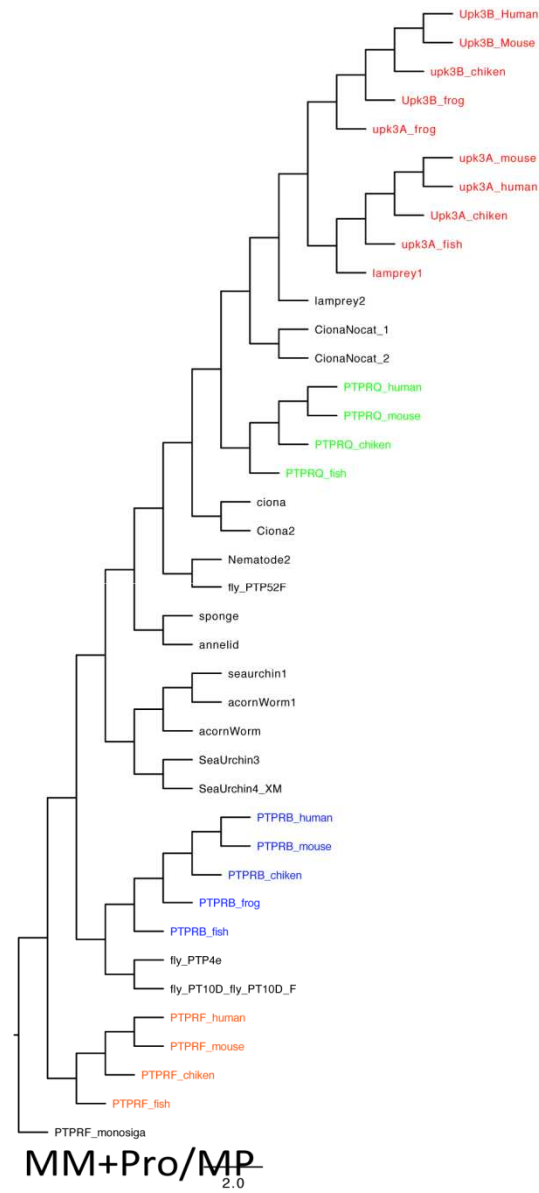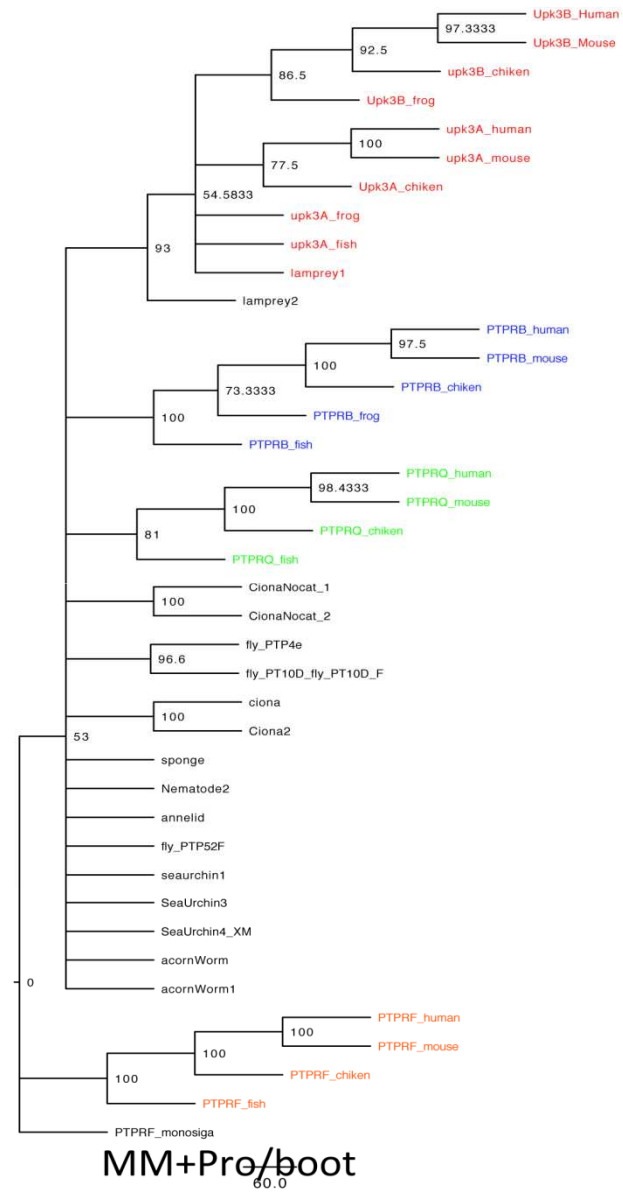

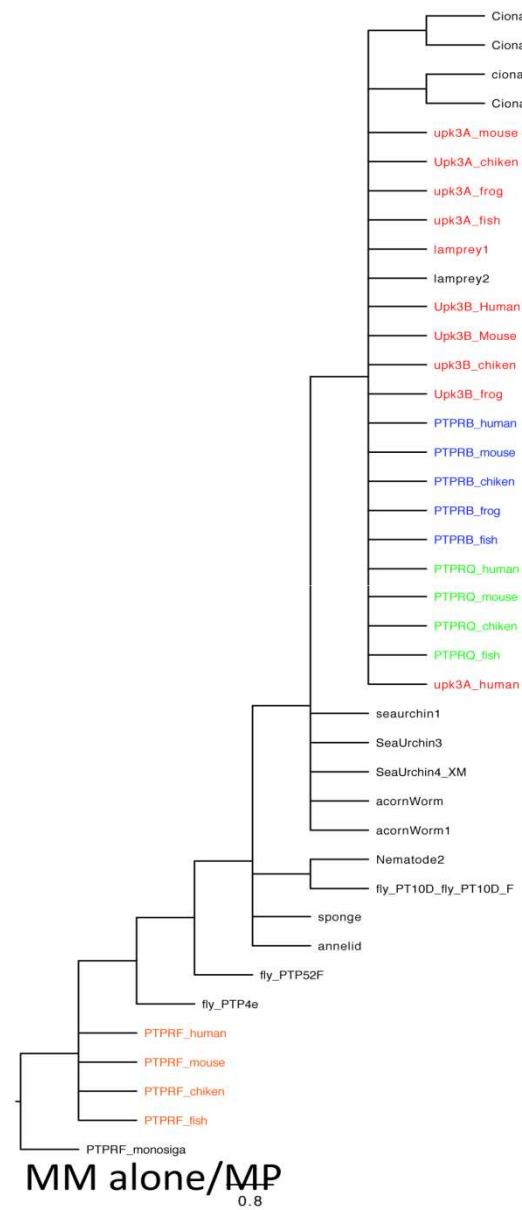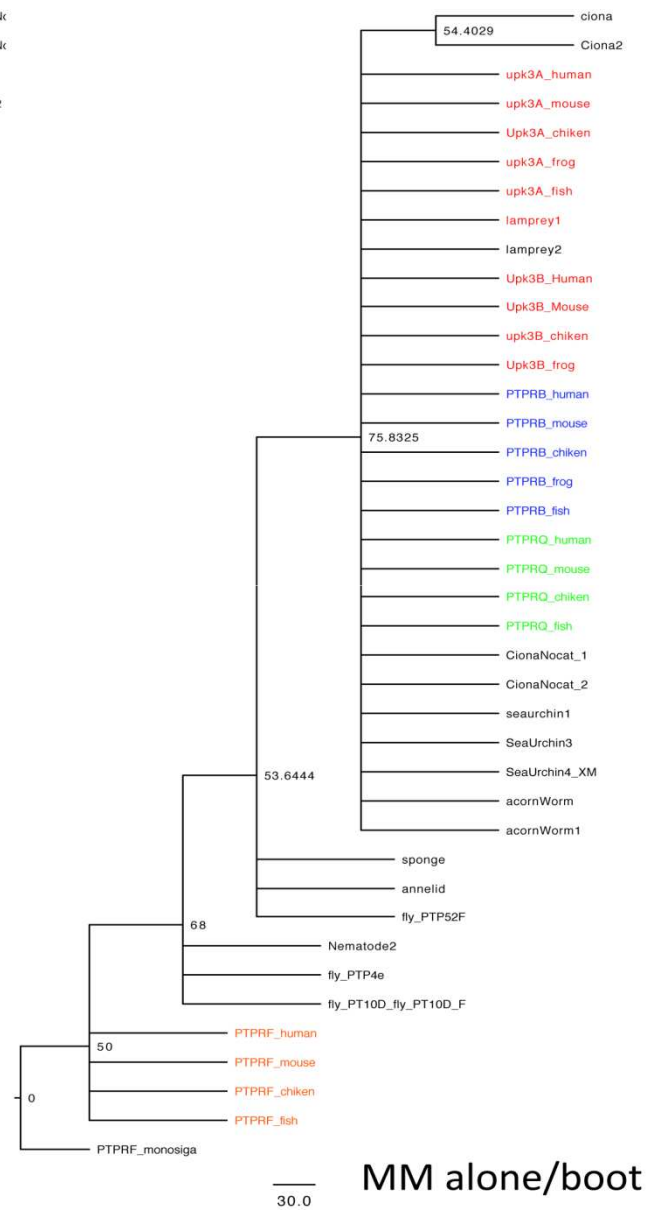

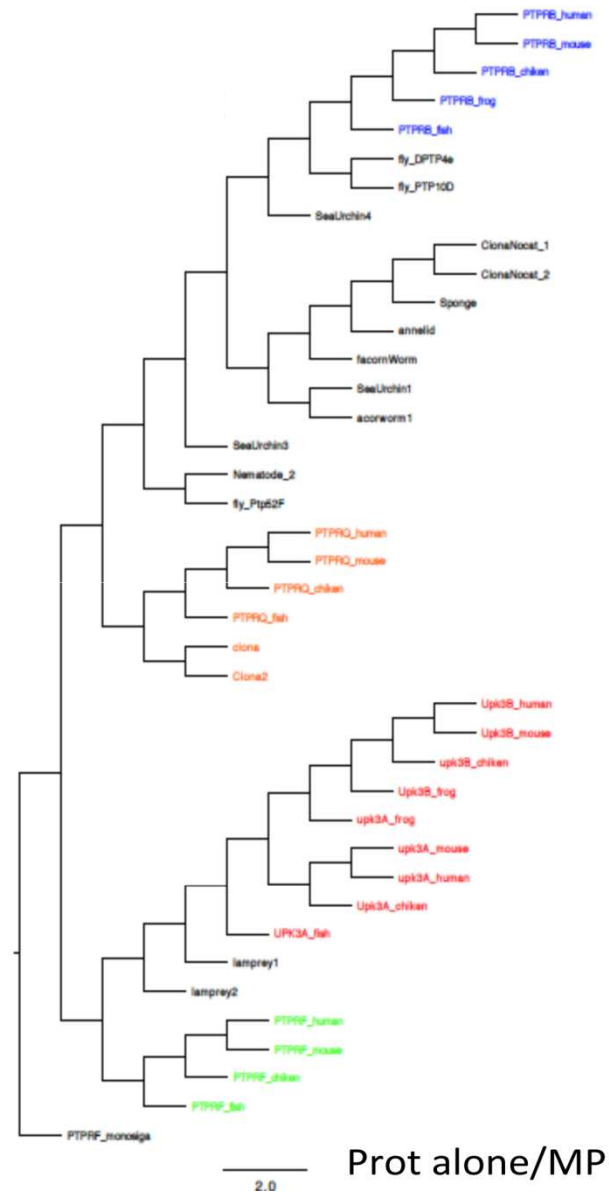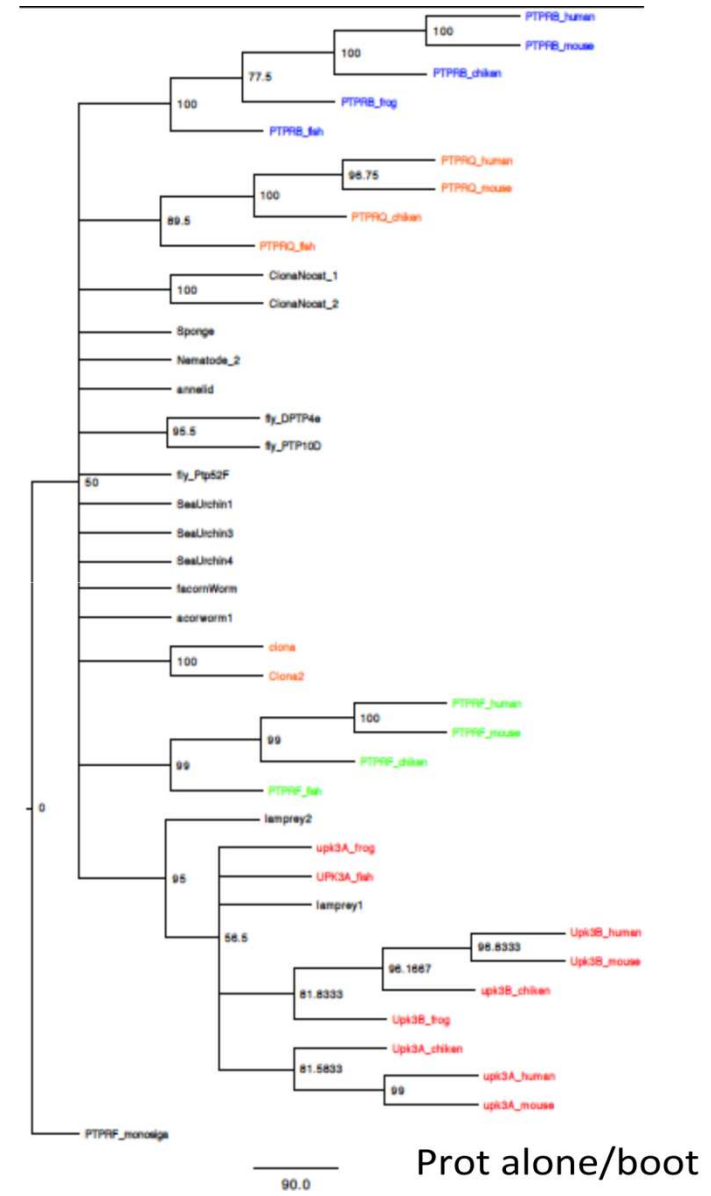

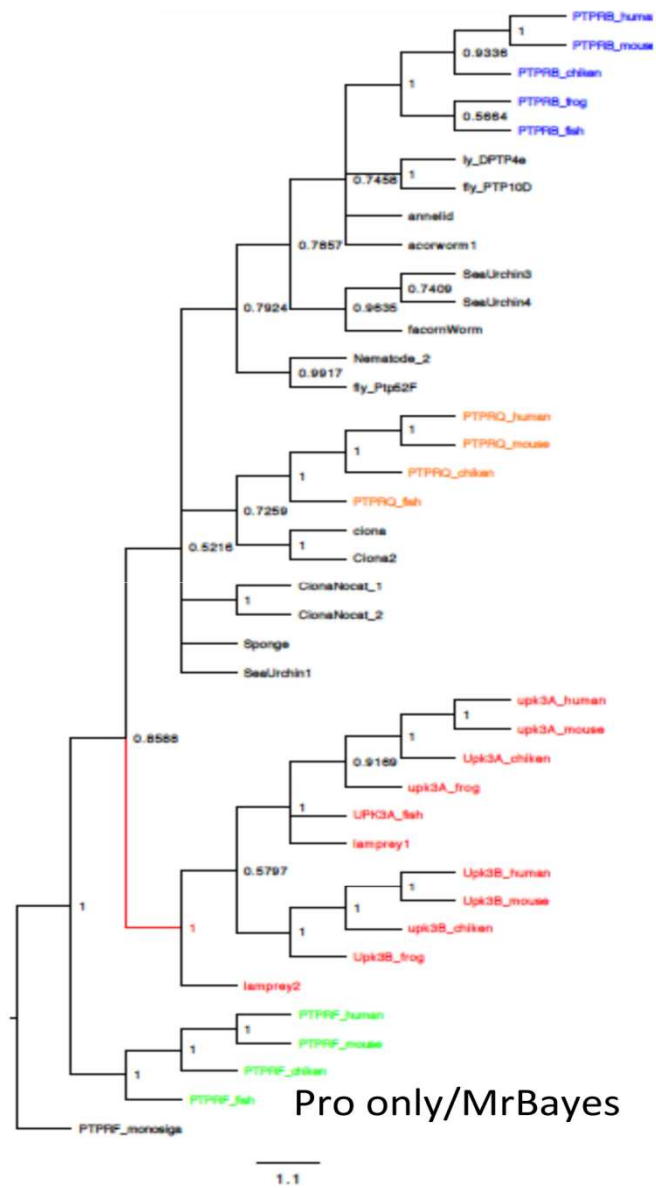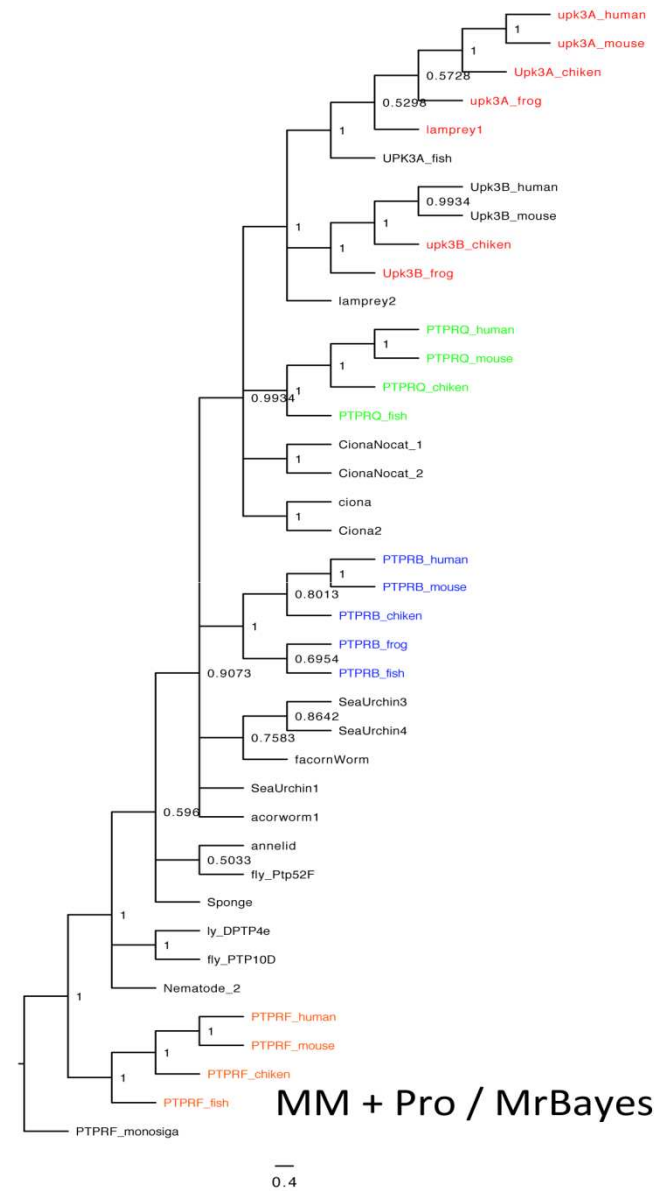

Supplement: S1 Fig — 1A) Trees generated from Protein + MM matrix using maximum parsimony. The tree on the left is the maximum parsimony tree and the tree on the right is a bootstrap tree using maximum parsimony. 1B) Trees generated from the MM matrix alone. The tree on the left is the maximum parsimony tree and the tree on the right is a bootstrap tree using maximum parsimony. 1C) Trees generated from the Protein sequences alone matrix. The tree on the left is the maximum parsimony tree and the tree on the right is a bootstrap tree using maximum parsimony. 1D) Trees generated using Bayesian analysis. The tree on the left is the protein only Bayesian analysis and the tree on the right is the MM + Protein Bayesian analysis. (PDF) [file pone.0170196.s001.pdf]

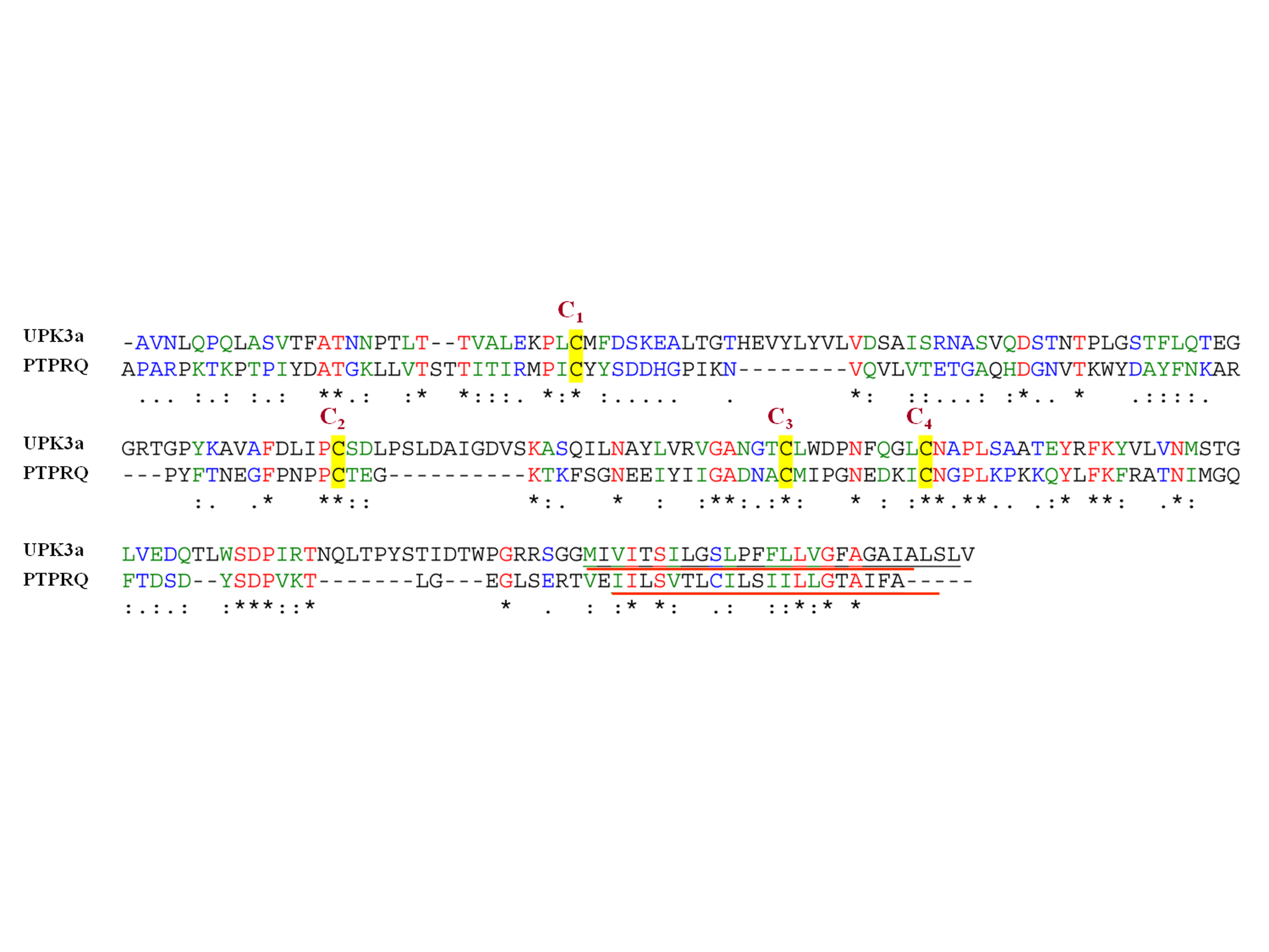

Supplement: S3 Fig — Alignment of the similarity region of human UPK3a amino acids 17 to 235 (upper line) and PTPRQ amino acids 1744 to 1928 (lower line). Transmembrane helices and cysteine residues are underlined and highlighted in yellow, respectively, in the sequence. Amino acid identity: identical (*); strongly similar, (:); weakly similar, (.). (TIF) [file pone.0170196.s003.tif]

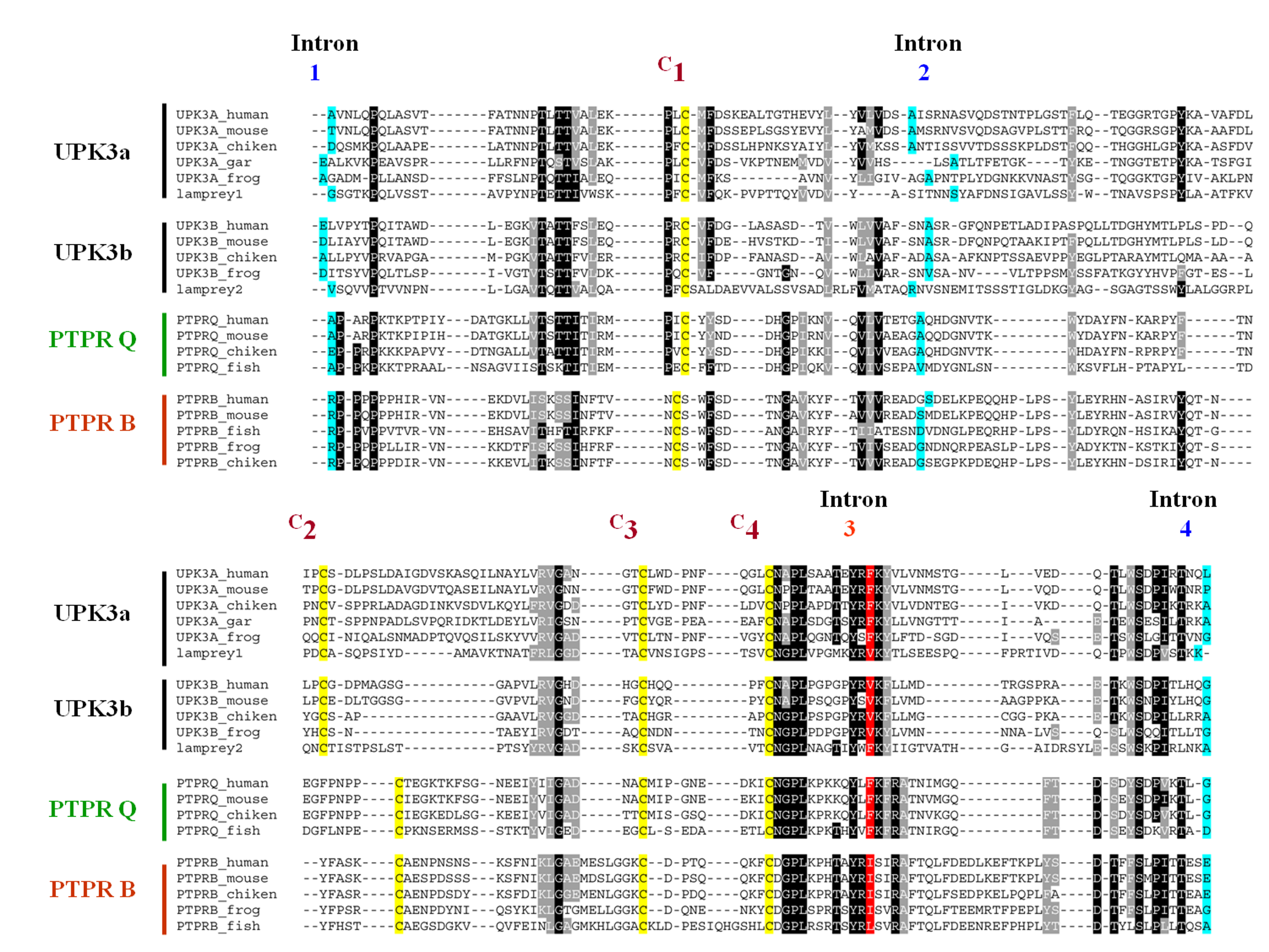

Supplement: S4 Fig — (TIF) [file pone.0170196.s004.tif]

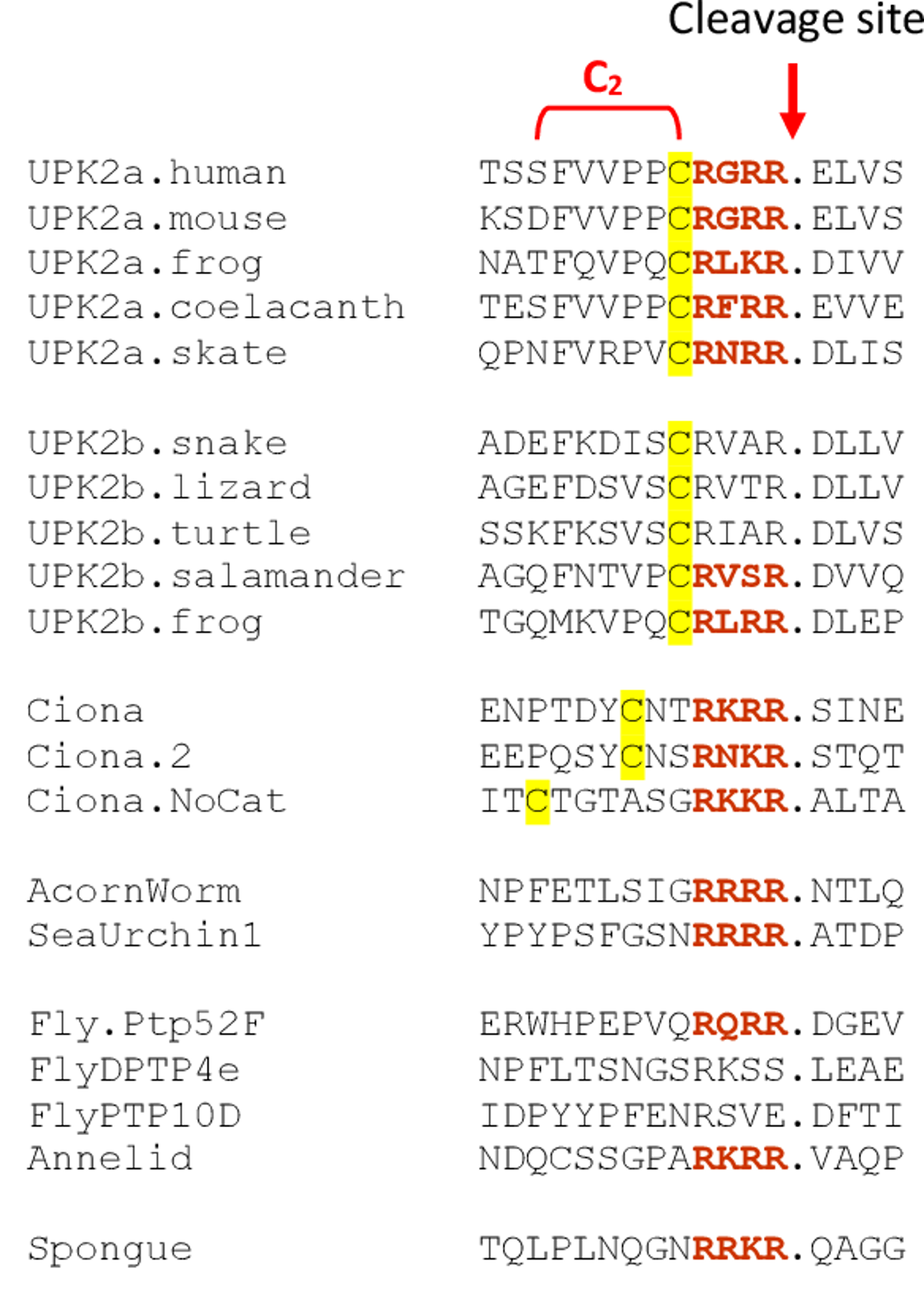

Supplement: S5 Fig — Cleavage sites are indicated by a vertical arrow. C2 cysteine residues are highlighted in yellow. (TIFF) [file pone.0170196.s005.tiff]
