# Supplementary material for: The Tetraspanin-Associated Uroplakins Family (UPK2/3) Is Evolutionarily Related to PTPRQ, a Phosphotyrosine Phosphatase Receptor
Source: PLoS One. 2017 Jan 18;12(1):e0170196. doi: 10.1371/journal.pone.0170196 (PMC5242461; doi:10.1371/journal.pone.0170196)
Supplement: S2 Fig — Sequences of UPK2/3 proteins from mammals to lamprey were aligned with MAFFT using default parameters. The alignment does not include the UPK2/3 first exon sequence, which encodes the signal peptide, and the last exons, which encode the cytoplasmic tail in UPK3 genes but it is absent in UPK2s. The UPK2/3 cysteines (C1–C4) are highlighted in yellow and the two additional UPK3d cysteines in green. Exons are displayed in alternate colors. Amino acids in red indicate that their codons are split between adjacent exons. The intron positions 1–5 are indicated above the sequences in blue and red numbers for intron phases 1 and 2, respectively. The transmembrane helix (TMD) is underlined in blue. Note the specific loss of C1–C2 in marsupial and mammalian UPK3c genes and C3–C4 in UPK2a genes. (PDF) [file pone.0170196.s002.pdf]

```
                      1                               C1
UPK3a.human      ----AVNLQP-QLA--SVTFATNNPTLTTVALEKPL--C----MFDSK---------EAL
UPK3a.mouse      ----TVNLQP-QLA--SVTFATNNPTLTTVALEKPL--C----MFDSS---------EPL
UPK3a.cow        ----GVNLQP-QLA--SVTFATNNPTLTTVALEKPL--C----MFDSS---------AAL
UPK3a.elephant   ----AVNLQP-QLA--SVTFTTNNPTLTTVALEKPL--C----MFESN---------TAL
UPK3a.dog        ----GVNLQP-QLA--SVTFATNNPTLTTVALEKPL--C----MFDSA---------ATL
UPK3a.opossum    ----AVDLEP-QMA--SITFATNNPTLTTITLEKPF--C----MFNAS---------NLG
UPK3a.platypus   -----AQIKP-QIA--GLAFATNNPTLTTIALEKPF--C----MFDGA---------GPA
UPK3a.chicken    ----DQSMKP-QLA--APELATNNPTLTTVALEKPF--C----MFDSS---------LHP
UPK3a.aligator   ----AQSLRP-QIA--SPQLATNNPTFTTIALEKPF--C----VFDGS---------LSP
UPK3a.turtle     ----ALNLRP-QIA--NPKLATSNPTLTTIALEKPF--C----MFDDS---------LSP
UPK3a.Xtropical  ----AGADMP-LLA--NSDFFSLNPTQTTIALEQPI--C----MFKSA---------V--
UPK3a.Xlaevis    ----ARSAVP-LLA--NSDFFSLNPTQTTITLERPF--C----MYKDA---------I--
UPK3a.salamande  ----TFAVTP-QVA--GPALVPNTPTLNTVALQKPV--C----LFDSQ---------TGG
UPK3a.coelacant  ----AQFPKP-EIA--NPQFVAGNPTLTTISLEKPF--C----VFDRT---------ISA
UPK3a.spottedga  ---EALKVKP-EAV--SPRLLRFNPTQSTVSLAKPL--C----VFDSV---------KPT
UPK3a.skate      ----PLSFKP-EVS--SIALTLGLRTSTTVTLAKPI--C----VFSSG---------D--
UPK3a.shark      ----ILQFKP-EMP--SVSTIDGLRTARTVTLSKPL--C----VLPTG---------H--
UPK3a.1.lamprey  ----GSGTKP-QLV--SSTAVPYNPTETTIVWSKPF--C----VFQKP---------VPT
UPK3a.2.lamprey  ----VSQVVP-TVV--NPNL-LGAVTQTTVALQAPF--C----SALDAEVVAL----SSV
UPK3b.human      ---ELVPYTP-QI---TAWDLEGKVTATTFSLEQPR--C----VFDGL---------ASA
UPK3b.cow        ---DLIPYTP-RI---TSWDLEGKVTATTFSLEQPR--C----VLDRH---------SSA
UPK3b.mouse      ---DLIAYVP-QI---TAWDLEGKITATTFSLEQPR--C----VFDEH---------VST
UPK3b.elephant   --------DPLHA---TDNGLGGKVTATTFSLEQPR--C----VLDGH---------ARA
UPK3b.dog        ---ELIPYTP-QI---TAWDLEGKVTATTFSLEQPR--C----VLDGP---------ASV
UPK3b.platypus   ----LMSYKP-QV---SAHPLEGKVTASTFTLDQPR--C----VFDGQ---------VVS
UPK3b.opossum    ---DQIPYTP-QI---SALALEGKVTAATFSLEQPR--C----IFSEL---------AAP
UPK3b.Xtropical  ---DITSYVP-QL---TLSPIVGTVTSTTFVLDKPQ--C----VFGNT------------
UPK3b.Xlaevis    ---DITTYVP-QL---TLMPIQGSVTSTTFTLDKPQ--C----IFGSR------------
UPK3b1.Xtropica  ----IPYYVP-QI---TTKPILGKLTSSSFVLEQPQ--C----IFQQY-----------K
UPK3b2.Xtropica  ---DVGSYVP-KI----TTSILGNLTFSTFVLEQPQ--C----IFSSNY----------P
UPK3b.chicken    ---ALLPYVP-RV---APGAMPGKVTATTFVLERPR--C----IFDPF---------ANA
UPK3b.shark      ----VKNYTP-EV---TNANLAGRITATTITFQQPL--C----QFDSE---------INA
UPK3b.aligator   ---ELVPYTP-QI---TGNSLEGKLTASTFSLDQPI--C----IFDQY---------VNA
UPK3d.coelacant  ----DVPYLP-EV---AFNNVAGSVTATTFTLQQPR--C----IFKDV---------FSS
UPK3d.1.spotted  --VTSVEYTP-EI---TAYNMAGRVTGTTMVMKQPR--C----YFDNQLLLPC----TPD
UPK3d.salmo      --IVSLDHIP-EI---LPYEASGRVTSTTIVLGQPL--C----YFNTLTQLKC----SQS
UPK3d.trout      --IVSLDHIP-EI---LPYEASGRVTSTTIVLGQPL--C----YFNTLTQLKC----SQS
UPK3d.zebrafish  ----GQIFQP-QL---APANFLGRITSNTVILQQPY--C----VFTQ---------TCP
UPK3b.skate      --ADEADFVP-EI---FKDNVIGGVSQTTFALQQPQ--C----VFDRFQ-------PACT
UPK3d1.salmo     ---IPVNNTP-EI---TPSSLAAKLTSNSVILTSPS--C----YFNGLGNLSC---NSTT
UPK3d1.trout     ----PVNNTP-AI---NPSSLAAKLTINSVILTSPS--C----YFNSLANLPC---NSTT
UPK3d.spottedga T-VPSVDYVP-EI---IPMKLAGRLTGTTFLLRQPL--C----YFSNQQGLNC----SLS
UPK3d2.spottedg -----VPYAP-EV---TPHNLLGRVTSTTLTLQQPV--C----FFNNLSGLPC----APD
UPK3c.human     AAPEHISYVP-QL---SNDTLAGRLTLSTFTLEQPL--G----QFSSHN--------ISD
UPK3c.mouse      ---ESINYAP-QL---LGATLEGRLTQSTFTLEQPL--G----QFKNVN--------LSD
UPK3c.dog        ---EHISYVP-QL---SNRSLAGTLTQSTFTLEQPR--G----QFSHRS--------ISD
UPK3c.cow        ---ERISYVP-QL---SSATLAGRLTQSTFTLEQPR--G----QFSHPS--------ISD
UPK3c.elephant   ---ELISYVP-RL---SNATLAGKLTQSTFTLQQPR--G----RFSHK---------ISD
UPK3c.opossum    ---EPINYTP-AI---TREPLEGSITSSTFTLDQPN--D----QFNGSG--------ISD
UPK3c.platypus   ----KIAYTP-VI---TKLPMEGKITLSTFTLDQPL--G----QFNSSA--------VDD
UPK3c.lyzard     -----VNYTP-RL---ASENLGGKVTASTFTLDQPR--C----VFNDV---------VNA
UPK3c.chicken    ---DKLSYKP-TL---VGGNVEGRMTGSTFVLEQPR--C----VFDSY-----------S
UPK3c.turtle     ----SIDYTP-VI---TARELEGKITSSTFVLEQPR--C----VFNDS---------VSN
UPK2a.human      ------DFNI-----SSLSGLLSPALTESLLVALPP--C----HLTGG------------
UPK2a.mouse      ------DFNI-----SSLSGLLSPALTESLLIALPP--C----HLTGG------------
UPK2a.dog        ------DFNI-----SSVSGLLSPALTESLLVALPP--C----HLTGG------------
UPK2a.cow        -----ADFNI-----SSLSGLLSPVMTESLLVALPP--C----HLTGG------------
UPK2a.elephant   ------DFHI-----SSLSGVLSPALTESLLVALAP--C----RLTGG------------
UPK2a.opossum    ------EFNI-----SSLSGLLSPALAESLLVALPP--C----HLTGG------------
UPK2a.platypus   -------FNI-----SSLSGLLTPTLAESLLVALPP--C----HLTGG------------
UPK2a.salamande  --QTAQNFST------SLADLPINPLQTLAIVAFPP--CWVGVSQSNA------------
UPK2a.Xlaevis    -----------QNT--SLADGVLTPLSTSVIIAFPG--C----KDSGK------------
UPK2a.Xtropical  -----------QNV--SQATGVLTPLATSAIFAFPD--C----TYSGQ------------
UPK2a.coelacant  ------NFNT-SLS--DNAALISNVYSSFVILSLPP--C----TYAGK------------
UPK2a.skate      ------GFTI-SLANDNMGEVVASRRSMSAIITMDPNSC----NLAGE------------
UPK2a.zebrafish  ------DIPI-SLLNPNTDGVLASTFPNSFLLQMPD--C----SIYGNQ-----------
UPK2a.salmo      ------EFQV-SLLK-ESDGVVTGRFADSLLLSLPP--C----ALATQ------------
UPK2a.lamprey    ------DYQV-KLL--NTSVVRAAQTPQSVAFAVPS--CEI-AMIAGT------------
UPK2b.snake      -----MDFFR------NNSMLLTTFTGTTFIVNIPK--C----VSSSK---------FSP
UPK2b.turtle     ----TLDFFP------NASLILAARMSTSFIVNIPK--C----ISSSQ---------FTP
UPK2b.lyzard     ----ELDFFP------NNSMLLTSFMSTYFIVNVPK--C----VSPKD---------FSP
UPK2b.aligator   ----TLNFFP------NMSNILAARLSSSFIVNIPK--C----ISSSH---------YTP
UPK2b.salamande  ---PAFSFFG------NQDNIIGNRMGYSFITNIPS--C----ISGAG---------YTP
UPK2b.Xlaevis    ---SNFQFFT------NTDDVLGAVLSQSFMVNVPS--C----INAVG---------YVP
UPK2b.shark      ------DYDI------ALFNGEVSRLATLVWLSPAY--C----LFEKWVEQRGGELTVRS
```

2

```
UPK3a.human      TG-THEVYLYVLVD-SAISRNASVQDSTNTPLGS----TFLQTE------GGRTGPY---
UPK3a.mouse      SG-SYEVYLYAMVD-SAMSRNVSVQDSAGVPLST----TFRQTQ------GGRSGPY---
UPK3a.cow        HG-TYEVYLYVLVD-SASFRNASVQDSTKTPLSS----TFQQTQ------GGRTGPY---
UPK3a.elephant   VG-TYEVYLYVLAE-SASLRNASIQDHTSAPLSS----TFQQTE------GGRTGPY---
UPK3a.dog        NG-TYEIYLYVLVN-LASSRNASVQDGARAPLSS----TVQQTE------GGRTGPY---
UPK3a.opossum    NV-SYEVNLYVMEN-SGSVRYAVIKNNRSFPINS----TFQETA------GGQRAPY---
UPK3a.platypus   QL-PSEIYLYVMMD-SADAH--PVLDNGSRPLRT----TFQQAA------GGRRGPY---
UPK3a.chicken    NK-SYAIYLYVMKS-SANTISSVVTDSSSKPLDS----TFQQTH------GGHLGPY---
UPK3a.aligator   GK-SYEVYLYAMMD-SMSMISSAVTDNSSKPLDS----TFQEVN------GGQLGPY---
UPK3a.turtle     GS-SYEVYLYAMAD-SESTVSSAVTDNSSKPLNT----TFQDTN------GGQLGPY---
UPK3a.Xtropical -----NVYLIGIVA-GAPNT--PLYDGNKKVNAS----TYSGTQ------GGKTGPY---
UPK3a.Xlaevis   -----NVYLFAIVK-GATNI--QVADAAKKVIAS----NYTGTQ------GGLLGPY---
UPK3a.salamande DPANYQVELFAMAA-SAPSS-------TPLASGN----TFRNTS------GGTTGPY---
UPK3a.coelacant SSGDFTVAVFAVKS-TVAVA------ENINDFTQ----TYQSSR------EGTTAPY---
UPK3a.spottedga NE--MMVDVYVVHS-LSATL--------TFETGK----TYKETN------GGTETPY---
UPK3a.skate     -----VVEVFGVQT-TADSI--------PIEIGNRTILTYQQTD------GGARGPY---
UPK3a.shark     -----MIELLVVQG-NVTPI--------ATNLDG----TYQATK------GGATGPY---
UPK3a.1.lamprey TQ--YVVDVYASIT-NNSYA----FDNSIGAVLS----SY-WTN------AVSPSPY---
UPK3a.2.lamprey SA---DLRLFVMATAQRNVSNEMITSSSTIGLDK----GY--------------------
UPK3b.human     SD---TVWLVVAFS-NAS---------------R----GFQNPE------TLADIP----
UPK3b.cow       AD---TVWLVVAFS-NAS---------------R----VFQNPQ------TLAEIP----
UPK3b.mouse     KD---TIWLVVAFS-NAS---------------R----DFQNPQ------TAAKIP----
UPK3b.elephant  TD---TVWLVVAFS-NAS---------------K----DFQKPK------TQAEIP----
UPK3b.dog       AS---TVWLVVTFS-NAS---------------K----DFHNPQ------TLAEIP----
UPK3b.platypus  TD---TIWLVVAFS-NAS---------------R----DFQNPT------TAASIP----
UPK3b.opossum   AD---AVWLVVAFS-NAT---------------E----DFQNPK------TAAEIP----
UPK3b.Xtropical GN---QVWLLVARS-NVS---------------A----NV-----------VLTPPS---
UPK3b.Xlaevis   TN---QVWLLVARS-NVS---------------V----SITN--------AMLKPPS---
UPK3b1.Xtropica TS---LVWLVVALN-RVI---------------P----QLSY--------TQLSNPA---
UPK3b2.Xtropica TQ---DVWLVVALD-TVE--------------------PFLTD-------TNLSTPV---
UPK3b.chicken   SD---AVWLAVAFA-DAS---------------A----AFKNPT------SSAEVP----
UPK3b.shark     TP-GLIFWLVVANSQRGQ---------------N----TFDA--------ITFVQPSL--
UPK3b.aligator  TD---DIWLVVAFA-NAT---------------S----SLKNPT------SRTDIP----
UPK3d.coelacant CPL-CQLWVAVATE-KGA---------------N----NLITTVG-----RPVTFA----
UPK3d.1.spotted KC---EIWLVAAVGSAGI---------------Q----NFDADKGKST--ILSESPYP--
UPK3d.salmo     TC---QVWAAIASG-PGI---------------N----NFDIDKLVAV-QIVSASPYP--
UPK3d.trout     TC---QVWAAIASG-PGI---------------N----NFDIDKLVAV-QIVSASPYP--
UPK3d.zebrafish GC---EIWLVAALS-TGT---------------G----NFNALVNISSPISLSVSPYP--
UPK3b.skate     LC---EIWLVVDNP-ANV---------------S----TFDTN-------MNTLTPSA--
UPK3d1.salmo    TC---EIWLVSAKD-TGV---------------S----NYDADNKMPY--IDTRSPYP--
UPK3d1.trout    TC---ELWLVSAID-TGV---------------S----NYDADKNRPF--IDTLSPYP--
UPK3d.spottedga TC---EIWLAVARE-AGV---------------N----NFDTDKVQPSFDIVSASPYP--
UPK3d2.spottedg NC---EIWLVIA---RGV---------------P----KFEAIKGNTS--VLMSSPYP--
UPK3c.human     LD---TIWLVVALS-NAT---------------Q----SFTAPR------TNQDIP----
UPK3c.mouse     PD---PIWLVVAHS-NAA---------------Q----NFTAPR------KVEDRH----
UPK3c.dog       SD---AIWLVVAHS-NAT---------------Q----NFSAPQ------RVEDIP----
UPK3c.cow       SD---AIWLVVAHS-NAT---------------Q----KFTAPQ------KVEDTP----
UPK3c.elephant  FD---AIWLVVAHS-NAT---------------Q----SFIAPQ------RVKDSP----
UPK3c.opossum   LD---DIWLVVAFS-NAS---------------Q----SFEPPQ------SAQDIP----
UPK3c.platypus  LD---DIWLVVAYS-NAT---------------D----NFSNPV------KPDEEY----
UPK3c.lyzard    TD---GIWLLVARS-DAA---------------R----NFTRPG------SPSELP----
UPK3c.chicken   TA---NIWLVVATR-AGM---------------N----AFNDS-------AQPGMPEW--
UPK3c.turtle    TD---EIWLVVALS-NGTS-------------IS----TFTNPT------SLQSLP----
UPK2a.human     -----NATLMVRRANDSK------------------------------------------
UPK2a.mouse     -----NATLMVRRANDSK------------------------------------------
UPK2a.dog       -----NATLMVRRANDSK------------------------------------------
UPK2a.cow       -----NATLTVRRANDSK------------------------------------------
UPK2a.elephant  -----NATLMVRRANDSK------------------------------------------
UPK2a.opossum   -----KASLTVRRVNESA------------------------------------------
UPK2a.platypus  -----NATLKVQGVNGSS------------------------------------------
UPK2a.salamande -----NLILVSKNSSGSS-----------------------------------------I
UPK2a.Xlaevis   -----TVNLIVANGTTT-------------------------------------------
UPK2a.Xtropical -----TVSLVITNSTSTATI----------------------------------------
UPK2a.coelacant -----NASVTYSKNSTSEDS----------------------------------------
UPK2a.skate     -----TVIVTVNNTSNGQVI----------------------------------------
UPK2a.zebrafish -----SVLLLYTEAPTNL------------------------------------------
UPK2a.salmo     -----SVTLEYNNTDTNESK----------------------------------------
UPK2a.lamprey   -----MLNIFVTPNTTGVEV----------------------------------------
UPK2b.snake     A----TVRIAIAQLPDASSLP-----------------GITDTDQIE---NLRRTPQALV
UPK2b.turtle    T----TIRPAVAIVGDKVAMP-----------------AVTDTNQIQ---SL--------
UPK2b.lyzard    V----KIRLAVAQVPDTSTLP-----------------GVIDTDDIA---NLRRTPQATI
UPK2b.aligator  T----TIRPAIAVLGETVGLP-----------------AVTDTNQIR---SLRNFTDAPI
UPK2b.salamande A----SILLAVS---TSAAVP-----------------GVSNTDAIK---SLLNDSRAQV
UPK2b.Xlaevis   S----TLKVAVANRNPTC---------------------MVDTDSIK---SLKTDPTAPV
UPK2b.shark     SA---SVQVELLREDNATFV----------------------------------------
```

C_2

```
UPK3a.human      -KAVAFDLI--------------PCSDLPSLD---AIG----DVSKASQILNAYLVRVGA
UPK3a.mouse      -KAAAFDLT--------------PCGDLPSLD---AVG----DVTQASEILNAYLVRVGN
UPK3a.cow        -KAAAFDLT--------------PCSDSPSLD---AVR----DVSRASEILNAYLIRVGT
UPK3a.elephant   -KAAAFDLQ--------------PCSDLPSLD---AVE----DVSRASEILSTYLVRVGA
UPK3a.dog        -KAVAFGLI--------------PCSDLPSLD---AVG----DVARASEILNAYLVRVGA
UPK3a.opossum    -KAASFILP--------------QCGDLPNLD---EAG----DVSKVAEILSAYLVRVGD
UPK3a.platypus   -WAATFSVP--------------RCEDLPQLW---DAG----DPARAPQILDAYLFRVGG
UPK3a.chicken    -KAASFDVP--------------NCVSPPRLA---DAG----DINKVSDVLKQYLFRVGD
UPK3a.aligator   -KAAVLNVP--------------DCASPPKLA---DIR----NVKKASDVLKQYLFRVGD
UPK3a.turtle     -RAALFNVP--------------NCASPPMLA---DVV----NVKKVSDVLKQYLFRVGD
UPK3a.Xtropical  -IVAKLPNQ--------------QCINIQALS---NMA----DPTQVQSILSKYVVRVGA
UPK3a.Xlaevis    -QVAKLDNP--------------KCENIQASN---IMA----DP-------NKYIVRVGG
UPK3a.salamande  -VAGKFGVP--------------NCTLVGSLT---PSQ----D-----DFLRQYIFRVGD
UPK3a.coelacant  -KAASFAVP--------------NCDSPLVLS---NPV----NVNVIRSLLNQYLIRIGD
UPK3a.spottedga  -KATSFGIP--------------NCTSPPNPA---DLS----VPQRIDKTLDEYLVRIGS
UPK3a.skate      -RAARFMTP--------------ICTSLP-FV---PSR----DPAVIRVQIEQYLFRVGD
UPK3a.shark      -RAARFANP--------------ECTSSS-FT---PST----DPTKIQTLIDRYFIRVGS
UPK3a.1.lamprey  -LAATFKVP--------------DCASQPSIY---DAM----------AVKTNATFRLGG
UPK3a.2.lamprey  -AGSGAGTS-SWYLALGGRP-LQNCTISTPSL-----------------STPTSYYRVGA
UPK3b.human      -ASPQLLTD-GHYMTLPLSPDQLPCGDPMA------------------GSGGAPVLRVGH
UPK3b.cow        -ASPRLLTD-GHYMTLPLTMDQLPCEDPAD------------------GSGRAPVLRVGN
UPK3b.mouse      -TFPQLLTD-GHYMTLPLSLDQLPCEDLTG------------------GSGGVPVLRVGN
UPK3b.elephant   -TFAQLLTD-GHCMTLPPSPVQLPCTDSVG------------------GSGSALLLRVGN
UPK3b.dog        -AFPRLLTD-GYYMTLPLSLDQLPCEDPEG------------------GGRSIPLLRVGN
UPK3b.platypus   -AYPRLLTD-YYYMTLKVSPDLYPCADA---------------------GGLSVLRVGT
UPK3b.opossum    -SYTELSSS-FYYMTLKLSPDLYPCEEE----------------------DIAVLRVGS
UPK3b.Xtropical  -MYSSFATK-GYYHVPFGTESLYHCSNTA-----------------------EYIRVGD
UPK3b.Xlaevis    -MYSSFPTQ-GYYHVPLGTEASYPCSNTA-----------------------DYIRVGD
UPK3b1.Xtropica  -NISSFETN-GFYHTLPVFGGDYPCADTS--------------------GQLSAMIYVGS
UPK3b2.Xtropica  -TYSSFTTN-KFYHTLRVRGADYPCFNES--------------------AMSLALLQVGA
UPK3b.chicken    -PYEGLPTA-RAYMTLQMAAAAYGCSAP----------------------GAAVLRVGG
UPK3b.shark      MTHQTASTA-DYYLTLRTLAERYLCPPTSS------------------SNGEMYFVRVGQ
UPK3b.aligator   -PYQRLSTA-LHYXTLRTTIXFFPCPRST---------------------NTSVLRVGS
UPK3d.coelacant  -GYQKLSQR-GYYFTMRSSKDLYKCVEN---------------------DDKVRVLRLGA
UPK3d.1.spotted  TAFTGSPPK-NYYLTKVGRQQVFPCQQSH----------------------GIVYFRVGD
UPK3d.salmo      IAFSSQTNR-MYFVTKLGRPKDFPCGQLP----------------------GIKYFRVGA
UPK3d.trout      IAFSSQTNR-MYFVTKLGRPKDFPCGQLP----------------------GIKYFRVGA
UPK3d.zebrafish  TAFLP-SSA-QFFLTRVGPLANFPCNTAP----------------------AFPYFTVGA
UPK3b.skate      ATYKDFLLN-GFYLTVKTARTDYACPQT---------------------MGQVYTLRVGD
UPK3d1.salmo     TAFSSNTSK-KYFLTKLGLQKAYPCPIVA----------------------GTGYFRVGS
UPK3d1.trout     TAFSSNTSK-KYFLTKLGFQKDYPCPIVA----------------------GTDYFRVGS
UPK3d.spottedga  EAFQ---NK-NYYVTRLGVQNNFLCAELP----------------------GIRYFRVGA
UPK3d2.spottedg  DAFRNNSSP-NYFLTKLGVQNAFPCAEST----------------------GFRFFRVGD
UPK3c.human      -APANFSQR-GYYLTLRANRVLY---QT---------------------RGQLHVLRVGN
UPK3c.mouse      -APANFDRN-GYYLTLRANRVHYKGGQP---------------------DSQLRVLRVGN
UPK3c.dog        -VPEDFTRR-GYYLTLMANRLLYPGNQP---------------------GNQLRVLRVGN
UPK3c.cow        -VPADFPQR-GYYLTLRASRALYPGGPP---------------------SNQLRVLRVGN
UPK3c.elephant   -APADLPLK-GYYLTLRASRALYPGDQA---------------------GNQLQVLRVGN
UPK3c.opossum    -YAATFLDK-KYYLTIRASRDLYSSKRG---------------------SQGISVLRVGN
UPK3c.platypus   -NLSELSKK-QYYMTMRATRDLYPGGNN---------------------SSFLHVLRVGQ
UPK3c.lyzard     --FQDLEKN-GLYLTLTNTAPASYPCPEPGA------------------AGGPLTVLRVGN
UPK3c.chicken    -SFQRFPTNTSAYLTLGAMQYHYGCPKP---------------------DRELTVLRVGS
UPK3c.turtle     -AFQKFPGS-PHYMTMGTSSLNYPCEKS---------------------SGQITVLRVGN
UPK2a.human      VVTSSFVVP--------------PCRGRRELV---SVV----DSGAGFTVTRLSAYQV--
UPK2a.mouse      VVKSDFVVP--------------PCRGRRELV---SVV----DSGSGYTVTRLSAYQV--
UPK2a.dog        VVKSSFVVP--------------SCRGRRELV---SVV----DSGAGFTVTRLSAYQV--
UPK2a.cow        VVRSSFVVP--------------PCRGRRELV---SVV----DSGSGFTVTRLSAYQV--
UPK2a.elephant   VVKSSFMVP--------------PCRGHRELV---SVV----DSGAGFTVTRLSAYQV--
UPK2a.opossum    GMTHNFTVP--------------PCRARRDLV---SVV----YNSGSFSITRLSAYQV--
UPK2a.platypus   VLQQRFVVP--------------PCRGRRELV---SVV----DSSAGFATTRLDAYQI--
UPK2a.salamande  TNNQTVPVP--------------PCRLRRDAV---FSS----DSSSGGTVITNIGFRV--
UPK2a.Xlaevis    VQNISLQVP--------------QCRLKRDVV---VIN----NSQSGNVQTVNVGYQI--
UPK2a.Xtropical  IQNATFQVP--------------QCRLKRDIV---VIN----NGQSGNVQTVNVGYQI--
UPK2a.coelacant  -KTESFVVP--------------PCRFRREVV---EVA----RQMEGFTVTDLLGFRV--
UPK2a.skate      -AQPNFVRP--------------VCRNRRDLI---SLV----SNADGTPQTLNLGYML--
UPK2a.zebrafish  NNTVNFTVQ--------------PCPVSQS-----------------------WYLL--
UPK2a.salmo      TLVNIFKVL--------------PCRFRRDII---STI----ENNAQFTTSRNLGYQV--
UPK2a.lamprey    -IVGQVAVP--------------GCRVARAVSG--QVVSGNDGTGPGIPVPSGVAYRV--
UPK2b.snake      YFADEFKDI--------------SCRVARDLL---VLD----LDDSQYELITVVGYQVGG
UPK2b.turtle     -HSSKFKSV--------------SCRIARDLV---SMD----IDDGNYKLTTVVGYQVGV
UPK2b.lyzard     YFAGEFDSV--------------SCRVTRDLL---VMD----MDDSQFELITVLGYQVGA
UPK2b.aligator   YYAGEFASV--------------SCRMARALV---KMD----IDDENYKLTTIVGYQVGA
UPK2b.salamande  YYAGQFNTV--------------PCRVSRDVV---QVSSK--AADNSFTLTTVLGYQVGS
UPK2b.Xlaevis    YYTGQMKVP--------------QCRLRRDLE---PVK----MNS-----MRDLGYQVGT
UPK2b.shark      -LPQRYSVP--------------FCNDFSPKPA--------------AVSTPLAYQMGP
```

C_3 C_4 3

```
UPK3a.human       NGTCLW---DPNF-QGLCNAPLSAATEYRFKY----VLVNM-STGL----VEDQTLWSDP
UPK3a.mouse       NGTCFW---DPNF-QGLCNPPLTAATEYRFKY----VLVNM-STGL----VQDQTLWSDP
UPK3a.cow         NGTCLL---DPNF-QGLCNPPLSAATEYRFKY----VLVNM-SSGL----VQDQTLWSDP
UPK3a.elephant    NGTCLS---DPNF-QGLCNPPLSVATEYRFKY----VLVNM-STGL----VQDQTLWSDP
UPK3a.dog         NGTCLS---DPNF-GGLCNAPLSAATEYRFKY----VLVNM-STGL----VQDQTLWSDP
UPK3a.opossum     NGYCSL---NPNF-KGTCNPPLTRATEYRFKY----VLINP-SSGF----VEDQTLWSQP
UPK3a.platypus    DTACMW---DPDF-SGACNPPLAGETGYRFKY----VLVNT-TSGS----VVDQSLWSDP
UPK3a.chicken     DGTCLY---DPNF-LDVCNPPLAPDTTYRFKY----VLVDN-TEGI----VKDQTLWSDP
UPK3a.aligator    DVSCLY---DPNF-LGVCNPPLASDTTYRFKY----ILVDE-TLGI----MKDETLWSDP
UPK3a.turtle      DVTCLY---DPNF-PGACNPPLAQDTTYRFKY----LLVDV-NAGV----VKDQTLWSDP
UPK3a.Xtropical   DVTCLT---NPNF-VGYCNAPLQGNTQYSFKY----LFTD--SGDI----VQSETSWSLG
UPK3a.Xlaevis     DVNCLT---DPNF-KGICNPPLQNNLQYRFTY----VFT---IGDV----VQYQTDWSPP
UPK3a.salamande   NPTCLT---DPNF-SGICNPPLANSTAYRFLY----SLVHN-NGS-----RVANTHWSDR
UPK3a.coelacant   DTMCSNM-------PGICNGPLTPNTAYRFKF----VLLNG-NR------PAAQTPWSSM
UPK3a.spottedga   NPTCVG---EPEA-EAFCNAPLSDGTSYRFKY----LLVNG-TT------TIAETEWSES
UPK3a.skate       DDQCLN---QAPFVSGNCNAPLKENVAYRFKY----AVLNS-STNI----ILNETSWSDP
UPK3a.shark       DTQCLS---GGTAQSVPCNAPLNENVNYRFKY----VARDP-ITFI----LRDETVWSKP
UPK3a.1.lamprey   DTACVN---SIGPSTSVCNGPLVPGMKYRVKY----TLSEE-SPQFP-RTIVDQTPWSDP
UPK3a.2.lamprey   DSKCSV--------AVTCNGPLNAGTIYWFKY----IIGTV-ATHGAIDRSYLESSWSKP
UPK3b.human       DHGC-------HQ-QPFCNAPLPGPGPYRVKF----LLMDT-RGS-----PRAETKWSDP
UPK3b.cow         DAGCLA---DLHQ-PRYCNAPLPGPGPYRVKF----LLTNS-RGS-----PQAETRWSDL
UPK3b.mouse       DFGC-------YQ-RPYCNAPLPSQGPYSVKF----LVMDA-AGP-----PKAETKWSNP
UPK3b.elephant    DARCLA---DLQQ-SPYCNAPLPSPGPYSVKF----LLMDT-EGS-----PKAETRWSDS
UPK3b.dog         DPGCLA---DFYE-PPYCNNPLPSPGPYRVKF----LLMDA-RGS-----PQAETRWSDP
UPK3b.platypus    DPGCLR---DPGR--EYCNAPLPAPGPYRTKF----LVMDE-ASH-----PKAETQWSDP
UPK3b.opossum     DTNCLR---NLSQ--EYCNAPLLAPGPYRVKF----LVMDN-NGQ-----PKAETWWSDP
UPK3b.Xtropical   TAQCND---N-----TNCNGPLPDPGPYRVKY----LVMNN-NA------LVSQSLWSQQ
UPK3b.Xlaevis     TVYCTD---N-----TYCNAPLPDSGPYRVKF----VVMNN-NA------LVSSSLWSGL
UPK3b1.Xtropica   DVNCSN--------PLFCNGPVPSRGTYRVRF----VVLNG-TV------METGTRWSEV
UPK3b2.Xtropica   DEKCNE---------SFCNGPLTSPGPYRVRF----VVLNN-TG------MVAKTNRSDL
UPK3b.chicken     DTACHG--------RAPCNGPLPSPGPYRVKF----LLMGC-GG------PKAETKWSDP
UPK3b.shark       ETTCRT---------ETCNVPLNNSKSYSVKF----ILINP-SLQNS-PNVIAQTNWSSF
UPK3b.aligator    DAFCRN---DNSQ--QHCNGPLPNPGPYRVKF----LILDS-NG------AKAETRWSER
UPK3d.coelacant   DTSCPQ---SIGE--SDCNGPLPDSGSYRARF----LIVDS-QSGN---MLKAQSQWSKS
UPK3d.1.spotted   EGNCTS---------ANCNGILPAGSTVRVKY----VLVDP-ASRN----VTTETYWSQN
UPK3d.salmo       EGNCTN---------TNCNGILPPGSTVRVKY----ILIDP-VSRG----VVSESKWSYP
UPK3d.trout       EGNCTN---------TNCNGILPPGSTVRVKY----ILIDP-VSRG----VVSESKWSYP
UPK3d.zebrafish   DGICTG---------INCNGVLPVGSIVSFRY----LLIDP-SNYT----VVNMTNWGGP
UPK3b.skate       EDPCTT---------PNCNAPLNAGSLVRVRY----VMINP-LATT--NNVIAVTKWSNP
UPK3d1.salmo      DGNCST---------PNCNGILPVGSTARFKY----VLINP-ANKT----VVAESLWSNN
UPK3d1.trout      DGSCST---------PNCNGILPVGSTARFRY----VLINP-ENKT----VVAESLWSNN
UPK3d.spottedga   EGNCST---------PTCNGILPAGSTASCAVLGLAVVSQL-QDQL----VKGALLWAQQ
UPK3d2.spottedg   EGLCST---------SNCNGILPAGSTVRAKY----VLLDP-GSKQ----VVSESQWSSP
UPK3c.human       DTHCQPT-------KIGCNHPLPGPGPYRVKF----LVMND-EG------PVAETKWSSD
UPK3c.mouse       DNNCSLE-------SQGCNSPLPGAGPYRVKF----LAMSA-EG------PVAETLWSEE
UPK3c.dog         DTSCSPT-------KRGCNHPLPGPGPYRVKF----LVMSD-KG------PVAETEWSNE
UPK3c.cow         DTRCSPR-------TRGCNRPLPGPGPYRVKF----LVMSD-RG------PMAETEWSSE
UPK3c.elephant    DTRCSLT-------TKGCNHPLPGPGPYRVKF----LVLSDDRG------LVAETEWSGE
UPK3c.opossum     ETNCTR---------SDCNKPLPGPGPYRVKF----LVMNT-NG------PVAGTNWSED
UPK3c.platypus    EANCTS---------KSCNGYLPGPGPYRVKF----LVMNE-RG------PVAETDWSDN
UPK3c.lyzard      EVQCAS---NRAR--PDCNGPLPRPGPYRVKF----LAINP-DG------VTAESEWSEE
UPK3c.chicken     ETGCAD---NISV--PNCNGPLPGPGPYWVKF----LALNG-SE------PTATTEWSGP
UPK3c.turtle      ETGCVS---DTTR--PDCNGPLPGLGPYRVKF----LAMSP-VTG-----PTAETRWSDP
UPK2a.human       -------------------TNLVPGTKFYISY----LVKKG-TAT--------ESSREIP
UPK2a.mouse       -------------------TNLTPGTKYYISY----RVQKG-TST--------ESSPETP
UPK2a.dog         -------------------TNLVPGTKYYISY----LVRKG-SST--------ESSREIP
UPK2a.cow         -------------------TNLAPGTKYYISY----LVTKG-AST--------ESSREIP
UPK2a.elephant    -------------------TNLVPGTKYYISY----LVKKG-TST--------ESSKESA
UPK2a.opossum     -------------------TNLIPGTKYFVYY----SVEKG-TAV--------ESSNKVQ
UPK2a.platypus    -------------------TGLRPATTYVXXX----XXXXX-XXX--------XXXXXXX
UPK2a.salamande   -------------------TNLTANTTYTASY----QSNGV-TIGLPTN-----------
UPK2a.Xlaevis     -------------------QNLQPGAIYTTYY----AVDGS-NIP-----------SIT
UPK2a.Xtropical   -------------------QNLQPGTNYMATY----SSGGI-SGP-----------SFQ
UPK2a.coelacant   -------------------GNLKAGTQYDFRY----TIDNT-TNV-----LKSNI---IQ
UPK2a.skate       -------------------EMLQPSTTYNVYL----RAGTI-RSNMLG-----------
UPK2a.zebrafish   -------------------GNLKNGTTYSMSY----KIGND-TSSVL------------
UPK2a.salmo       -------------------TNLTTGSTYRFQY----VVGAE-KSNILE-----------
UPK2a.lamprey     -------------------TGLTPSTSYSVVL-RHSTLGLQ-SVPGI------------
UPK2b.snake       EF-CRQTK------GPFCNQALKPSTFYRVNF----FFLDD-KSV-----IRAHTDWSTA
UPK2b.turtle      EV-CEKTK------GPFCNQALQPSSVYRVNF----FILDE-KAV-----IRAHTGWSDV
UPK2b.lyzard      EF-CRQTK------GPYCNQALKPSTYYRVNF----FFLDN-KSV-----IRAHTDWSTA
UPK2b.aligator    EV-CENTK------GPFCNRVLKPSSFYRVNF----FVLDE-NAV-----VRAHTDWSDP
UPK2b.salamande   EV-CTSVK------GLYCNQVLEPGTPYRVNF----FILDA-SNV-----IRAYTDWSDI
UPK2b.Xlaevis     EN-CTEVS------GPFCNQFLQPGTSYWVNF----IILDE-TDT-----PRAYTGWSEP
UPK2b.shark       NVACID---------GSCIEHVLPGQRFRVRY----IIYSV-SEE-----ALVTTKWSAP
```

```
                        4                                          5
UPK3a.human     -IRTNQ-LTPYSTIDTWPGRRSGGMIVITSILGSLPFFLLVGFAGAIALSLV--------
UPK3a.mouse     -IWTNR-PIPYSAIDTWPGRRSGGMIVITSILGSLPFFLLVGFAGAIILSFV--------
UPK3a.cow       -IRTDR-LTLYSAIDTWPGRRSGGMIVITSILGSLPFFLLIGFAGAIVLSLV--------
UPK3a.elephant  -IRTNR-LTPYSAIDTWPGRRSGGMIVITAILGSLPFFLLLGFAGAVLLSFV--------
UPK3a.dog       -IRTNR-PTPYAAIDTWPGRRSGGMIVITSILGSLPFFLLVAFAGAVVLSLL--------
UPK3a.opossum   -IRTNQ-ISPYLEIDTWPGRRSGAMIVITSILSTLVFFLLVGFAAAVIFSFV--------
UPK3a.platypus  -IRTKR-TLSWSRVDTWPGRRSGSMIVITSILSSLMFVLLVGLAAAVTCRV---------
UPK3a.chicken   -IKTRK-AKLPMKIDIWPGRRSGSMIVITSILSVSVFLLLAGLLASVFSALV--------
UPK3a.aligator  -IKTNS-VKKSSTIDTWPGRRSGGMIVITSILSVLIFLLLAGLFASVFFAVM--------
UPK3a.turtle    -MKTRR-VKQSSTIDTWPGRRSGGMIVITSILSTLMFILVAGFLASLYFIVM--------
UPK3a.Xtropical -ITTVN-GKASSTIDTWPGRRSGGMIVLTSILSTLMFFVFIAYVIGFAYSIA--------
UPK3a.Xlaevis   -ISTVN-VKSSGTIDTWPGRRSGGMIVLTSILSTLMFFVFFAYIVGFAYSIL--------
UPK3a.salamande -ISTKN-VKTPDTLDTWPGKRSGGMIVITSILSTLLFFLLSGFVAAANV-----------
UPK3a.coelacant -ISTRK-SKPFEDIDTWPGKRTGGQVVVTTILVILLFLLLCGFVTTLVASI---------
UPK3a.spottedga -ILTRK-ALSPDEIDTWIGKRSGGGIVVTVILSLLLFLLLGAAIFMGVLDVI--------
UPK3a.skate     -IPLLR-VADFALIDTWPGARTGGMVVITTLLVILLSLLLCGYGALLVYACC--------
UPK3a.shark     -ITLLQ-VQDPARIITWPGARTGGMVVLTTLLVIILFLLLCAFVAFL-------------
UPK3a.1.lamprey -VSTKK-SPAASTINTWPGKRTGGMVVVTAVLSTLLFLLLAALLLVVIFKACS-------
UPK3a.2.lamprey -IRLNK-AGELNAIGVTPGPXSGGMVVVTVILVVLLFIAVDRARPALGQ-----------
UPK3b.human     -ITLHQ-GKTPGSIDTWPGRRSGSMIVITSILSSLAGLLLLAFLAASTMRF---------
UPK3b.cow       -IALRQ-GKSPGSIDTWPGRRSGDMIIITSILSSLAGLLLLAFLAASSVRF---------
UPK3b.mouse     -IYLHQ-GKNPNSIDTWPGRRSGCMIVITSILSALAGLLLLAFLAASTTRF---------
UPK3b.elephant  -IALHQ-GRAPGSIDTWPGRRSGGMIVITAVLSSLAGLLLLAFLAASTVHF---------
UPK3b.dog       -ITLHQ-GKAPGSIDTWPGRRSGDMIIITSILSSLAGLLLLAFLAASTVHF---------
UPK3b.platypus  -ITLKQ-GRDPSSVDTWPGRRGGSMVVIASILSVLAGLLLLALLTAAAGACTV-------
UPK3b.opossum   -ITLNQ-GKDPRSIDTWPGRRSGCMIVITSILSTFAGLLVIAFLIASTVQF---------
UPK3b.Xtropical -ITLLT-GKSSSQLDTWPGRRSGGMIVLTSILSVLMGILTLCLFAAFFVGC---------
UPK3b.Xlaevis   -ITLRT-GKNPSTIDTWPGRRSGGMIVLTSILSLLMGILTLCLIAAFFVGC---------
UPK3b1.Xtropica -ITMHI-AINSSTIKTEPKRPSSGMIVIITILASLLFILLVCLIAAVSLGS---------
UPK3b2.Xtropica -IRLPI-GINYTTIDTWPLSRSGSMIVITTILSILLAVLLACLLAALCSE----------
UPK3b.chicken   -ILLRR-ARSLSTIDPTPARRSSTAVVIAAILASLGAALAMAVLGAVG------------
UPK3b.shark     -ISLKT-VVEPRNIDPSPRGRSAGMIVITAILSVLLFLLLAFFVAMLLMVC---------
UPK3b.aligator  -ITLKQ-GHRSSTIDTWPGRRSGTMVVITVILSSLVGILTIAFLCTSAYD----------
UPK3d.coelacant -IQLKR-PKDPASIDTWPGKRTGGMVVVTTILVILLAILLLLFIVALITAC---------
UPK3d.1.spotted -ITLYS-STDPVVIFDGIRQRSAGMIVITSILSVLLFLLLLLLIAGLIYAK---------
UPK3d.salmo     -ISLTS-TRSWSSIDEWLGKRSGGMVVITVISSCLLAVLLLLLGAVLLLDG---------
UPK3d.trout     -ISLTS-TRSWSSIDEWIGKHSGGMVVITVISSCLLAVLLLLLGAVLLLDG---------
UPK3d.zebrafish -FNLTT-LLSYQTINDGLSARSGAMVVITTLLCVAVALLLLVFFIMLCVSC---------
UPK3b.skate     -IQLEN-AVDPNIIDT-STRRSGAMVVITTILSILLFLLLVLFIVMLAA-----------
UPK3d1.salmo    -ITLYS-LKDPEKIDNGFAGRSAAMIVITAILCSFLALLLLLLLIMLIYVL---------
UPK3d1.trout    -ITLYP-LKDLESIDHGFAGRSASMIVITAILCSFLALLLLLLLIMLIYVLCC-------
UPK3d.spottedga GLVVVD-VMPSSSIDDWTWKRSGGMVVITVITSCLLAILLLLVAALLLGG----------
UPK3d2.spottedg -ISLIA-LSDSAGIDEWIGKRSGGMIVVTSILSSLLAILLLFLLTAFVLRS---------
UPK3c.human     -TRLQQ-AQ---ALRAVPGPQSPGTVVIIAILSILLAVLLTVLLAVLIYTC---------
UPK3c.mouse     -IYLQQ-AQ---TFREAPGSQGKGTVVIIAFLSILLAILLVVFLVLVISAC---------
UPK3c.dog       -THLQR-AE---RLQAAPGPQSTGTVVIIAILSVLLAVLLTALLALLIYTC---------
UPK3c.cow       -TRLQQ-AE---VLQAAPGPQTAGTVVIIAILSVLLAVLLAALLALLIFTW---------
UPK3c.elephant  -THLQR-AE---VLQASPGPQTAGTVVIITFLSVLLVVLLTALLALLIYIC---------
UPK3c.opossum   -ITLRK-PVEFS--ESRPPSKSAGTIVIIAILSILLSLLFLALVALLVYTC---------
UPK3c.platypus  -IALRE-AKDPG--DPNPTRRSTFMIVIVTILSVLFALLLGALTGVLIQAC---------
UPK3c.lyzard    -IALVQ-AQSPETIDVSPGRRSASAIAIASLLSILCAVLLAALIAALVYKYT--------
UPK3c.chicken   -ITLKT-AREPQSIPGMGGARSGAMIAITAILSVLLAILLAALLATLCS-----------
UPK3c.turtle    -ILLKA-GKDPATIDTWPGKRSAGMIVITTILSILLAILLACFIAALTYRC---------
UPK2a.human     -MSTLP-RRNMESIGLGMA-RTGGMVVITVLLSVAMFLLVLGFIIALALGSRK-------
UPK2a.mouse     -MSTLP-RKNMESIGLGMA-RTGGMVVITVLLSVAMFLLVVGLIVALHWDARK-------
UPK2a.dog       -MSTLP-RRQAESIGLGMA-RTGGMVVITVLLSVAMFLLVLGLIIALALGARK-------
UPK2a.cow       -MSTFP-RRKAESIGLAMA-RTGGMVVITVLLSVAMFLLVLGLIIALALGARK-------
UPK2a.elephant  -MSTLP-RRKMESTGLGMA-RTGGMVVITVLLSVAMFLLVVGFITALALGAQK-------
UPK2a.opossum   -MATLP-RRKVETLGLGMA-RTGGMIVITVLLSVAMFLLVVGLIVALALGVHK-------
UPK2a.platypus  -XXXXX-XQKAELLVVGMA-RTGGMVVITVLLSVAMFLLLLGFIVALVLGSHD-------
UPK2a.salamande -FTTVQ-PTNYTAM-PEVFARSGGMVVITVLLSIAMAILVIALILTFVMGRKK-------
UPK2a.Xlaevis   -FST---RSVSQTV-PDIMARSGGMVVITVLLSIAMFVLLVGLIAVLVIGRK--------
UPK2a.Xtropical -FST---RTVYPAV-ANIMARSGGMVVITVLLSIAMFVLLAGLIAVLILGRK--------
UPK2a.coelacant -ITTSL-VTSNVLIDEGFKLHSGGMIVITILLSFAMLFLIIGVIVVLVLGNKS-------
UPK2a.skate     -VTTIS-PVDYRTIDLGFG-RSGAMVVITVILSIAMLALIIAFIVVLVLSK---------
UPK2a.zebrafish -TNTTTNVNDYQQIDTGLRARSGAMVVITVILSLAMVFLLVGIILVFFFFSG--------
UPK2a.salmo     -VSTRQ-VKDHNQIDSGLPACSGAMMVITVILSVSMFILLVALIFTVAHSLGGD------
UPK2a.lamprey   -LSTANARTPRSAVDAEAFSRSGGMVVITVILSVLIFLLLAILVAALLLGNKN-------
UPK2b.snake     -IQTRN-VTNYESADVMFEGRAGGMIVITILVSVGGAVLLVALIVAVALSSKK-------
UPK2b.turtle    -IQTNN-VTTFMAYDGSFIGRAGGMIVITVLLSVAMFVLVVGLIVAAALGGKKS------
UPK2b.lyzard    -LQTRN-VSDHESADVMFGGRAGGMIVITILVSVGGAVLAIALIVAVALSNKK-------
UPK2b.aligator  -IQTNN-VTSFSAYDGSFEGRAGGMIVITVLLSVGMFLVVVGLIVAAALGGRKS------
UPK2b.salamande -VTTLN-VTNNAQLDSGLSRRSGGMVVITVLLSIALFMLVPALIATLVVGREKSPL----
UPK2b.Xlaevis   -RTTRQ-VRRLDAVDLGLSGHSGGMVIITVLLSVSVFLLLLGFMAVVVVSRAPNLFTSES
UPK2b.shark     -IATRDDPPSYFSIDADTTPRSGAMVVITTVLVVALFLLLLGFVSMLAARSKFAR-----
                                           __________________________
                                                      TMD
```
