# Supplementary material for: The Tetraspanin-Associated Uroplakins Family (UPK2/3) Is Evolutionarily Related to PTPRQ, a Phosphotyrosine Phosphatase Receptor
Source: PLoS One. 2017 Jan 18;12(1):e0170196. doi: 10.1371/journal.pone.0170196 (PMC5242461; doi:10.1371/journal.pone.0170196)
Supplement: S1 File — The similar region and transmembrane helix are highlighted in green and red, respectively. PTP catalytic domains are underlined. Exons are displayed in alternate colours. Amino acids in bold red colour indicate they are split between adjacent exons by a phase 1 or 2 intron. The C1–C4 cysteine residues and the CNGPL motif are highlighted in yellow. (PDF) [file pone.0170196.s006.pdf]

PORIFERA

Sponge XP_003383454.1 curated by ptotein profofiling [Amphimedon queenslandica]

MFFIIDILIKNFYLGFAPDVINFTVNSGNSTSLALSWQLRYPLVPPDMYYIAYNYTELSGANPRSDNSTIMIDEDDVTTTTDYI
LYTLDDLLPFTSYYVTLFPQYGEEEGEGLSDSGNTGEGMMSSQVNDTNVDIDRDNMRLNISWAPPSRPAGNILRYEVTLSIRGK
ICLTINTTELSVIAEFKFNVTYTIRITPFNSFGSGTTRELDMFATPEGAPTPPLNFTLTELSLSSLKASWEEPSTLNGVLANYT
VYCNLSSLQFYSQQLMLSFNAPPTTVDPDSTTANITGLYPFTNYDCYATASTGGGESSRSNNDSARTSEEEPAGPPEGFNVTDV
TATSVSLEWRRPSVPNGVILHYFLQYSAYNVTIPVTFNTSSDEYSVTSYTVESLNEYTNYTFTISAVTSGGAGPLATTSTRTSE
AVPGTNLDGFNTSSMSLRITWTELLEDDQNGVIIGYNISYFSLPAVGQPINNFTSDTSYNISGLDVYTDYNVSVAAYTSAGTGP
FDSVIRRTDSTVPTSPSDVSYINISSTSIEVSWNPPTDFNGPNEGYVITYIRLESDTESMSTNRLTGTSFVIENLEKYEQYSVT
VVAFTDKGPGASSDVLSVLTDEDLPGPPSNVSTMSTNTSISITWSPPLDPNGLLLSYSINVTLNSTYAQYLSFDVMTMSVSQNI
FSYTLFDLLPFAGYDISLQASTSVGLGTPAIETETTLQAAPAAPVANPMASPVSSTAVNVSWLPPNLSNWNGLITNYTIEYRTN
DEYIRPSIEVTATLSSFANSDDPRAATTPLQLESIIIPSLHEFVNYSFIITLSNSIGQISSLPVHIVTLESTPSGPPLDVSVIV
DSPSTARISWSPPMYIDRNGIIVNYTVRIITTVRGTIRETNISNVSGNYYDASDLPQFASFNVTVAAATSVGLGPYSPSVSNMT
FEGVPSAPDPVIVKRRNDTAIQVNWTRPAEPNGIILGYLIYYIGTKNNTGTEYSNINVLIIINVTDPNTLSYLITNLLADTQYF
INVTAYTSAGLGAVGDGDSILLPTGRPPTVPPNVTIGTPPTGGTSDPTTATTIRIEVTIPEELNANGPLTRIRILIRIFLSRND
TISTWYESQKFPNSVAPPWQATQLPLNQGNRRKRQAGGETVAETIGTNNSCGPNDIVCNGPLKPGTQYQFKYRVYNSDDDDSYV
ESQYSGPIRTGDPIAEENNTGTTIVIAVVVVLLVIILLIAILVIVVIIVLKRRKRKAYSFAASFNGKEELITAGEGKGATLSKA
IQLTPTDPTIVPLTENTIVNPTAVRTASVKATPNSRKVYLRDFPSYVEDMLNDAGFKFSEEYEKVSAVGLDHSKDASLLPENRA
KNRYTNILAYDHSRVKLESIDDEPGSDYINANYIPGYRMRRAYIATQGPLPSTFDDFWRMTWEQNSHVIVMLTQLVERGRTKCH
RYWPGAQPEVYGEINVDMLSETEKSDWIVRKFKITKEKRSRTITHYQFVSWPDHGVPDEAGPALDFVREVHEVASSAFGPVIVH
CSAGVGRTGTFIALGTLLQHIKDHDWVDLFGLASEMRQHRNHMIQTEPQYVFIHKAMVDACKSSMSKFGGGIKVLPSFPIYSNI
GPASQLSDFYGDMLSEEASYQYQKTSGNGKTYDESTF

PROTOSTOMES

Nematode.2 CeNTP10D C. elegans. F44G4.8a DEP-1 NP_001254192.1 [Caenorhabditis elegans]

MIRWKYELHSLIWLFLVLHLSKCQSDSLTTSAEQHELFAIKKDSLSPWSQILVSLPRRHPLYQSFAAKIQDVTENISDEVRDSN
KTFVSSDDAPYSIRIHALRAGHRYSIAIHGQKDGSTSLIKEESVVMDPRAPDFRSMDSDIQVAEHNITMRTIKNDSYLQDSFSI
EYRQINPDKKFPVLQILDIPEQKNLEFYLGNLNSGFDYSVRVIAHKDGMSSRPWISTLTTKPSPLKEVNINQNAGSCVEVSWQN
DEFSGADFYTIQYSLQSTPNNSTNMTIPSTESSISICDSMLQGEAYQIIATVQKGGQVSEPLITKFQLRPLPPIDFRVRADLKR
GKYKLLAELPTSSKIDKCQITVAGDEAERSVNYANIEQTKSGHKICWFNFALSPGERFDFSISSMANESASQKLQKSIVLTPAF
DFNAFGLTLQESNGGIELIWPKSEVFMTRVKDIWNKVVGAESLLNMRITPIGNNDETDKTLKFETSPKNIDPVFAKNLVKGACY
RVQLFTVTKTGIISETRHNETIRMSSPAVNVSLESVTRSSATLRIVFSTHHDSTSISNCQMHIVVRDMNGKSVFDKRMQLTATF
APLLNLDGLSPFHKYTVNTQIICGSGSSETPQCPAATRTMRQLSFSTRQDKPAAVQDLKVEPLNSYSVMLTWLPPALPNGILTH
YAVNVTKIGSDETRTIDVGVSSNRSDHTVQVVIDELFGGHTYSFSVRAVTEAGFGENSPVVPTVSMPLMAPPVPTVAPMIMKES
VGSHNMIVRFPTTMFDNRNGEIKQFAIIVSETTADESINRWIESDNGTYTWQQVQRFDVWPSYVAKLQDIQKVKQDVDVSIFEE
LGEDETCLEVRADRICNGPLRSASKYRVRIRLFTSPTLFTDSPPSQVMTTGSATPAIPLLTVVAVLIVIAFVGIVGTIFLFFWN
RTKKARLAAAAFKNGPSKEKESQWEALKMMMAERAADCLAKLGLDATTPPPSSTTSSNSPTSTSTTMTDCGSNPHLGAPNAGGH
RRTRSLRERTGVEHRLERLSSGPVHRTPLYTVVTGANTNKSRPVRIEDFADHVRLMSADSDFRFSEEYDMMRNVGVGQSVAASE
LPINRPKNRFTNIPSYDHSRVKLSNPNNIEGGDYINANYVPGFSSRREFIAAQGPLPTTRDHFWQMTWEQQCPAIIALTKCVEK
GRDKCHQYWPDHENVPVLYGDIEVTIVAEKEFDEFVIRDIRLEKSGPDGRVTRFVRHWHYMAWPDFGAPSHPNGIIQFSRMFRH
HLPHSPHNAPTIVHCSAGVGRSGTFISIDRLLQSSSFGDPIDVFGTVCEMRYERCQMVQNEQQYIFIHYCILQVLQGSSPSPTS
TSTGAHHNAGFVQDGQMIVESGF

annelid gi|443734862|gb|ELU18718.1| CAPTEDRAFT_229306 [Capitella teleta]

MSQPVTNQLAGFGVLAALALICVSRSESSSTTTAAVPTTNFSTNIPPTTQGLEVTEVTTSMVTLVWSYDGSEQLTFVVEYVEKD
ADNATSFVLEQGVTSCVVEGLQAGWTYTFEVSVQLERDMLGEPAYANATTIPGKPINFRTVDNSVGATFFTLTWESAAGSQQDD
FVVVYRKIGSSDVVQVFTGGATSVTLHGLDAGVLYEVKVMAVRGQQLSTPAIITEVTRPPTPRIHNITAVDTKSVTVQYSAGAG
GSQNSFVVTYVNQEGQTNEPVCSYSNTCTFDVSVFGVKPGEHFTVKIKATQDMVSSAWASMSGNTKPLPPSAPKLFERSETTLT
VIGDVPSTNSTVFDGCKASVTPAIQNGPYVFKRNGGYEIRLQNPTPGSKYAINIWTYSREEESAHVSGDFYSMPAQPGVISRHT
GIATTTKQLAVSWGAPNSGQVGGYVAKLYEHGKSVAIESKTLTTNMRQATFHNLTPGQVYDVSVSSYVGTGVYSAFSTIELSTK
PESASNLELKTTTSSTLVVQWDEPSTGFYDEFLVRIQSHGETEKITKSSQSTYEKEFDNLSAGTEFTIVVITVSHGQQSTATIG
IFYTKPERPSNLKKESTTSDSITVKWNAPVSANFDSYRITINSTDHENEVLDSSESSHTFSGLMPAQQYDITLISLLNSLESSM
LSIQVYTNPAAPTDLRILSRNTNMMSFTWSPPTNPGTIDDYKYSITISGAATPEIERSGTINEYSHQLSAGKTYIIEVRAVTNN
QRGLPVSLTFTMIPNDPGNVREMERGQSYLKIEFDAPWDTYDGFEVTWVEKGSGPSSVADLLVTSYNDDSITVEWEKPTGTTVN
GFTISIDPPPSGQSGSKDLTENIQEYTWTGLSAGREYNLIIEVIFADTKSEKVERRQTTKPKRALSLSANYVSTDSISLSWKVD
QSSSQDNFKITYEVTGSSTEWTRETTFTQGQLQYSYLIEGLSAGSTYSISVIAKKSSVQSDPLEITQTIQPKSVRELKAEVIPS
GISLSWLPGFDSTQNSYRYQYQGKNVKLNIVPWVVINSESVDLQNLFPGERYQFYVDAISNDQYSPSNQSTLATTYPLPPTDLT
VDRSATTVSSVRVQWKDDVTRSYITSWDIKIADRGTDNIRRVGSTHERTNLNYEIPNLTAGKNYTVYVYGKSGNQHSRDASTMD
VTAKPVIRSTLSEDTEKTTEDTIAVTYSESKAGVFDHYLFSLLNSSDTVIKQRSNTDRNIRFENLVAGVKYTILARTVSGVEES
MTTQKSITTKPNKPKHKCNQGTDKLTIILIKPSGFVDEYILKCLNEDCNSTEISNVSFEQDFNNLKPYTTYHFEAYTKRFEKLS

NKVEFPCKTGEGPPGPVQEFSYSENSLKPFEIRLTWMEPRNPNGKIIKYHIKYNGIKENQVPHTGSEVDVEPDMHEQLIKGLKP
GFEYTFEIAAETNAIGKKTTLVKTMPIKAPIVMPNSPPPQAITTAISHDKIRIILTNPFLNTNGDVVAFSVFVTTDPNERFMAN
SPLRTWADVKGPSPMASYFAVYKCANLFDGNDQCSSGPARKRRVAQPRNTVEFTVGGDSSCTTNADDYCNGPLDAESTYYVALV
GYTENDLYSSGPSSEPIRTDTAPTNLLLIIIVVVVVLVLAIVAAIGVIVYMRKRSSDNNEDKLVCCLVLIGVKPASPEWPPGSN
NGMVIIPSSKPVKKVDFVQHVASMAADSEFKYSEEYELSLVGKDQPKEASDLPCNRSKNRFINILPYDHSRVKLLPIDDEDGG
DYINANWIPGYNSRREYIASQGPLPSTRDDHWRMIWECNCQCIVMLTKCVEAARQRCDHYWPIDSEAIYYGDLQVQILNETKST
DWSISEFIISKGDQSRRLKHFHYHSWPDMKAPNKTGSIVPFVRLIRAKVQLDGGPLLVHCSAGVGRTGTFIALDHLMQHIEEHD
DIDIYGTVYQMRKHRCMMVQTESQYIFIHKCLEFVLEGGEDDEYANTGAIGTENRAFEGLRSESDLVAEIFFNLPRQALTNFDD
DEGIDVKSDP

Fruit Fly DPTP4e L20894 FBtr0301793 [Drosophila melanogaster]

MDCATRKQQQLRAHHQQQQIQIQTHGRKRQQLQKQRHNHHHYYQNSQQQQKHFVWLVVGILTIFLAQHANAADLVINVPNASSN
ANAFYRIDYSPPFGFPEPNTTIPASDIGKDIKFSRALPGTEYNFWLYYTNSTHREQLTWTVNITTAPDPPANLSVQLRSSKSAF
ITWRPPGSGRYSGFRIRVLGLTDLPFERSYSLEGNETLQLSAKELTPGGSYQVQAYSVYQGKESVAYTSRNFTTKPNTPGKFIV
WFRNETTLLVLWQPPFPAGIYTHYRVSITPDDAIQSVLYVEREGEPPGPAQAAFKGLVPGREYNISVQTVSEDETSSVPTTARY
LTVPERVLNVTFDEAYTTSSSFRVRWEPPRTYSEFDAYQVMLSTSRRIFNVPRAANGDSVYFDYPDILEPGRTYEVVVKTIADN
VNSWPASGEVTLRPRPVRSLGGFLDDRSNALHISWEPAETGRQDSYRISYHEQTNASEVPAPFPVAAESQITTNLTEYTLDSLL
AGRRYLIAVQALSKGVASNASDITRYTRPAAPLIQELRSIDQGLMLSWRSDVNSRQDRYEVHYQRNGTREERTMATNETSLTIH
YLHPGSGYEVKVHAISHGVRSEPHSYFQAVFPKPPQNLTLQTVHTNLVVLHWQAPEGSDFSEYVVRYRTDASPWQRISGLHENE
ARIKDMHYGERYLVQVNTVSFGVESPHPLELNVTMPPQPVSNVVPLVDSRNLTLEWPRPDGHVDFYTLKWWPTDEEDRVEFKNV
TQLEDLSSPSVRIPIEDLSPGRQYRFEVQASSNGIRSGTTHLSTRTMPLIQSDVFIANAGHEQGQDETITLSYTPTPADSTRFD
IYRFSMGDPTIKDKEKLANDTERKLSFSGLTPGKLYNVTVWTVSGGVASLPVQRLYRLHPLPISDLKAIQVAAREITLHWTAPA
GEYTDFELQYLSADEEAPQLLQNVTKNTEITLQGLRPYHNYTFTVVVRSGSIQGTDFADVSVSTLMRSSAPISASYQTLTAPPG
KVDYFQPSDVQPGEVTFEWSLEPAEQHGPIDYFRITCQNADDAADVSSYEFPVNATQGKIDGLVPGNHYIFRIQAKSALGYGAE
REHIQTMPILAPPVPEPSVTPLEVSRTSSTIEISFRQGYFSNAHGMVRSYTIIIAEDVGKNASGLEMPSWQDVQAYTVWLPYQA
IEPYNPFLTSNGSRKSSLEAEHFTIGTANCDKHQAGYCNGPLRAGTTYRIKIRAFTDEDKFTDTVYSSPITTERSDTVIVAATV
SAVLLVAMVLVVVYCQHRCQLIRRASKLARMQDELAALPEGYITPNRPVHVKDFSEHYRIMSADSDFRFSEEFEELKHVGRDQA
CSFANLPCNRPKNRFTNILPYDHSRFKLQPVDDDDGSDYINANYMPGHNSPREFIVTQGPLHSTREEFWRMCWESNSRAIVMLT
RCFEKGREKCDQYWPVDRVAMFYGDIKVQLIIDTHYHDWSISEFMVSRNCESRIMRHFHFTTWPDFGVPEPPQSLVRFVRAFRD
VIGTDMRPIIVHCSAGVGRSGTFIALDRILQHIHKSDYVDIFGIVFAMRKERVFMVQTEQQYVCIHQCLLAVLEGKEHLLADSL
ELHANDGYEVTKIYLERQPQTKMGTLPIRASLAMAEKLDADLMTNKDEDEDQEQQQQQLQLATEVKPKGSNDDEEDEEDDDDD
DDDQQPLNNETTATLSSASCSSSTHDVHVVLQEAIEKPKQEQERICAGTQSHADTESDNTDSDDDDEDGDGKVAKDGAVADEDG
WWY

Fruit Fly PTP10D M80465 FBpp0303419 [Drosophila melanogaster]

MLYQLSKATTRIRLKRQKAVPQHRWLWSLAFLAAFTLKDVRCADLAISIPNNPGLDDGASYRLDYSPPFGYPEPNTTIASREIG
DEIQFSRALPGTKYNFWLYYTNFTHHDWLTWTVTITTAPDPPSNLSVQVRSGKNAIILWSPPTQGSYTAFKIKVLGLSEASSSY
NRTFQVNDNTFQHSVKELTPGATYQVQAYTIYDGKESVAYTSRNFTTKPNTPGKFIVWFRNETTLLVLWQPPYPAGIYTHYKVS
IEPPDANDSVLYVEKEGEPPGPAQAAFKGLVPGRAYNISVQTMSEDEISLPTTAQYRTVPLRPLNVTFDRDFITSNSFRVLWEA
PKGISEFDKYQVSVATTRRQSTVPRSNEPVAFFDFRDIAEPGKTFNVIVKTVSGKVTSWPATGDVTLRPLPVRNLRSINDDKTN
TMIITWEADPASTQDEYRIVYHELETFNGDTSTLTTDRTRFTLESLLPGRNYSLSVQAVSKKMESNETSIFVVTRPSSPIIEDL
KSIRMGLNISWKSDVNSKQEQYEVLYSRNGTSDLRTQKTKESRLVIKNLQPGAGYELKVFAVSHDLRSEPHAYFQAVYPNPPRN
MTIETVRSNSVLVHWSPPESGEFTEYSIRYRTDSEQQWVRLPSVRSTEADITDMTKGEKYTIQVNTVSFGVESPVPQEVNTTVP
PNPVSNIIQLVDSRNITLEWPKPEGRVESYILKWWPSDNPGRVQTKNVSENKSADDLSTVRVLIGELMPGVQYKFDIQTTSYGI
LSGITSLYPRTMPLIQSDVVVANGEKEDERDTITLSYTPTPQSSSKFDIYRFSLGDAEIRDKEKLANDTDRKVTFTGLVPGRLY
NITVWTVSGGVASLPIQRQDRLYPEPITQLHATNITDTEISLRWDLPKGEYNDFDIAYLTADNLLAQNMTTRNEITISDLRPHR
NYTFTVVVRSGTESSVLRSSSPLSASFTTNEAVPGRVERFHPTDVQPSEINFEWSLPSSEANGVIRQFSIAYTNINNLTDAGMQ
DFESEEAFGVIKNLKPGETYVFKIQAKTAIGFGPEREYRQTMPILAPPRPATQVVPTEVYRSSSTIQIRFRKNYFSDQNGQVRM
YTIIVAEDDAKNASGLEMPSWLDVQSYSVWLPYQAIDPYYPFENRSVEDFTIGTENCDNHKIGYCNGPLKSGTTYRVKVRAFTG
ADKFTDTAYSFPIQTDQDNTSLIVAITVPLTIILVLLVTLLFYKRRRNNCRKTTKDSRANDNMSLPDSVIEQNRPILIKNFAEH
YRLMSADSDFRFSEEFEELKHVGRDQPCTFADLPCNRPKNRFTNILPYDHSRFKLQPVDDDEGSDYINANYVPGHNSPREFIVT
QGPLHSTRDDFWRMCWESNSRAIVMLTRCFEKGREKCDQYWPNDTVPVFYGDIKVQILNDSHYADWVMTEFMLCRGSEQRILRH
FHFTTWPDFGVPNPPQTLVRFVRAFRDRIGAEQRPIVVHCSAGVGRSGTFITLDRILQQINTSDYVDIFGIVYAMRKERVWMVQ
TEQQYICIHQCLLAVLEGKENIVGPAREMHDNEGYEGQQVQLDENGDVVATIEGHLSHHDLQQAEAEAIDDENAAILHDDQQPL
TSSFTGHHTHMPPTTSMSSFGGGG

Fruit Fly.Ptp52F-RB FBtr0289985 [Drosophila melanogaster]

MEATNWKQSHRRIHALILILWIAGVQGQTVGSLVAVSESQELAEKVEVTVVSTVYTLGFSISNEADYSIDKVICRNGSGSETEA
NAAHVCENLNPCTFYTSVVSFRSNVSGKPPPSDQTIYAYTEYKQPKTTVTSVVATANTIKVTWQTNDRACVESFGITAKATDYT
KSFQLLNDKSSQTFVNLSACLTHTITLDTRNNASVVVDTDATDVDTQYAEPGDLVMNVTNLASGITMITWGDPSEKNCISNYVF
KWQRNDCGTDQNQDTTTDVPSTETTNEIDYVTTTPMETTPDTPSDEVKCEWTDVSSDGKLREYLLTDLQGCDLYTFQVFINENS
TAKASQTFTSAEKLVSAVYEPSPTAYPTQLHWTWFSPQNHPKCVANYSVTLTGPIQRSENKTMNVITEETFAIFDDLDPCGIYL

VEIVPNQLNGSAGTKYQEQSTVGEDQPSVIQDPVVEAEAYSMEVSWKTPEYADLCIDGYRLSGWMEDDKLVEVEALSITTQNTT
VVFDKNLLACQVYIIQIIPYTKENLDGQLRQVGVETKAAIVDYTKVKLEMKNAGSEFIDLIAFNADYNNSCPTIFALFTCNATT
QVRNPYAERYVEGHSKQGFNASLSPLSPYATYVCKVILYNVAGPSEPVVDADMHTTTYFPEQPESVMLEKSTVSSLLFNWQPPT
YTNGPIKYYQAFLMRHEASYFVPADCAIVEQDTKSETKGDPSVNFTGLAPAVRYMMQVAAQNDFGMGVYTEPVIGITLPAVSDS
VTQLTVLTQGPVNNNAVYEANVTITWKVPCKSNGDIEYFQLAFNGTRNNFAPVSFERRVELDTGNKQGRMSYTETEMQPQFDYT
VEVSVKNRDVEQLSSSVPGSWQSPAGLPTIPSDELIKQMRANVEETSNPTKTAIVRLPADIMTSASGDIKWMALMISQKNCAGV
PHLKYDVSSDWPKVLSYQEAGADGTGDCSLEYQTTEERWHPEPVQRQRRDGEVTSDEEIVFTIGLDKCSEVQKTYCNGPLLPDT
DYNVVVRLFTASGYSDAAVLNFKTKAAIKVTLILVSVCSCLLLAFVLGLTVLWVRKRLAWKRDSGQGIEDPFGNVIAKNFAIFY
TEVAKPEKLAREFKEITVVALELSYSASELGCHKNRYADIFPYDKNRVILDIDAEGSDYINASFIDGHTRKKEYIATQGPKPES
VMDFWRMILQYNVRVIVQVTQFREGNTIKCHEYYPYNVRGLTVTIKSKEVLELYDRTELTVVHDKYGLKEKVIHYYFKKWPDHG
VPEDPMHLIMFVKKVKAEKRPSYSPIVVHCSAGVGRTGTFIGLDLIMQRLKSESKINIFETVKKLRFQRMKMVQTQQQYTFLYA
CTYELVKHKIPRAALKMDGRPKSVTVPAIPSPKKVSFPDVDVGSEYVSSAPITDLDDGRPIVQLPSRFSGLRRNSPPGENDNPT
SSSNM

DEUTEROSTOMES (INVERTEBRATES)

SeaUrchin1 gi|390362950|ref|XP_790935.3| XP_011667085.1 [Strongylocentrotus purpuratus]

MEGNRPCRHLGLSILGLILICTLQDSVAIDAPDIVTVENEGNTDSLKVTWTPPTGATDQVVTWSPADGSGSDTSFGLSGTATEH
TIPGLTPGTLYNVTVTAQNGTDETSDDSEQGRTKPSPVNLTESGATNDSISAEWTKPSGAIDQYTFSCSEGTEDPSSPITDVSS
NMYEVSCVGLSAAGADYTITVTSLSGNEISNESTTTITALPNMVHIEAGDATNSSFVASWNHPEGEMDSFQVFCYENDTAPEEI
SDVGSNSTYMYEVTCESLPTAGTGYQFSVISISGSKNVTSETVLYTSKLGASFGIRLYYFHTSSCNIWLILGPFCNISLVPMQH
NPVTHLQPLHVHLRQHFITLPNPVELTASDSAVDSVRATWLKPAGEVTSYEVQCSNGTVVPPPVQTGDGPFTASCEDLPTPGDE
YTMNVTSVSNGKSSDVKTIALRALPEAVTLNEERGMVTTTTIAASWTMPSGIVEYYDVFCLVGDPSPVMGNPSGNLTASCVNLT
MPGDDYNISVTAVSNGQRSETDTITITALPLSANLTAGNSTINSVSAMWPYPGGLVDVFQVNCSNGTESNVTVLVNQTNPDPSY
LVSCDGVWNPGDDYTMVVTSVSNEQENSASIMLTALPESVMLNEERDRVNTSTITASWTMANGIVDYYQVSCSNGTASPAQVNP
SEDLEASCTRLSTPGDNYTISVTTVSNGQNSAVSTITITALPSQVSDLAESGATTSSVSATWTKPDGVVSSYTVTCPDGSIQTV
PEDNDGSTHNVTCSILPDPGPGGDHIIMVVTLSGTKASDPATVTITALPSQVTDLAESGATTSSVSATWTKPDGVVSSYTVTCP
DGSIQTVPEDNDGSTHNVTCSILQDLGPGGDHIIMVVTLSGTKASDPATATITALPSQVTDLAESGATTSSVSATWTKPDGVVS
SYTVTCPDGSIQTVPEDNDGSTHNVTCSILPDLGPGGDHIIMVVTLSGTKASDPATVTITAFPNSVVLREDGVTTRQITVQWVD
PVGEEDSFIVDCGDDGSNDITLFPGDATDNTFEATCNTSAAGAPYDITVTSVSGDKESPVTGTFTAATPDGVTFEEGSASTTAV
TVTWIALARADGFRVNCSEGTPSHEGTPSPDSPLPGSATEVTCNGVTPGGNHSVSVITLENNGESPAAVMYVVAVPLSADLAAD
TSTINSVSAMWPYPGGLVDIFEVNCSNGTERNATLAANEDLNSSYVVSCDGVWNPGDNYSMVVTSLSNGEENSASIILTALPVS
VNLTAGGSTTSSVTATWDIPGGIVDEFEVECSNGTASTPGNGSDQDYLASCDDVDSPGDNYTMTVKSLSNTQRNSATIVLTALP
ESVSLNEAIDMVTTTTITASWTKPNGIVDYYEVSCSNGDPSPVMGNPSGNLTASCTGLSTPGDDYTISVTSVSNGQRSETDTIT
ITALPEAVELSEGESNTTVISATWTVRNSVVDTFNITCSDGTASPPSISVDSSQAGQQLTAYCVGLPTSGEQYDLSVVAISKEK
LSAVSTVGVFALPASVESIAVTEPTTTTVDVQWNLTQCSECVYNYFLLTFQPDSQEAIRVDYMAGVNEYSSQVSGLIQGRSYNF
TVVSVSGVGVEDATLKTSEEKSVDQRTGDADKRLHGSIIDIVXXXXXXXXXXXXXXXXXGTRASSLINLVAVPEAVGNISVTQY
ATTTADVQWNLMQCSDCIYNYFLLTFEPDSQEPIRVDYTTGVNEYSYQVSGLTEGRNYNFTVVSVSGVGVQDATLKTSEEESVD
QRTVPATPSDLSIVPGQRELNLVWAYQGDADNFTITVTPNQGIAMFNGDFNDPAAEVTGLTPGTMYDIAIVTASGDERSEPITQ
TNRTLPDKPSPVGNPQAEAVDKNTITLTYESPLEPNGDITGYRISYIGTRDGNTDHEFSDFYSPPVLTVTYNDLYPGFSYTFTI
IAVNDGGFESDPALTEPVETPQEEPSPVPEDYPYEANTVFSETSTTSFAVVLPDDLFSHENGELLTFAVIITIDDNDPTVSSTE
LTYAARAENAYITAIEIPYPYSPSFGSNRRRRATDPPGTVVIGDGTCAGSQNEYCNGDLDDNTEYYYAFRAYNGMGNVTSSTFG
PVKTVKDNTAGIAAGVSVSLIIIIVAVVVVVVFLKRRQPKEPSPRPSLEGRENQGYDGPDHEMRPTRRKTGGSMHRKSHSKPIV
LNKFEGHYGRMKADSDFRFTEEYDEIRLVGKDQAIVSALEMVNRAKNRFTNILPYEHSRVKLAALADDSDTDYINANYIPGYNS
PREFMACQGPLPGTVDDMWRMIWEKKTSIIVMLTQLVEKGKIKCHEYWPADYNPVTYGSIQVSVQALQQYDHWVIREFSISQGD
EIRKLTQYHFMSWPDHGVPDKTWTMLDFVRTVREAIQKTASDRPIVAHCSAGVGRTGTYIALDRLMQAMQENDYIDIFGIICEM
RMQRNHMVQTEKQYIFIHECVMDLLRRGEEEDTGESIYVNLPSNGFKDPDDVNKNLLDQAV

SeaUrchin3 XP_792888.3 SPU_001461 LOC588094 [Strongylocentrotus purpuratus]

MEGGPNAITQLTSSLTTSTNITLNWNSALGVVSYYEVAYDPPNGNSPRRTDVRADEPRTLTLSGLFPDSQYTVTVVTWSGTGED
ATSSTPFSVMFDTAPILPLQVIVRFVNETEAQLTWGLVLGEDTYIITVDDSGANPIPSSETVPEGETPLSVRKGLTPGSLYTAN
VQTTTATGPDQQFRTSPYPPLDLDVADSTPTSIDVAWAPPRAGGVFDEYILSYSIGDRIRTEVGRFAPDVTTATINNLNTATTY
NIYLVTEANDGGFPQSSDVITREGRTEIPAPGEIVIINRTPTSFRFQWGPSIVPDVTYITTYSPSDGSKLDTLSIRQSDVTGAI
PGRLYELTVFVLPGDQQETGLVRTLPSSPGLLAVTAVDYVSVTLTWVPGTGEYTGFIISYKPTEGITYMDVEEVDQDVRTWKVE
GLSPDVSYDFQVVAVSGEHNTTYSEPTRRINTTTLSIAADAFVVLDYNSTSITVAWRDTNTNIIVNIEPQDDEARNVNVGDRPV
HTFTGLNPSTTYTITSLSQSQNLVTQVRTNPASPSNLEVSKTSTSFSIEWEAAESEVSQYKVDVKCADMSCSFNDVIYPNERLV
YELEGLVYLKEYLVTVSSCLDMDGQFAEQMGDEPLESSIITDDVPDDEIVVQETTTDNITIFYATGNRRGFVEAIYLAGTPAQV
GSNSFVTGAVVKKTFDGLVPGTLYTIRITELTNTIIPRTVNVRTRPIAPSAIFGSPTSTVIPIFWFASPGSISFYEVTYSPPDG
TTPAVSRIDASGPLSLDIVTLLPQKVYTVFVRAVTGVDNMPSISKAVSGMFETLPAQPGEIILRRITTDTITYTLSEVPSVLQY
IVQLNIGTDLQSANPHVLPNVEGEFDELTPGQLYTLVLTVPGTQGDLATIQVRTDPNPPGSVSVELTSVPYSSLLAEWEAPPAG
SLSGYEVSVRPADQVLTTMSEVVDSSTTELLLDNLLPEVNYTVEVRTISAGEGFSSTSDPTEALGRTAPFPEAILLVTGYDSTT
ISVTWRKKEGVDYLAQFIEVGQEIGEDFQGLVQSSSSTTTILSFRYTDLVPGRNYELIVRSNDQSEDTIRVSQRTLPSPPMNAA

AAPLSAESIRVTWTAATNDFEFYEVTFYPSADSTDSTAMTIPRDQFSVDIDSLLADTEYVFDIRSVSGSGDSLQKSAAITASAT
TEPIIMRLFSAVENVLLIDFTPIVGIFDDYRYGYRALPSGTITYEPLDSGTSQFGIQGLRSGALYEITLDTRIGSIYTEVDRIQ
ARTRPSAPRLISPVTDFDENSFTLEWLAPSTAEVDFDGFRVIYTPADGALESPLLLDKTRTSLFLTRLEPGTEYMVTLVSVSGQ
GSTQQVSFEEDVSVTTADGVPGGVNIKELTETSIRITWNSALGATYIPTITPDEGTTRILDTEAAIFEELTPGQLYTLIMTQIG
GSSGPPTQQYTLPNAPMDVAISRTTAFELDVSWSPPAEGFYNSFSLTYSRVGTSEVTTIPVPASDAPSVTLPDLLPETSYAVSV
VSVVGTINSQPAETSGTTGRAPEGVPVLVERTTTTISISWGASTDALATGYQLRIRPEGGTSILFNLGQAITTYNFMNLESGTN
YIISLQIFGLSEAPQELPVTTYPQTPGAIQILRTTQTSITFAWGSAGGNFDRYSINLYPDGSQTAFLQDVPRGSDLTVSKNYLA
PGTLYRIEIYAVTGTTRSALRETTASTAMELLVFITIDAFGVAVSWPNAPDFSNYYLEYDPSNGVPPSPVPTTQDSVFLNSLTP
GQIYNFALSSGTPDTGTQSLVRATYVLAPLAPTIEIMILSPTTILIKILPASSGVLDYYRVGVTARQTGLGVGQNGVASFNIPR
EACPEVFITDLVPGGTYDVEVVAVSGETDSAVSSMDVSLETMVAGQLYVNDRTTTSLEVVWGAVTDRTFTNYVLNLFLGNTRTD
FQSQGINEERKVLFEGLLPGTEYSLNLLVEGDAITQSTILTATAPLTPGSLSFLTVEGQEVTLAWPARNSQVDSYELCWTPSSQ
TRSPTADATQELLTLEQSDVEYTISLYALVSVGVGDDVKTTRSDAITRFITLSEALEGELNVTDFDSNSISVEWSEVVSIEFEN
YRLQATPEGDGSPIFAVIPPEDPRMHTFENLIPGVRYTVNNILQGSDPVVTRTVSQRTRPNPVSGLTVVDTTSQSVTVNWQQAT
GSFNDYVVTYTLADEPTTVIYADRIPSDGTRVSVIDGLLPDTSYVVTVYTSSGTDADETTSTEESVPATTTALPSCSIQVTETT
YTEITVQWGSCAVVVNSYELTIEPNSANAPPGNVPIGGNFEYTFNSLVPGQRYIVGIRGTGSTEPYATDSVYTDPNTPDNLVEV
EVSTSTLKVQWDPPVGEYERFEVNLNEQLQGTLRGDQIEAGELPSFTFDALRPGESYSVSIFAVVGSGNLEKRSPPRQNQFTTD
SLDAGQLSVIDFDSFSITVVWGAVEGITFDVYIIQFQPPTGNPIYNRVERTQPRESTVTSLIPGQLQTISLILIDQVDPISVIQ
QRTAPLPPRDLQTQPTVEGSTYSILLLWSMPVPSAIDQYELTYSPADGLSESFIVLEANDEFYTFRELTPSTTYEFRMKTVAGE
GDTRTESSEVEMTQRTLSAPLGQLLLRSKTTVTISVTWGVGNTANTIPEGNYVVRITPGDIADVLVNNEERNVDFTLLNPATLY
SIRLIEQGNDVSSIDVLTDPVAPNSISFESIESTSATVTWNRFVGHDVDNFEVWYRQTSSINDYTLAGEVDGDTESIALSGLDP
SALYDVQIFSKVTSGELTSKSEPAMDSFTTGALQEGVIEFRDKTTTLIEVLFGTAPLSSEVVQYRVTLTSEEGASTSFNVAPTS
EPFSVVTFDGLTPGRLYTVSLQAFGITSTYPTKDVRTFPAPVRGLFPIEIGTTYVLFTWGLPIGDYDSYELSYLKGTAREEEII
SASETNVRIEFLDPNTMYTFNMVTLSAALRSEASDQLFTTTSLAVNEIAVRDVSTTEIEIVWGPEEGVDFWTLTTVPAVSSNIL
YAQSPTYRFSGLTPGQEYVITVNVNSTSQRTLPEMPQNLESVGMQLFLELQWSPPIGVYDGYRITYTLDGTRTVIPDLPSQPPR
YTLRDLDPGTDYIVSVQALSGGLESEPVTRTLTTGVFDQLELSVTTVSNTSIALVWGIYEDRDNLPELTNYELSITPPDAISLI
RGLDVFRATFERLIPGQEYSIQLKLTTLDEPVSTVVQRTNPDPPAFLRSTRSNFTEIDLAFEAPLSRFDFFTLTYEKSDGSDLS
PNMRTIAGNEDSVTVEELQPGTSYDFSLTAKSGTPTEFTASQAVEDTFTTRAVPALTVVAAAITCDTIQIFWAPDPDDMLYRIG
IQPGPANDDFRRTTEYIFSGLNPATAYSISVTVVAFESNSQSEYLPLIQQTRPSGAGAITVIDKTTTTIDVNWLPAQGNFDSYR
VYVTSENGLPKAYPPISATEERRLSITELDANVLYTVSIETISFFGTQELTSCETSTLDVRTDALDANEIAVTAFTSTSITVIF
GKREDATGYSITLRNLDGNAPRDITVNLLPSESSRVTFDTLNPGVNYEITLDSQEDTTIEQRTKPVAPATVLTQNPTVTSFDVI
WTQPQGVYHNFLLVITNPDGTKRPEIALGPLDAVREVTELLPGNQYDIQVYATVGTGEDLIRSEPASAQGITNSLGALEVIYID
RTTDSLEVAWGASPRPNPGVYRVSFNTEGLTDTTPLITQDRNIRGTSLVAGQLYNVFVVAENYQQDSGILNVRTIPFVVRNLAL
TGEPTAISFSISWNQPASGMYDGYAVYIAGQDNVERLTSRLDDPDTTELLIEDLDPNQEYTVSVYAVAGFGQDSEVRSEVTEAT
VSTVSLDALDLETYSVTETTIGVAFGPALVAPSGYTLSLTSDGITIGLIIIPVAGSTKFVFTGLTSGTLYRAQVQVTGSSVTSD
PLDIRTIPRQPSSFMDTLVTADEISLSWLRPPSGNFDSYELSYTPENGAKVTLPDVATDETSLVLENLATATEYVILLRTQAGE
DTFSEPLELTVTTRSALLALQALPDPTSDVSLDWDREAGSIQRYMLIYDPNNGDPISPIETGIPGPVAISGLMPGSQYTFRLFS
VLTNNVQFLSSSVSHVTTPQPPGNVAVTEEMTSTITLSWSAPSQEQFFGYILEYEGTGFSTELGRTPATSVLPRHQESVTLKGL
TPGTSYSIKISAFVEYDDVTTRSNIVTKIGTTVSTSSLNIVAKFVNQTSFTIEWQPAVGASSYVTRVSEVTSGVEISSGTILNG
DYERTEHRLVPGRNYQIEVSATGTGQSDTVEQRTLPSAVSDVDVVPEAAALTLSWDPVFGDVDGYHASYQLKENPYQISDLFYV
SDPSNPTIRLTGLSPLSEYIITLTSFSGSGSDQEISSSTVEASTVNYSPAEIVIETVTSTSIALSWGASTSPTASSYVIEASDA
GNVVHSTSRALSQSRFIIISNLTPGTLYTIRITVTGSSERDTEIALTVPTAPRSLSTDSISQSIVTVSWSQGLGNVDEYDIYLR
QAGESETPAQIVPPSQLTETFENLEPDTYYSMRIVSVVRAQNLEQRSIPAVTNFTTQPRIINVVETGRNLRLSWANDFGNDILK
YQLIYIEDKEGATSNSEFVDPSDAPLVTLMNLMPGQTYTLILTAVDTLGNRAQIASTTYTLKPLRPTFTVNRRSPSAVSLLGNL
LGGILKFFRIRVTPQVTGAGAGTPINIDVLIPVEACPEIELTGLIPNRDYQVTLQAESGVASSDPQIITFTTPPLTQQGELSFG
EITPSGFTVYWTPFLGRTSYELSINNQAGLVVPASGDAVQSYVVDGLSSGTEYTVSLVDGGTGAIRTRPLAPGTPLATQVLDTS
IQLQWAQSSDGNVAGYEICYSPDDGSSPVYIAGRTTTQYTLEGLDSDTTYIISISALTGVPQVKSQKQTIFVTTIADTTIPVAS
NFAVTNTQAADHVIILEWDVPERASYDYFEVSYTPDVGYPSSPVRLPKAATSLSIMNALADKVYQFSLVSIRGDERTDPPLTTT
GQIAVNNLIVVIGERTTTSLEILWGASASGSVDEYQVSYVGSTPIVVDGSSDRRVVIPGLSPGTEYTVTVQAINAGAAVGDSME
TASTLHEINGLSAMVTDQASSMIHLTITPPFEDPMRYDGYNVRVRLGGVSSPVVQPVIPVAKSACPEIWIKKLEAATMYEIDIE
WYSGLDISEAFRVQTSTVAAAPLDITLDELETDSMKVSWQEGTGSFSNYLVTYSPVGSTQTPMLVAKGEPRVLDLSGLVPGTAY
TIMVQQQGQTPAQETHVQLTLPEAINIDSLSVTRSKNALDATWSAPSTGNYDGYRVCHYPRGTQNSPFTSGTTPSINLPGLNSL
TYYLVTVSSSEESTYSEPTTITQQTLIGDPGDINIALEDVDTNSVRITWVAINSITRYTVGGGTGASQTVTVNSPEEAEYTFTN
LVPGTSYTFFVTPLGTQRREREQYTRPDMPRNLLFNTITTNSISIGWEKPLTGGVDSYRVTYSPSSDGSPASPHTITDNGQITQ
TLTVTNLLPGTTYTFNVYALVAELVSEPVTRGSGTTIGMDIGAGYTSTQFTVVWNVPTSGDFNQFQVTYDPYDPTNAQESPRII
SLSSLNGAQEGSLLIYGLEPGQLYNVYVQTMLNGLQTDDGFGRISFRTPPLPVTGVVITQRTPYSLSLDWSDVISADIQYRITY
APLEGALFPNNEDSAQVLSSNQVSESQISARSLAPGKEYVFTISPVSGDPSSFEQVGEPETVVTYTQPLSPQNLVVSDVGVEEV
SLSWNLPAEVNFPIAYYEIDLVPNEPTFPIRINGTRDKWEIHDGLVSGGEYQAVMRSVINDVNGQVIYSEPLQSAFFLLEPTQP
QRLSTPYVTTTELRVEWTLGNSNFTTFQVSFSKDSATILTTDTTEQSLLLTNLTVATVYDISIRTVVEGPQTNMIKTSAAALIM
AVTTKSKPPQNLMQDEVTSDSITVSWGAPLNVVNMYHIIVKDEDGADVADATVPSSETSYTIENLNPYTKYIITVAARFNGLDS
DAIEIEQTTFQGKPSVVRDPVVAINSPNSVFLTWTPPDSPNGILTGFVVEITGEDLTSRKRREAGFTRTETYGVTVFSATIENL
PTGCSYTFSIKAANSADQSDAVIAGPLDLPHSAPPTPPADRVVRLVSHTGTTMSVSFDSSIFSDVYGPVTRYALLVAQSSPDTI
PSTPSDPAINWQVGSGQPYQTTLLTYAPFTNGNTRGFYTVGANNSCTQDSVGYCNGPVVPLTDYRFALRAYGSDGKFTDSAWSP
LYNSGINTTWYAAAVVSIIIITLVLLLVMLLISRQCCGEARSSALYERGNGQHLPLVNAAYPSAHNQDSMHKTPLPEDAFIRMG
PIPVRENAYVSMRDDRPKAPLPATLPRSAPPLTNPVQMRKFPDHVNRMSAGNNTGFVEEYNSLSNVGRDKSTDASRLAVNATKN
RYRNILPFDETAVHLQDLEEGQTSDYINANYIQGLHSMKEYIASQGPVPDSVNDFWEMVFENKSTTIAMITGLVEGGKTKCEHY
WPDDDTPVNYGSVTVTMTHTQEMEQWTVRSFLLEKGMQQFETHQYAFKGWVDHDVPNNARPMIEFIRTIDVAHDANLGPITVHC
SAGIGRTGVFIALHKLIKQVETSKPNDYIDVCGTVARMREQRFNMVQTVKQYMFIHQCLLLLMR

SeaUrchin4 SPU_016937.1 XP_783483. LOC578203, partial [Strongylocentrotus purpuratus]

MVVLLPYLPLVLIAGARILSVVNTANALTFLWDEQPNPETFTGYLITSASINEVIPKQSEETSYQKSISNLTPGQLYEVSLFLQ
PITTFPAPLDQLQVRTAPSPPESLRVVDETITTLRIEWDSPASGLYRGFEVNISPPTALQELPRIVGSDIRGFTFVGLMHNTEY
TVSVTTIMGEEGLEERSTTAEKTVMTARGDGDEVYLLSVSSTDAQIGWTIPPPTTVYLDGKQITVLSQPHTIPSLTPGRQYTFT
VEDVDLAFTTVPERPDGLELESVGPYSLDWKVDTSLNDLNYERLEVSYSPESSQTDSPFDISRCTDGTFTLEGLQPDTEYSVSV
TAISGDTRSDVLTRYGFTTCALEGQLHVVSLSSDEIALAWGPVNNIQGYAVRLFNAIGINMRTLSLSANSMSATLDSLSPASNY
SVQLIVMDTDGSRQTSVLQFVTDPAPPGPITLQSSTQTRLSFTWTPATSNFDSYELSISGGDMPEPRPVGVVYPNEGNEFEVGG
LLPDTTYNVRIYSVFFESRSIASEASFNTDEQILEFNVGSNQNLTVSWQVQEGAESYRLIYTTTNRNTNSIARPATSTSITIDN
LLFGAIYNFRLATIAADGTEMEVANADFVQSPLTPSTNISDIKATTALLTFSLREGIFDDYVVMLSSPTDSCGARQPVFTLSRE
ACPELLLLDLYPNTMYTAQVFARTGERGLSLPSETTLQNFTTATVVNNEVEISKVGTNSLEIIWKTDNVDEYLLHYSGPDGPLM
SDIVPYIEINYYTFGELTPGTQYSISVISLINRERQGDALEVMQYTKPDPISNLVLTPQENTINLTWTSPSTMYDGFLICWYSA
TTEDNKAWLDGDATTYPWVISTPNEEYVVSVATFVGDREGDYVLSNKVTDFITLTETSPLILKDIATDTISVSWVTIPGVVDYT
LSYSPPDGNEQATQDIDESSAIAEGLNNIYYVNFTGLTPGREYTFTVVWPLAFNYSTDAIRTNPLPIYDPSVTNTTSCNLLYQW
CLPDSDFDNISITIVPDVSMRTLSRGNGTGGMCDYQTVYLDGLEPATSYNVSVEVMSGDKSSEVVMVYSTTEPLPVNNVTLDDS
TIFTLTISWENPIDGSFNMFLVEYAPVRSPDSVMALDPINMGTNTAIIPNLQPGTLYNIMVYALAGEERSDATEITAMTRQQNS
EELRLQTRTSTSISVSWGNTTRPEVTGFVLRITFQEGDAIDSIKNVSLGDVLGVEFSDLIPGTNYTVYLLVEVEGQLDTAVHSL
DVTTLPGMPGNISCDPTKTTQSLLEIEWGSTQEGNFDGYAISTQTGDGEEIPLESVEKDEQLQATADYLMDNTIYTFIVRSTLG
DLRGEPTTMTCRTDPQNLMVSTSTSPSGGQEVFLDWQDYGASSYKVEYTPQEAPLPVVQVIIVLRRPTSFTIQITLPRRGRYRF
LRLRVRRRSQGLDAGPDGDEFLIPTDACPEYTFNNLLPDTDYAVDVNTVAEDGRTESSPSTMDVTTPRMEEGDISFNEVTTTSI
SFAWGMVSADTNGPFDFYLLNLKNELSIDVQNVQVNTSETREHTFIGLMPGELYRVELIVNSNRQRSAPMYTRPDKPTDIMLSD
LTTTSLTIDWKAPLTSYQGFRLCWAYGETNSLELGPAVMERTLSDLEAGAEYLVSLAAVVGTGANQITSEKFTTIVTLFPPAEL
HVNITSFNTTAVSIVWGTDIPLLDVSSYVVSYWETANNGSVQDISVDDPSQTDYTIDGLVPGTRYSFMVDALGTVQAVTSIGAT
QYTNPETVAEIRVIPSITCSVRLEWDRPEGNLDSYIFHYNVTRGTIVGAPLQLDVAINDTAETLTYLLEGLEKDSSSDFVSLEV
GVTTVAGAGEDQVESLQQVVKDLDVNVVTITILGRDQNSIQAEVNSSVSNVESITTGLYNAEMVTSSTTRNCSTMDCESFTVDF
DTLNSGTLYTLYAAVVQSSGREVVPLLAKAATIPESAVDLQFTSIGRDYVVLTWDNPAGMIDSYNISYYPVNDITKLMFEVVQA
AAESNVLRVDDLNEGMNYSFTVVSLLEVEADLQEMGAPVEVFAVVGVLGSLNITAFDETTISIEWEQVDVEEYILSYDALEGGS
GNVTVQSTDPNSYTFTGLTPGTQYTIGVQPGIYAALEKTQSTVPNPPTNLRTTMVAAQSITVSWDPPSEGGSDTYNITYNVTGG
QPPSPHTVSTSETSVTFSNLTPLTEYFFTIVNIWKSLPSRSELTRATTSAQTLSLAVTPEDNTEFTVSWEDLDTEAFEQYCVMY
EPYEAQENRQQESFPVARNIRSARFYGLDPGQEYTVYVTSCSSVSDPTSHANERISFRLDPAAIAEIRIMDGSLGPRAVMLEWD
QPEGIRGLYEVTLDPADEGSVDVDGDELPVNITGVMTSIKILNLAAGKGYTVGITAISGDKRSENTTFLFYTLPDPPQNLVISE
IGEDTATLSWADPAEMNFDILEVVMTTMPATKTTILKLNTNTVELTNLVPGQTYTVQLASVIRPDDALNQPEVIGEPQVSSPFL
MKPASPSDISVSNNELTRVTVTWQPSEGVVARYSYTYGLPGSTVTASETTTTTDTSIQLSNLTPSTRYEFRLWAESDQTEGSET
SSSDVVTVNIVTTVFFNVPSDPVSALPVTTLPSMPSAVGGLTITKTGIVDAEVSWSSPLLPNGVIANYSLTIHISHDNGTLSPY
GDTVTISAVAEQTEYSQDVIGLAAGQYYVFSVYATNQVASSNQRSTTRTLFAETAPPSPPRGATPSLVSRGSTITITFFNLFDT
AFGRIVRFAIIVQERVDGGTVVITAKRQTTSSLTWAEARRTRPVPSYQTTPDDYNPFADGAGVTTFRVGSQSCDPDDLTAYCNG
ALYPGTSYRFAIRAYGVDGKFVDTEFSSPFRTDPDRGIFVIPAIVLAIMVVIILIIFMAMGFGCCGKPDDGMYSDDSNGYGNDY
SNGNGYSNGNGNGIMKRALINDGVNTYAYQQPQIMEHSFTNIPDYSTIPPNRGTTAQVHQPSTTHTRPTKKNMFAQHVAKMSAR
NNAGFSDEYNNLPAMEKTRAVTAARQARNDDKNRYRNILPYDYTRVQLKGVAGTDYINASYIKDDHGQKKYIATQGPLPNTIEH
FWEMVWENQTSTIVMMTALVEGGKTKCEHYWPAGEEPQLHGNVTVTLVGSNQTDNFIERTLLLEKEGTERTVTHYQYLAWTDHG
VPESTAPLVGLLRQVKVTNQEDGAATGPIVVHCSAGVGRTGTFIAADMLMDAIQRSTATDYIDVAGTIAKIREQRALLVQTLYQ
YIYLHRTVLSLIEEQ.

acornWorm gi|291235644|ref|XP_002737754.1| [Saccoglossus kowalevskii]

MGTQDDTLTGRVLIIIQVFIFIGFSNAGYKCTDDCAYSHDNLCDDGGVDSYSNYCYLGSDCDDCGPREVPDTTTVAPPTTPTTP
TTPSSTTRTTAMHAMQTTFVHTTTAASEAEHAHTTLNTSTDASKTTDLQTEGHETSGAESTHTPEIVTDVIHSSRTEYEVMSMM
TTNENEENNVTMYNIRTEEQETEVVDSSTLVGTEIVITGFTPTEEQETEVVDSSTLVGTEIVTTDFTPTEEQETEVVDSSILVG
TEIVTTDFTPTEEQETEVVDSSTVVGTERVTTDFTPTEEQETEAVGSSTLVGTEIVIISFTPTEEQETEVVDSSKLVGTEIVTT
DFTPTEEQETECVDSSTLVSTEIATNGFKPTEEQETEVVDSSTLIGTETMTTDFTPTEEQETEVIDSSTVVGTEIVIISFTPTE
EQETEVVDKTLTTDFTPTEEQETEVVDSSTVVGTEIVTTDFTPTEELETEVVESSTLVGTEIVTTDFTPTEEQETEVVDSSTLV
GTETLTTGFTPTEERETEVVGSSTLVGTEIVTTDFTPTEKQETEFVGSSTLVGTETMTTDFTPTEEQETESVGSSVVVGTDVGS
TGGAQTEGPETEVVDISTVVHTEMVTTGGTPTEEQETEVVGSSTLVGTEIVTTGFAPTEEQETEVVDSSTVVGTEIVTTDFTPT
EELETGVVDSSTVVGTEILTTDFTPTEKQETEFVGSSTLVGTETMTTDFTPTEEQETEFVGSSTLAGTEVTTTGFTPTEEQETE
SVGSSVVVGTDVGSTGGAQTEGPETEVVDISTVVHTEMVTTGGTPTEEQETEVVGSSTVMGTGVMTTGVTQTEKQETEIVGSSK
VDGTEVVTSGLMPTVEDGTGVIDSSRFASTVKVATRSTLHSTTMVRPTEEDTESTTVVLSTLPTILAVYVTSTEVQMADDSYLR
EWSDSVALTFDLKLSTNESLQSVSLNNDWKLIIYIRDVSSTNETVNERLRPYEVEVDNFQISGSDLVFNDIIVNMNLSGLLCSE
MTYICIELIAYDGNGTDLVFIREPVDCVSILCRGVEIAPFVDVVTGSKLREGMDDQSFQFNLILYPDPEAGSIEGEYLWTLTSF
FSQSNDGYGNTDAGTVHSLTTDQSGMSLYSGIPASWTGFRSSAINITDKLCSDVSKYFCVNISKSSLSNPDFTFGNPVMTCTPT
PCTGVDVVDTRLSIVSGIVVKEHESSQYFNVFVTLLSSPDFGDIEGASLWNVTMYVNTQGNANVGDVSALAVADIPRDMRDLSL
LAGSNAILEGVEAFMDLAELNCRQIRYLCVDVSRDQTSVPAFTLTGKLQTCAEITCRGVEITDTELIVKSGTSIVERSGNHSVT
FDVLLSSNPAAASISGDRLWEMYVYLSDVEFDDTHSFAATYPVLSSVEDTDLTAGVTLALLGIEAFLDIDSLTCDQMRYICVTV
FKGRYSSPEYTLTGVPDQNILTACQPVTCTGVIISTDLQLSDDFYLLEGVDNQHISYTMTLFASSAGAGVSGMNLWDIRTQLRS
IEDAIVDTSNGTIQEVNTTLNAGGVLLMSNVKSVLSLEGVLCHQFFNICAILNKNSNANTEYTIEYDRSTLVSCRSVSCKGVEI
IEVAPTIMNGDIVAEADDAHAVSINVGMTSSIYGTNRTGAGSWALEVFVTSDDVRMPKLVNLYATLTAAQQDTELIGGIPSVLS

NIEVELDVSALQCSEFTQLCVVLHKGDNAEPDFTLGVLQDENALPGCAHLSCQRVAFNISAINVTTSSFTIRWDEAVGDFDGYY
IDVSPALTGLSSSFITERDEPREASFFDLESGQEYVISVGIAKNGFPKSTGVTQYTQRTFPEKVDSLRVSMVTETEIHVVWDRP
DGVVSTYVISIQPSEGTATLPITLPSDSTTASFSGLSPMSQYVITIVTYAGSLQSKEVPVKVITGTIPPVLEVTSFTMDTITVA
TYVDNRLSHGMLHLSYFPTDAEPIPIELDLNKTSYVITDLVPGRLYTLEVKIVADIDGGRITSNSDRKYQQTEPSPPGSIVVSR
ITEFGAIVNWEPSSGDFNFYLVTYSSASVNTALPIRVNHGQTPQLSLSGLSPATVYTVHVSTRSGELTSQSVSTTFNTLLAPPG
RLSVNGVTSSSVQISWLPAISGLAVEYYVTYAPFVEGATPASPFTLNGTSVNITNLYPFTIYTVTVQTRIDDMYSRERGITATT
YPDLPSSVKGFNVTVESPYQVTLTIQQPSFANGILESYIVNVVGYKEGFPPHILDFEIPPDETDDITEYLLEDVIAGYTYTFTV
KPQNSLGIGPLRTAGQVIMPIYPPPKPTASRITANVRFVETTAYTLRIHLSNNLFDDRFGIIVAYLVIIAEDGEYSPLPEVLPS
YDEVIDSSPWPPYQTSQPFNPFETLSIGRRRRNTLQTVNYIIGAKENCDVSLVYCNGALRPVTDYRYVIRGYNELGNYTDTDWS
LPQKTDLDPFWILYGVIAGLILIGIIILICICYCCCCRRRRSSTQDSDKLSEPPSYTPYACENGVFFNGNEPTLEKTEKLYTLS
LRRKRKSRPVPLKGFRDYVNELDEDDRKGFEEEYDDLRKLVVRQPTSVGQRMEHRSKNRYTNIVPYDNNRVILSGSGNSYINAS
YISGSKGSRSYIATQGPLESTCGDFWKMIWQQRVTTVVMMTQCNELGKSKCHHYWPRDTQTQISHSDLTIRLTSETRLPDWIIR
DFSIESNGEIRALRQFHFTSWPITGTPYDADPLIRFIEAIRIQVLPNSGPILVHCSAGVGRTGVFIALYHLLEYFYTMIQVDIF
GKVIKMRKQRPFMVQTQGQYEFLYYAIQQHIKEKPLGTNWSMRKTMDSNWTITYGGTVDPDDSGPGFFDPYDPEEV

acorworm1. XP_006823994 curated profiling| partial [Saccoglossus kowalevskii]

MSLKHAFCFPANVNVIDYGTDYITVNWTHSGEHVDEFVITAIATENEAANGTETVNITRGTVDGLTPGAEYTITVVAVSQDDVE
SLPSDEVIQRTIPEPPYNVTVESYNTDSIHLRWEPPLSNVFTHYNVTYVADSDPGMTVTVNKSTTESSLTGLISGETYNITIKT
ISGDESSGASDAVVQTTEPNSVVLARDETQITTDTIAVTWSSYSGDIVDTYTVQCSSNGTAEEATVDYVNGTDTYSAKCTGYET
AGKLENITVTAVSGTSGPSQSLSEPSMIEIHTKPNSVVLARNDSQITNDTIAVIWNSNAGDEVSYYTVECSDGGTPGQAMIIYD
NTVTYSAKCTGYGTAGKLENITVTAVSGTSKPSQSESEPSKLAIHTKPKSVTLARDETQITNDTIAVTWSSYSGDIVNTYTVQC
SSGGTAEEATVDYVNDTVTYSAKCTGYGTAGKLENITVTAVSGDINTTGSESEPSTMDIHTKPNSVTLARDDSQITTDTIAVTW
SSNSGDIVDTYTVQCSSGGTAEEATVDYVNDTVTYSAKCTGYGTAGKLESITVTAVSGDLNTTGSKSEPSTMEINTIPNQADPV
RNDNLVTNTTIGVKWTDPGGNINHYEIGCPDGGTPSANVTHTPDKEMTAQCTALATPGGLYSITVTSVSGNSKSDPAVTSMNTK
PNKVRLERDDDNITNDTIAVTWNSNSGDEVTYYTVECSSGGTAEDSRVDYVNGTDTYSAKCTGYETAGKLETITVTAVSGDLST
TGSESEPSMMEIHTESNAITLTKDSEYVTTTSIAVTWTNPGGAVAYYTVECSEGGTAENGSIPYIDNDQHEYSGKCINYNNPGN
LETINVTSINADNHRGETSSMEINTEPLPVTDLTKVDATTAFIEFSWIEPESGTWSGYDVGYLSEDGTQQFSEYLSDDVHTITS
HTFNNLTAGVLYDITVITVSGEEMSTNETEPMRTIPNAVTDLVLTTPNQTNPEIVIMWSPVEDEVSHYRVKCNDSDDVTTEPDD
QIIPHENVTSLTTECSDLFPGRLYNVSVSSISGYEGVDETESEPIVKAKRTHPKPVTSLSLTSPDNSVDTIHVSWTAPEDSDVD
GYRVVCTETDSGDKIDDVEVTVTNYDFSGLTAGRLYTIDVYSYSGSDNDEKLYSVNTTDVKRTYPKPVTSLSLTSPDNSVDTIH
VSWTAPEDSDVDGYRVVCTETDSGDKIDDVEVTVTNYDFSGLTAGRLYTIDVYSYSGSDNDEKLYSVNTTDVKRTYPNSVTITS
TSTTTDSLNVTWSLPDGNWTGYRIEYVPQDNLANAVLRREDISDPEQKIVHHTIYDLYAGTLYNVTIFTVSSGIDQLESLPDTT
EDRTVPATAYNVTEVESSKTSYSLEFSWKINTTSLHDYYEVYYRGQLDTSFRNTTSDSENVSLVDLRAGDTYNVTIITISGKKR
SAPSEPAYGTTTPLSPGAVSVIDVTETEIKIQWAQACENCTYDYYVHHEPEDGEVDVDSSSLHAKLYDLNHGTLYNISVVSISN
DVPSEPTYTLARTLPDTPENITIETGMRSLNVTWDEPEGEYAGYNISIEPSTNRAGKDIIVDVPDKGIEECIITECDPYTNYKI
TIIVHSDEPNQTFSEPAVSHAMTETAPPPPPRNNIVPSSSESDIEVSQTTITVLFTDDYFNDSNGPLLNFTVIVTEDGTEEVDK
HLNGEIPRVYCKDCENVPGIEEGEVKTWAEVQNTPARYQIDYPYDYPDLSRRKRDTTGIEITIGSDDSCSTKDEGFCNGPLKDG
TSYRYHFRAYTDVGYSDTVLSGPIRTSTNMSWLWLLLGVLLGVLIAVAIILAIIYYKRRYYEETTTRTVEGTANPEFIGLRDEN
GKPPIAQKPKTQKHSKPVKLAEYHGYFQIMSADMEFRFSEEYDELRPVGREQSWDAAELPENRAKNRYTNILPYDRTRVKLSQV
EDDEATDYLNANWMPGFNSPREFIAVQGTLPGTKDDFWRMIWEYNVSTIVMVTQCQERGRVKCERYWPTDDNPVYYGDVLVTVT
HENELTDWVIREFTVENGKSLRRIRHFNFTAWPDHGVPEETGSLIKFVRSVRAQIQNDGTPTVVHCSAGVGRTGTFIALDRLIQ
HIKEYDYVDIFGIACEMRMHRVYMIQTESQYIFIHLCVDDLLKEREQPDEPDEPDSTPDTTEHIYGNVPSVTVMRNGPDVVTAN
LGDAYKRNSVIAQA

ciona ENSCSAVT00000019606 plus GENSACAN and profiling [Ciona Savigny]

MIKQLVILLFFRFLIHFRTLNKSIAESSTFSLEGQLQLDSLARTTTLLNLEPHRHYVLRIITISNHTTPRGWVTSESESTSLSF
STRKLDPDIKYRLPFITISPALAPPRSLHVNHTSITEESFEIAWKSAVGLVEKYRIVLKTKSADETKILVANNFATSYFVDGLS
PGESYEIAMISLKGIDQSSAVFVSQTTRAISDMAPPVNFRMTSRGETEITLGWDPAPGVQGVYMVRCEGFQEPCPAEVDIIRPD
EKRELRVGRLKPGARYNFTIQTQNGQDISKAKRLSLITVPRTPLSLDTVSRSDTTISLRWDQPTGILDGYKISYTSRGGQLEIL
FVQPGIFEKTLNNLRPGTYYEISLVSTSSEIESEPIILTEITELREIENLSIARLGETWAFISWVPGEGGYSGFDYIVQVATVL
DKDRSVARNITIQTRPLPPNDVRMDNLTKSSVHVWWREPETFDQFLLVYKDDFNTKNLMTQQTSVDINDLTPGSSYVIDICVIF
GSVKSYNVTNEITTYIIAEPGTPTALVLKPVSTTELLTEWEIPLNPNGIIRRYIIRFKQNYPHPSTNYTLIETNQTRYLLGNLE
PGAEYVALFAAVNSAGVGNFTESYFFKMPESKPGPVSDLHVSSVNAISATVAWQPPEQPNGEIIRYVLNISTDHLPVLSVDLLK
RSNSDKNLCLFDHKSKSDKTALVSVTSSLQNNSVTEALNDEIYFDPLCPVFVNVTSLLPFTLYRFRVTAWTSSGEGDSRIREEM
TLEDVPDDPPRHVRLTSATHTSLNVTWFPPNQPNGIVTYTIAVLQSDKMFQSNLTYYDINELRPFTKYGVMVRAETSVGVSPWS
EILFADTQQGRPGSVQNLTAQVETSRSVTLQWNPPINPNGVITGYWIFAKVKNVTQLTLWLEGVSLNSGVNLRTLQDVPSSPPI
NLSYYNVSSTAVNITWDEPLEANGVIIKYVVYYMTKDTFLDVTVTERFALIKDLKIFSPYEVIRVRPYTLLGDGPASGTLLIHT
DEDFPASPVRNVTSKNITSSVVELSWTPPLIINGRARSYTVYTVVLLNSYNAFNSYSNKTSAFLTNLRVFTAYKVQVACQTKKG
LGNILSKTIFIQTEEGQPATPPFNVSYQNLTSTKVRLTWRRPLVPNGIIQFYEISLTSKNNKTIRATTENDVTAVTVDHLTAYS
EYTATVRANTKFGDGSQQSHALTIHTLEDVPGSPVTNVTYVNLTSSSIGLEWSLPKEPNGKILKYSIRYSMLEGNMNQYKAVND
TFVELFDLNKYTRYNVSVSAHTVAGEGPPAYISLHTDEDEPESAPYDIIFQQYNSTTIALTWRPPVKPNGIIVNYTVYYSNEDK
VMTKTTTKPRTVLRNLEKFTEYEVYLTASTKLGNGGKISAIETFQTLEDAPADPPRDVVVKALSSTSISVGWSTPATPNGQIQF
YTVFYTDKISAVHATNVTTVISMNRSTSSSKILLQITNGTNAVIKDLNKYSDYLVWVSGSTALGDGNQLSDAKKVRTMEDVPDN

GVSDLSALAINATTIKVTWQPGIPLTGPTFFHIQYVLNNTPVHDLNFISFQPHTEYVIKVYPRVVGVYDAGPVSSIVVTTMESA
PSSPVQNLHVVYVTETIVKLKWKPPSEANGVITSYTITVTKQNYNKTKTGNETETVIEDLRANSHYNVSVTSSTKIGEGPSTIL
LFRTKEGVPQDAVTDIQLINLTSDSALIKWLAPRQPNGVITHYTVHYGRNSTIQTNVTEATLRDLHPFQLYWIEVYPWTSAGIG
VKPSEKMAIKTKESAPTAPTSLNCSVASNFSVKISWDLPMRTNGIIRGYNLEYRSMDKNKVKFVQPSQTKQIISNLKPFANYLI
KLRAINLAGKGQWTHCNVTTLEGYADAPPQLQLKNLSDRSITIEWSRPLQWNGRFHGYLITYKPPDSCPNPANDTQVCSFTSNL
TSVTLEALSKYRAYNISVGSITGRGVGTMRDIFVSTLVGVPDSPVRDVHVTVVSSTSINVTWNLPSSYAGPTTYKLYSELVPLY
DYFDHTAQYLRVHNHENNSPFSVGLDEDTTYGVIVTTMTTVGELSSIPVSIDTKEDAPSDFPESVGVATVLNNSSSVHVLFKEP
LDPNGKLINYTIQHRRLEDSKLQSIVVSVDQLTAPTTEHEPYSVSVSGLLGGRYYQFRVRAATAVGNGPWSLWTNKVLLPTTAP
PVPDTWPEVMNKIDDTSTMASVTSSTITVKKPCMFSNENGPIASLSVIIAEDGGNIEAEPTYWSQAFPLQPSPPYKVLVSENPT
DYCNTRKRRSINEKGFVIGTSDCPSELNTHCNGPLKSSTDYRVKFRAETSNGLKTDTEFSEIIRTSPSFLATHMTLLIGIGAAI
GLFTILVVISAVFLRLVMQYSIQYGFLNNVFPIMETSIHTNMAFIGEASMDDKVQEESVHGNEFSRPVFRDEFPNHVSSLSQNK
NKGFSLEFDDIRGIPYAGTTAIAENSCNKTKNRTTKLVPFDHCRVKIEGIPAIQGSNYINASYIPGLDSPEQYIATQTPLDHTK
KDFWRMLWETGSTNIVMLCGSVNAGKKRCDEFWPKKQTEYFGNLAVQMKEEIRHDEWIIRQFIVTMRDKVRHVTQHHFIKWPQL
EQAENSLPLVRFIKNYRLLRDRSINPTIVMCSNGSGRCGVFIGLLRILDNNGRDVDVFGTVAALRKYRPYMVQTLSEYIYLHQC
VLKFIDD

Ciona2 XP_018672020.1 XP_002123247.2 phosphatidylinositol phosphatase PTPRQ, partial [Ciona intestinalis]

MEETPGPVNNLQVSSYNATTVTISWQPPLEPNGEIIYYKLTVYKQNVLATIINFEKKQNENQFNCLLDNAPRDVLSSFMQLLLP
NSLLQHNPSTTLTKESGDVTIQALSEIITSQTDNNSTKKGKQRTLRSIVTETNEYFEPLCPVATIVTSLKPFTNYRFNVVGWTA
VGEGIGVDVSHTMPQEIPEGPPLNVRVDLATDSTIRVMWSSPAKPNGIVTYTINIELGNWVYQSNTTYYDVTNLKPFSTYAIKV
KAITKVGEGPWSNWIIANTLEGSPESVNSFNVTSKTSRNITLVWEPPLTPNGVLLGYYLIVEENNHIVRSYWLAENNGATREIP
SQSDDVIQFDDDILGGKLVDYSMKIKTKTISDLTPHTAYAFNIFAATVGGNGSQLTLYDVTLEDKPDSPPYNLSYSNITSTSVN
ISWMEPLKANGVITKYTVYITKDKLQETSVTERYAVISGLNIYTTYEVYVRATTKVGDGPSSSKMQIHTDEDVPGSPVLDVNFQ
NISSSVVKVTWSPPLVTNGVILGYTIFYSNFTTYNTTSKTTSVYLEKLKMFANYTVRIASQTSKGLSPMLSGAITIQTMEGQPS
TPPFNLSYTNTTSSVIQLTWLKPLVPNGIIQFYEITLQTQNNNTIRKTTQSSETSKTIKGLKPFSLYLANVRANTKYGDGGQAS
ATYLIYTMEDAPGSQVMNVSYVNLTSTSVNVSWLQPAEPNGRITKYKIESKLFKTKETNKTFITLTNLKKYTEYIISISAYTMA
GEGPREVVKICTDEDEPSSPPDGIFFHQHNSTSISLTWNPPLTPNGIITLYSVHYRHGNKSLIRTTATPGITLNNLKKFTSYDV
YIRASTKFGDGNQTSKIKSFKTLHDAPADPPHDVRVAAISSTSINVTWSPPTTPNGLIQFYTVYYQHNPSTVQTKNVTKGMQVV
IGNLRKFTNYLVWVTSSTALGDGNQMSDAIKVQTLEDVPGPTSNLNCTAFNKSTMMVSWDPPMEANGILLSYTLKINEGETKFF
NTNTNHYILTDLTPFTQYTASISAQTTVGEGPISVCNTTTLQSVPDKPSQFKLVSYNDTSSLVSWKKPTNEYGVITNYILEHIH
NNVTTTQNITPINNKINILTTNLLKHSEYKTRCAAFTIKGQGNWTRFITFTTLPGVPEQKVQELSPRVVNSTAIRVTWLPGQPL
TGVTYFMVNVSTKTSVIVAKNESLNNVEIFNLQPYTEYIISVTPHVYGVDTPGPTSLVTVTTDEAKPSSPVRNISIITITQTTV
TITFLPPTKSNGVITSYSIMTSLDHKQLVNGSHTKIKLENLKPYKVYNVTVMPYTKMGKGPKAILTFQTLEGVPEDSPQKVQIT
NITDQSASLHWEPPTLPNGIIRTYTISYQSNESQRNFNSSKTQVQLTNLAPFTNYSLQLYAWTTRGMGKLPSRVLYFTTHESEP
SAPTNLECKIYDNYKILVTWDLPLLSNGRVIAYKLSHSVLNDTQTYFVKPPQINQTLQHLQPFTEYFISVNAKTSAGWGPSATC
NITTGEGYATAPQDLNINKTSDVTITIQWNKPNNVNGRLNGYVIMYIPFDACQPNSGILNKTRCILSTNKTWTTLIGMKQHTEY
KISVFATTGRGLGAPSNISVRTLVGVPEHSVENLQAIVTSSTTVNVTWESPESFAGPTTYKVEAFHSTTMQRVTSPLLTSSTHS
YVMSGLDEDSIYGVTVSTMTSVGQLSSKPVVIMTYEDVPSDFPRDVNVAIVMGNSSSVRVLFKPPRDPNGVLTNYTIQFKRLEN
SESRNAIIPVSDLVEPTSNNVPYYVTINGLLGGRLYKFRVRAATAVGAGPWSLWTTDVQLPITAPPVPALLPEVIIQQDKAIVT
SSSVIVKKPCVFSDDNGPLKSLSVIVAQEGATLDAEPTYWAKAYSEEPSPPYKVIVTEEPQSYCNSRNKRSTQTNNGFVIGTSD
CSRLSTSQCNGPLKSNTEYRFKYRAEANNGLMTDTEYSEVFRTNPSFIEAHMTLLVSIGAALGIFIILLTISVALLRRRRRNQI
PNGNESLKTMGPIETMGPIIEEKSKVPVMESSVHSNVAFVMDDSRDMKDTARSPSINEFSRPVAVTDFSLLVEKLSQNKNKGFS
SEFDDIRGIPYAGTTSVAEKACNKTKNRTAKLVPFDHCRVKIEGIPAVEGSNYINASYVPGPHSPEQYVATQTPLDHTKKDFWR
LIWETGSNTIVMLCNVMEGGKKRCSEYWPKKQTEYFGNLAIQMTKQDVCKDWIVRHFSVTMRDKVRHVTQFQFMNWSVVEQGNP
LPLVRFIKSYRTTRHNEYKPTIVMCSNGSGRSGVFIGLDKIVDTMTSYIDVFGTVASLRKYRPFMVQTLTEYIVMHQCIVNFID
DVTIEQPTGDGLEETIF

CionaNoCat.1 Ciona XP_018669650.1 |XP_002120279.2| ENSCINT00000036281 (**ESTs**:BW474929.1| FF941901.1| FF962764.1| FF924695.1| BW485618.1| BW182207.1) PREDICTED: phosphotidylinositol phosphatase PTPRQ-like NO catalitic domain [Ciona intestinalis]

MEQIAPKTIQVFKLYKLLFNLILSLRFPQVFFNLILSLRLPLFQTYIFVAVPTTAVTSLQVVPSARSAAVTFDAPVPVTGVFVY
TVQYYVTSAGVGTAQSVTLNTNSGTVPALTPNSAYTFVVTPRTGATGAGPTATATNTTLEDTPGMVSSLVVSSSRFDQIDVTWG
APTMPNGVITQYQITWSPGNGMMNFSSTTTTHSITPLTSNLDYTVTVYAYTAIGRGVSNSAVQRTLESVPIDPVTSVVIATAAT
SVNVSFNQPATVTGMLSYEVKYYVTSAGPSTAVEQNFTSTADTITGLMPNTAYTVIVTPQTTAGKGGAATVVTQTFMTDEAAAA
AVASVTEGTKTADSITVNWAPPAVNNGVITQYRVTWNSGAGNQVVTSGTSHTITGLSAFTDYTVTVYASTAVGEGPGVNQVIRT
NEAVPTDPVTSVNVVPQATSVGVSFNQPATVTGMLSYEVRYYVTSAGTSTAVDQNFTSTTDTITGLMPNTAYTVIVTPQTSAGK
GGAATATTQTFTTLEDVAAAVASVTEGTKTADSITVNWAPPAVNNGVITQYRVTWSPGAGSQVVTGATSYTITGLSAFTDYTVT
VYASTAVGEGPGVNQVIQTNEAVPAQIGAFVAVGRTNTIRVTITPPVPANGVLLRYEATLNQDQAECTRFGDTSCTQNRVQSVT
ANGNTPGVIVFGNLTSFRSYEITVRASTSAGFGPVSVILTSRTTSIAPQGVPTNVQITSLSDTSAIVTWDSAKPVPGNSTYIVT
LTAVSNSSDVRTITIIPSTVSLNNSVVFTNLQAGHGYSGSVVQSTSVGMSDPSTSATLYRAPTLIAGAAPPTAGPTTNALGVSP
TVAFQVENPCLRKAQLFSEIGGTINQIQLIVWQIQAPTPTTNANVWANVINQNPIVAYQAGTITCTGTASGRKKRALTANNDGY

VVGADSTCTTTTVAVCNGPLPSGRQFNVSYIGVNGGGQTSGMTAPGGPFSTSTPTGLEAGEIAAIVISCIVVLLLIISLIYYCV
KRRRASGMHTVSGRDNRSFDDTQKQRYQVEASETPSTTYSTTQKKGEI

CionaNoCat2 protein profiling [Ciona savigny]

SNLALVSNSFDTVQVTWAAPIMPNGVITAYRVEWTPGGGGVDLGSTATTHTISSLTAYTNYTVSVVASTAIGQGVRVSDTIATV
ESVPTDPVTSINLIAQATSVGVSFVPPATVTGVYTYQVRYYVTSAGSTTAMTQQFTTTSDSITGLLPHTAYTIEVRDTFMKFTD
LPTQVSPQTTAGIGPATTMTITTAEAAPVAVASVNSGGSTFNSLTVNWPAPMPPNGVITGYRVTWVPATGGGSAVVSTGTSHTI
SGLTAYTEYTVMVFASTAAGEGPGMSTTLRTAEAVPAKITTASLSSTATSIAVVITPPNPANGVLVAYMVSTRTYLVTIRANTS
VGYGAVSDPFTFRTLATTPQGSATNVNVVSLNATSALVNWMSELPVPGNSSYTITLTALTNPNINTVSWIFSIKTQTVVSRMVT
VNNTAVFTNLLTGHGYSVVITQSTSSGSGTASSPFLLYRAPTIISGAPGPILSPTQALFGASPTHAFQIENPCNRKSSLFSEIG
GVITAIEVIVWQTGAASSKWEAATPAIWADAINQNPIPPYVAGTIQCSTASGRKKRALTPNGSGYVIGAETCTAANRVTCNGPL
ISGRQYNVAYRGVNGLGTPSDMTASNGPFSTSTQRGLEAGEIAAIVISSIVVLLLIICLVYYCVKRRRSSGAHTVSGRDNRSFD
DSQKQRYKVEAATTDEAATTYSQQKYAKRL

ciona.1 genscan and curated by profiling gi|459174018 |ref|XP_002122888.2|] [Ciona
Intestinalis]

ENSCINT00000033578MPSLYVISSYEISLTLATSGTAVTKTYTVIAPALTYTVTGLTPGESYTATVQAVSSSVSGSVSTPS
LPQRTDVFENQVSSSVVSTVSNLVDVPAEELAVSGEADSLLMTLDTVGEKVELDENGVYEDVTSNVGMAALLPSNKKGRSGLGV
ESLSINSEPGFRSDQVYLIQGEIPNNVQSFITLPEEAGMLWVSCNMQIINACLFSVFGDSGNAKRIIFYAFPNDKLFLSPNNTY
YDVNSMVVLSATVEQTSVNNLQEPVIIQLVTNTHAVDAPSGITVDNIKSTEFTVSWTTPTDAISKMTVDIAGTASNENDAVDVV
SPHKVINTTAGAAIVGNTEYTITVYAVSSTDATDFKFATNQTTTIFSAPVLTSVTGTNSTIAVDLSWTYDNGGGANAVSEYLIK
WDGGGSTGSPTAGSGSTTATISSLSANTEYTFSITAVSATVRGDTSAPSSATTAGATTTSIDLTWTAPAVGGGKNNVLAYTIQW
TGGAGGSKETSGTTDTISSLSANTAYSFTVAAKSKAGTGEASTPLQAITLPSLPEQPTLTRSTTNPTTVIDVSWPAVTSGTETV
DYVVEWTPDEGPAANKAVTGTTTTIDSLVPGQQYNVTVRAHNSAGNSEPSPAARLRTNPPTPTGVSLSNPTNGQTSLKVDWQIT
KNNFVISSYEISLTPATSGAAVTKTYPANANSPFTYIVTGLTPGESYTATVQAFSSGVSGSISSASNSQTTVPPTPTGVTLSQS
AGDQTTSLKVDWVMPSLYVVSSYNISLIPESSGSAIVTQTFTPSPASTTTDTVTGLTPGESYTATVQAFSSGVSGNNSDASASL
RTVSAVPGTPNLYQPTDGSDKTTILYANWTVPTGVVDSYQLLVYLGSVGGTLVANQTVSTNFANITSLIPGKRYQATVRAFSAG
VAGGVSGNSNFAKTNPPTPTGVTLSQPNVNQTTSLKVDWVMIPTSFVISSYIISLISTTSGAVVNKTYTASAGSAPTYTVTGLT
PGESYTATVQAVSSAYDPSTQSDVASSTNTQRTDPPTPTGVQLFQPTVNQTTSLKVDWQITETDFVISSYDITLTPSTSGAAVT
KNYPANANSSFTYIVTGLTPGESYTATVQAVSSGVSSSVSTPSNPQRTDPPTPTNVTLSKPTVNQTTSLKVDWHITEAYFVVSS
YEISLKITGTTGAAVTKTYSANDASPFTYTVTGLTPGESYTATVQAVSSGVSSSVSTPSNPQRTDPGVPGIPTLSQPTDGSDKT
TILYANWTAPTGVVDSYKLLVYRGDVGGTVDANLIVTTNFANITSLIPGKRYQATVTAYSVGVASNVSGNSNYGKTNPPTPTNV
QLSQPTVDKTTSLVVNWQITPDSSVINYYNIATTAMSTGTITNFYYNLTEKSYTMPGLIPGESYAATVQAFSAKYPPATQSFVG
SSLINQRTAKQNPLQLIQYLQQHNLTYVQDGDASTKQTVTLENATTSYNITGLTSGASYTISVVTISNGVRSDEAKQQFSTITK
GVSNLMAQSQTTNSIYVTWNPPPMDVLFTNYFVEYSKMNIFLMPEDHKNENIYLRICITLLLISGYLSYGFYSLWVNVTFTQPK
NGANIYIATLTGVTINEQRNGSNSSSPVQVGGLSPGHTYSAVVSASASATILVEGVASSAIIHTIKPEPVTNFRSTEQTSYSIY
MSWDLPTLGIFQSQNVTYVPSDSSENAIVVSLGNETTSYNITGLTSGVKYTISVITISNNIESDPATDESMTGPPVATNISITA
SNTTCVHGTIIYVGRGIIHFYSLTWGFHGSKLVPYHPHITHFNLCGLQAGVEYDINVVSLAGNVSSTYNISTSAYTEPNSVLSV
MLSSVSITEISVQWTRNSGGVSGYEVIATNSNCVVEANITIAGNTTLSTTLSGLTPGTVFNVQVKALFSALKSFPVYQSAPTCE
LVNYTDPEGGDKSIQTNFYETSATLTVLPGYNYTVMVSVTAYGLNSVIQTMTKNTKFNVMIKSRINNVNGVIENSDTFTELYKT
NAGPPEEPNITQPVNTGSTSNMNTITIILPANTFNDKQGPIEAFGVYITKETQKKPGPQPDLSRNDSCTNLMTECVAVWYTPAG
VANTPSTSSRKKRSYDNPIGNNLITFVIGSGESTISPWKTLFVNLPLAVDTPYVVAVAAKTSNNVLVSTNWSQPIRTETPVPPA
PNVGLIVGLTVACVVIVVLLIVVAWFYRKRRKESKKDVSSENQIPLQQRKTKVIQLAEFLDLLKVMKADSDFKFSEEYEEFKTV
GRDQATVAALLPENRGKNRYTNILPYDATRVKLSAIDDEQGTDYINANFIPGNNNRQREYIATQGPLPGTKEDFWRMVWEQNSR
NIVMVTQTVERGKIKCDHYWPFDNEPITVADYTLQMTSESILPEWTIREFKITHGSDTRRIRQFHYTVWPDHGVPDTAETLVKF
IRYVRRTIDREAKHSGPTVVHCSAGVGRTGTFIAMDRLLQHLPDNNYVDIFGIVHQMRIHRVFMVQTESQYILIHQMVQDILNR
VYDEDDDDQEPVYENTTTISDPIYENAEFNGKQKNGGVVNPALISPSEDDQNKEDSEEEGEEESEEEGEEESEEEGNEENDQNE
SKLISGSGGPNHDPSVIIKQDAVEETSKERGSKATVV

Oikopleura1 gi|313238213|emb|CBY13306.1| [Oikopleura dioica]

MNIIANETFQLAPAVPRLRVAADPASSKKVRIDLSPALGETASTRVDSYNFWYFKENESSILRENTTIPAKIPNTFTIVKPGSL
YHYYAQAVVNGVASESTTVDFRVPPLQVSDLVVDAVTNSSITLSWSNPLVSDSDFEFVDILVPGIADKSSVPAGTANITLSGFA
GSTLANINVFTRTAGPFTAQSAPAIISVYTLPPRIDPESFNATQVHNKNAIALEWEPLSVFFTKYKIIVDYLLAFKPTRKEIIL
SPTTTEYLLKSDIASIISGNVYRVSIVALSKTNAGGDDEQEVSSYPTTKMVLILPEAPTFLSVNTKKSSSKSLTFQWSPSSGLY
DYFELKWQPDSDCPAKFEQVRNLSIQQAQKSMDPITNFVQYKIEELHPYCYYDFSVKAVLNSTSPIYAGFDEASLKTTEVVVTG
KTNAEPPPLLSQRTNTPVLNMSNVVIGKGTISFDVASLPFSDAFGPLLRFAVWVRKDTGDSELKPPSIPKTWTNENNREWIAFW
VKLKGDSSERTLVIGDGKESQNKDRYYKNAPLESGEKYRVSIQVENEAGASALYYSQPIEMNDAKKAIAIVIPIVVVLIVAVIV
LYYVLRRRQLSAPKDNAKNQELGLANGTAGPGGGHVNPVFTQDRPRIELNSVERKSKVIQLVDFKQVYDELRQDSSYKFAGEFF
ELKEVGKDQTTEASLLTANRGKNRYTNILAYDKTRVKLGATDDDPGSDYINANFITGNNSKREFIATQGPLPGTKEDFWRMCWE
QNTPIIVALCQVVERGRIKCDHYWPYDSDPEWVGSDLQLVMVTSFCETSRRIKQVSVSNQRKMTNIVLFQYHFTVWPDNGVPSS
LEPLVKFVRQVRFEMNRFLDRGPTIIHCSAGVGRTGTFVGYDRLLQDYENKSYIDIFGMVYEMRMSRCLMVQTEAQYICLHELV

YDLQIGKYNYDRDNGPSDNSGELKTEAQTIGAPQAGITNPALIDTSSSSSGSLTSDEEVILPKTPPIQLEPDENSNQANNNQLN
DAASL

DEUTEROSTOMES (VERTEBRATES)

PTPRQ.human gi|222537743|ref|NP_001138498.1| phosphatidylinositol phosphatase PTPRQ precursor [Homo sapiens]

MDFLIIFLLLFIGTSETQVDVSNVVPGTRYDITISSISTTYTSPVTRIVTTNVTKPGPPVFLAGERVGSAGILLSWNTPPNPNG
RIISYIVKYKEVCPWMQTVYTQVRSKPDSLEVLLTNLNPGTTYEIKVAAENSAGIGVFSDPFLFQTAESAPGKVVNLTVEAYNA
SAVKLIWYLPRQPNGKITSFKISVKHARSGIVVKDVSIRVEDILTGKLPECNENSESFLWSTASPSPTLGRVTPPSRTTHSSST
LTQNEISSVWKEPISFVVTHLRPYTTYLFEVSAATTEAGYIDSTIVRTPESVPEGPPQNCVTGNITGKSFSILWDPPTIVTGKF
SYRVELYGPSGRILDNSTKDLKFAFTNLTPFTMYDVYIAAETSAGTGPKSNISVFTPPDVPGAVFDLQLAEVESTQVRITWKKP
RQPNGIINQYRVKVLVPETGIILENTLLTGNNEYINDPMAPEIVNIVEPMVGLYEGSAEMSSDLHSLATFIYNSHPDKNFPARN
RAEDQTSPVVTTRNQYITDIAAEQLSYVIRRLVPFTEHMISVSAFTIMGEGPPTVLSVRTRQQVPSSIKIINYKNISSSSILLY
WDPPEYPNGKITHYTIYAMELDTNRAFQITTIDNSFLITGLKKYTKYKMRVAASTHVGESSLSEENDIFVRTSEDEPESSPQDV
EVIDVTADEIRLKWSPPEKPNGIIIAYEVLYKNIDTLYMKNTSTTDIILRNLRPHTLYNISVRSYTRFGHGNQVSSLLSVRTSE
TVPDSAPENITYKNISSGEIELSFLPPSSPNGIIQKYTIYLKRSNGNEERTINTTSLTQNIKVLKKYTQYIIEVSASTLKGEGV
RSAPISILTEEDAPDSPPQDFSVKQLSGVTVKLSWQPPLEPNGIILYYTVYVWNRSSLKTINVTETSLELSDLDYNVEYSAYVT
ASTRFGDGKTRSNIISFQTPEGAPSDPPKDVYYANLSSSSIILFWTPPSKPNGIIQYYSVYYRNTSGTFMQNFTLHEVTNDFDN
MTVSTIIDKLTIFSYYTFWLTASTSVGNGNKSSDIIEVYTDQDIPEGFVGNLTYESISSTAINVSWVPPAQPNGLVFYYVSLIL
QQTPRHVRPPLVTYERSIYFDNLEKYTDYILKITPSTEKGFSDTYTAQLYIKTEEDVPETSPIINTFKNLSSTSVLLSWDPPVK
PNGAIISYDLTLQGPNENYSFITSDNYIILEELSPFTLYSFFAAARTRKGLGPSSILFFYTDESVPLAPPQNLTLINCTSDFVW
LKWSPSPLPGGIVKVYSFKIHEHETDTIYYKNISGFKTEAKLVGLEPVSTYSIRVSAFTKVGNGNQFSNVVKFTTQESVPDVVQ
NMQCMATSWQSVLVKWDPPKKANGIITQYMVTVERNSTKVSPQDHMYTFIKLLANTSYVFKVRASTSAGEGDESTCHVSTLPET
VPSVPTNIAFSDVQSTSATLTWIRPDTILGYFQNYKITTQLRAQKCKEWESEECVEYQKIQYLYEAHLTEETVYGLKKFRWYRF
QVAASTNAGYGNASNWISTKTLPGPPDGPPENVHVVATSPFSISISWSEPAVITGPTCYLIDVKSVDNDEFNISFIKSNEENKT
IEIKDLEIFTRYSVVITAFTGNISAAYVEGKSSAEMIVTTLESAPKDPPNNMTFQKIPDEVTKFQLTFLPPSQPNGNIQVYQAL
VYREDDPTAVQIHNLSIIQKTNTFVIAMLEGLKGGHTYNISVYAVNSAGAGPKVPMRITMDIKAPARPKTKPTPIYDATGKLLV
TSTTITIRMPICYYSDDHGPIKNVQVLVTETGAQHDGNVTKWYDAYFNKARPYFTNEGFPNPPCTEGKTKFSGNEEIYIIGADN
ACMIPGNEDKICNGPLKPKKQYLFKFRATNIMGQFTDSDYSDPVKTLGEGLSERTVEIILSVTLCILSIILLGTAIFAFARIRQ
KQKEGGTYSPQDAEIIDTKLKLDQLITVADLELKDERLTRLLSYRKSIKPISKKSFLQHVEELCTNNNLKFQEEFSELPKFLQD
LSSTDADLPWNRAKNRFPNIKPYNNNRVKLIADASVPGSDYINASYISGYLCPNEFIATQGPLPGTVGDFWRMVWETRAKTLVM
LTQCFEKGRIRCHQYWPEDNKPVTVFGDIVITKLMEDVQIDWTIRDLKIERHGDCMTVRQCNFTAWPEHGVPENSAPLIHFVKL
VRASRAHDTTPMIVHCSAGVGRTGVFIALDHLTQHINDHDFVDIYGLVAELRSERMCMVQNLAQYIFLHQCILDLLSNKGSNQP
ICFVNYSALQKMDSLDAMEGDVELEWEETTM

PTPRQ.mouse gi|124487427|ref|NP_001074901.1| ENSMUST00000050702 phosphatidylinositol phosphatase PTPRQ precursor [Mus musculus]

MDFLFFFLFSLIGTSESQVDVSGSFDDTVYDITLSSISATTYSSPVSRTLATNVSKPGPPVFLAGERVGSAGILLSWNTPPNPN
GRIISYVVKYKEVCPWMQTAYTRVRAKPDSLEVLLTNLNPGTTYEIKVAAENSAGIGVFSDPFLFQTAESAPGKVVNLTVEALN
YSAVNLIWYLPRQPNGKITSFKISVKHARSGIVVKDVSIKVEDLLSGKLPECNENSDSFLWSTTSPSPTLSRATPPLRTTHLSN
TLARNKISSVWKEPISFVVTHLRPYTTYLFEVSAVTTEAGYIDSTIVRTPESVPEGPPQNCITGNVTGKAFSISWDPPAIVTGK
FSYRVELYGPTGRILDNSTKDLRFVFTHLTPFTMYDVYVAAETSAGVGPKSNLSVFTPPDVPGAVFDLQIVEVEATEIRVSWRK
PRQPNGIISQYRVKVSVLESGVILENTLLTGQDEYINNPMTPEIMNLVDPMIGFYEGSGEMSSDLHSLASFIYNSHPHDFPART
RVEDQRSPVVATRNQYMTDIAAEHLSYVIRRLVPFTEHTISVSAFTVMGEGPPTVLTVRTREQVPSSIQIINYKNISSSSILLY
WDPPEYPNGKITHYTIYAMELDTNRAFQMTTVDNSFLITGLKKYTRYKMRVAASTHVGESSLSEENDLFVRTPEDEPESSPQDV
KVTDVSPSELSLTWSPPEKPNGIIIAYEVFYQNADALFVKNTSTTNITLSDLKPYTLYNISIQSYTRLGHGNQSSSLLSVRTSE
TVPDSAPENITYKNISSEEIEIFFLPPRSPNGIIQKYTIYLKRSNSHEARTIETTSLTLTIGGLKKYTHYVIEVSASTLKGEGV
RSMPISILTEEDAPDSPPQNFSVKQLSGVTVMLSWQPPLEPNGIILYYTVYVWDKVSLKTINATEVSLELSDLDYHADYSAYVT
ASTRFGDGKTRSSVINFRTPEGEPSDPPKDVHYVNLSSSSIILFWTPPVKPNGIIQYYSVYYQNTSSTFVQNFTLLEVTQEPGN
VTVSARIYKLAVFSYYTFWLTASTLVGNGNKSSDVIHVYTDQDIPEGGVGNLTYESLSSTAINVSWTPPSQPNGLVFYYVSLNL
QQSPPRHRRPPLTTYENSIYFDNLEKYTDYIFKITPSTEKGFSETYTAQLHIKTEEDVPDTPPIINTFKNLSSTSILLSWDPPL
KPNGAILSYHLTLQGTHANRTFVTSGNHIVLEELSPFTLYSFLAAARTMKGLGPSSILFFYTDESAPLAPPQNLTLINYTSDFV
WLTWSPSPLPGGIVKVYSFKIHEHETDTVFYKNISGFQTDAKLAGLEPVSTYSISVSAFTKVGNGNQFSNVVKFTTQESVPDAV
QNIACVARDWQSVSVMWDPPRKANGIIIHYMITVEGNSTKVSPRDPMYTFTKLLANTSYIFEVRASTSAGEGNESQCNVSTLPE
TVPSVPTNTAFSNVQSTSVTLRWIKPDTILGYFQNYKITTQLRAQKCREWEPEECVEHQEVQYLYEANQTEDTVRGLKKFQWYR
FQVAASTNAGYGNASSWISTQTLPGPPDGPPENVRVVATSPFGINISWNEPAIITGPTFYLIDVKSVDNDNFNISFVKSNEENK
TTEINDLEVFTRYSVVITAFVGNVSGAYTDGKSSAEVIITTLESVPKDPPNNMTFQKIPDEVTKFQLSFLPPSQPNGNIQVYQA

LVYREDDPTAVQIHNLSIIQKTDTSVIAMLEGLKGGHTYNISVYAINSAGAGPKVQMRITMDIKAPARPKTKPIPIHDATGKLL
VTSTTITIRMPICYYNDDHGPIRNVQVLVAEAGAQQDGNVTKWYDAYFNKARPYFTNEGFPNPPCIEGKTKFSGNEEIYVIGAD
NACMIPGNEEKICNGPLKPKKQYLFKFRATNVMGQFTDSEYSDPIKTLGEGLSERTVEIILSVTLCILSIILLGTAIFAFARIR
QKQKEGGTYSPRDAEIIDTKFKLDQLITVADLELKDERLTRLLSYRKSIKPVSKKSFLQHVEELCTNNNLKFQEEFSELPKFLQ
DLSSTDADLPWNRAKNRFPNIKPYNNNRVKLIADVSIPGSDYINASYVSGYLCPNEFIATQGPLPGTVGDFWRMVWETRAKTLV
MLTQCFEKGRIRCHQYWPEDNKPVTVFGDILITKLMEDIQIDWTIRDLKIERHGDCMTVRQCNFTGWPEHGVPENTTPLIHFVK
LVRTSRAHDATPMVVHCSAGVGRTGVFIALDHLTQHIHDHDFVDIYGLVAELRSERMCMVQNLAQYIFLHQCILDLLSNKGGHQ
PVCFVNYSTLQKMDSLDAMEGDVELEWEETTM

PTPRQ.chicken XP_001235338.2| PREDICTED: phosphotidylinositol phosphatase PTPRQ
ENSGALT00000017794 [Gallus gallus]

MPDTGVFDGIYVTTNGGPNATFSLKSDGKLTVENLTPGTEYDFCVFTKSREMLSSSYRVTGVKTCLAAPLNIREGNVTDTSVQI
AWDRAEGDFQQYEVTCTNCASAFRVQKVKQETATFSNLVPGKLYSFTVRTEKEGFRDSVLVAKEIETVPSAVKYLNYSRDSESI
TVTWPPAQNKFDGYVLSIKSKIFNKENMLSSGVRMYKAECLLPGTDFLISIVTTSGLKRSHPTFLKISTCPDPPSDLQVLGQEE
NTVYLSWKLPRGGFDKFQLSYCLMNNEKPFTRTVYDSRTVVKNLAPGMEYTFQLRTIKGWDSSVAVEKNVITKPAGICNLALKM
VNTSSATLMWNPTKTNFTSYKASLSNTTFISKFMIPGAVSKFSVTNLTAGGIYNFTLQRLQGNIEGSPAFLEIVAEPAKPEGLK
FFNVSSNSFSLYWRLPYGHVDRFCVDLIPDHGSVVISDLGVREYQADFYNTTPGTTYNVTVSTVSSSTYSSPASRTVTTNVTNP
GPPAFLAGERVGSAGILLSWNTPQHPNGRILSYIVKYKEVCPWMQTAYTQVTSKPDSLEVLLTNLNPGTTYEIKVAAENSAGVG
VFSAPFLFQTAESAPGKVVNLTVEALNYSAVNLIWFLPRQPNGKITSFKISVKHARSGIVVKDVLVKVEDLLSGRLPECNDNSE
SFLWSTTTPSTTFGKSTIPSSTTVTASTEASSQMSAVWNEPISFVITNLRPYTTYLFEVSAVNTEAGYIDSTIVRTPESVPEDP
PQNFAKVNITSKSFSVMWDPPTIVTGKFSYRVELYGPSGHILDNSTKDLKFVFHNLVPFTMYDVYVGAETSAGVGPKTNLTVFT
PSDVPGAVSDLHLVEVEATYIKIVWRKPQQPNGIITQYRVKVHVQEKEVTLENIILIGKNKHLIDSLEPYMINENIEPSPTWNS
IETANELYEGSGEMFSSIQTVSPVILTTVSDDHLPAINGAAELHSTLDDQYATDILGEELSYIIKGLVPFTDYTISVSAFTAIG
EGPPSVLTVRTREQVPSSVQSISYKNISSSSVLLYWDPPANPNGKIIHYTVYAMELDTKRAFHTTTSNNSLLMTGLKKYTNYKM
RVAASTVIGESALSEENDIFVRTPEDEPDSPPQNVEIINVTATEINLKWLPPEQPNGLITHYEVLYSDSSDLFVRNTSSTNISL
TEMMPYTLYNISVRAFTRLGHGNQSSFPLLVRTSETVPNSAPENITYWNISSTEIELSFFPPSIPNGIIQTYTIYLKRINGTEE
RVINTTHLVLRITDLKKYTEYMIEVSASTMLGEGLRSAPLHILTDEDAPSSPPESLSVKQLSGVTVKLSWKPPLEPNGIILYYT
VYVWNKTSKRSVNVTETSLEFTDLENNSEYSAYVAASTRFGDGNIKSDTIIFRTSEGAPGDPPKDIVYKNLTSTSIMLFWSPPQ
KPNGNIRYYSVYFRNDSGIFIQNFTSHDNDSDVNMSPSAVLDDLAKYSHYTLWLTASTAVGDGNKTSEIIDVYTDQDIPDGPVE
NLVYQNISSSSVNVSWLPPSQPNGLVFFHVSLSLLQLGTNKILSFLTYNTSIIFDNLEKYTDYILKITPATDKGSSELHALSLH
IRTDEDVPESAPIIKTFSNLSTTSVMLSWDPPVKPSGIIISYDLNLFGSEKNISFSTTNNFIILEDLVPSTLYSIYTAARTMKG
SGPSSVLHFYTDESVPLAPPQNLTITNYTADSVWLKWEPSPQPNGVITRYNLKIYQNDTEKIFYQNISGSNNEAKLDGLKPFST
YFISVSAFTKLGNGNQFSNAVQFTTMESVPDVVQNVHCIATSWESIFMQWEPPASSNGVITYYIVTVEGSSTNFSSDTTLHTFQ
NLLSNISYQFKIKAATSAGDGEEQICNASTLPEEVPSAPRDIVFSNVQSTSVTLNWRSPKSIPGYFQNYKITTQLQSIYCSNWE
TKECIEDEIHQYLYEKGVNAQIEETVYGLKKYRWYRFAVAASTNAGYGSSSPWISTQTLPGSPDGPPENVTVLATSPHSINISW
SEPVVITGPTCYLIDITSVDNENYKAQFLRTNDEGKILEISDLKAFTRYSVVIIAFTGDVSAAAIEGKASSPVIVSTFEAVPED
PPNNVTFQKIPDEVTKFQVTFVPPSEPNGNIQVYQAMVYNEDDPAAIRIHNLSVIDKTDQSVTAMIEGLKGGHTYNVSVYAING
AGAGPKIQLKITMDIKEPPRPKKKPAPVYDTNGALLVTATTITIRMPVCYYSDDHGPIKKIQVLVVEAGAQHDGNVTKWHDAYF
NRPRPYFTNEGFPNPPCIEGKEDLSGKEEIYVIGADTTCMISGSQDKICNGPLKPRKQYLFKFRATNVKGQFTDSDYSDPVKTL
GEGRSGGSVEVILAVTLCILSVVLLVAAVYAFARIRQKQKEGGTYSPRDAEIIDTKFKLDQLITVADLELKDERFTRYSSFFFR
RKEIFVIQLLSYRKSIKPISKKSFLQHVEELCTNNNLKFQEEFSELPKFLEDLASTDADLPWNRSKNRFPNIKPYNNNRVKLMP
DAGIPGSDYINASYVSGYLCPNEFIATQGPLPGTVGDFWRMVWETRAKTLVMLTQCFEKGRIRCHQYWPEDNKPVTVFGDIVIT
KLVEDIQIDWTIRDLKIERHGDCMMVRQCNFTSWPEHGVPETTAPIIHFVKLIRASRAHDNTPMVVHCSAGVGRTGVYIALDHL
TQHINDHDFVDIYGLVAELRSERMCMVQNLAQYIFLHQCVLDLLTSRGSSQPICFVNYSALQKMDSLDAMEGDVELEWEETTM

PTPRQ.fish |XP_009291679.PREDICTED: phosphotidylinositol phosphatase PTPRQ ptprq-001 A8DZA4 ENSDART00000143740 [Danio rerio]

MTTTAAAQDTFRYGLTDHHPLQHQLDLHESSVSIHDLTPGSDYSFDIQSFLGSDFSQTTSKNISTRPAGVCSLFVSDVNVTSAT
VSWAAAAGGFDFYRVIVRNSSHKWTIDASPHLQEVTVSGLWSGCSYNTTVQRFRNTISGAAATINIHSVPASPEGLRVISVSPR
SFSLHWLASPGCEKTYQVQLYPDHGNINITTDTADNNVQAQVSSVTPGTSYTVTVNAVASSGFSSPVSRILVTTIESSAPSKPE
GERVGSTGILLSWRMPMPLDPSIHSFVIRYKEMCPYPDPSFTEITKSLDIPETLLNTLTPGATYNIKVAAVNKAGVGPFSQSLY
YKTAEAPPGLVSNLTAFAEDHTSVIVTWFLPVRINGLITKFAVKVKHARTGQIVRTKELNAEDIMNGALPHCNDAADILSRGTP
SPSQTSQLTSAVLPPITLSAVPPASIWSVPISVKIDELRPYTPYVFEVSAFTSDGEGQIASTMVRMPEAAPEDPPQNVVLRNIT
SKSVSLTWEPPKIITGRFSYVIQLHSSEGLISENSTIDQMFIYTGLTPYTTYYIHVMAKSAGAAGPAAVINITTLAEAPSAVSL
LKAEAVDSTSVRLSWRSPIQPNGLITHYRILVLYHDTLVQDITLRGQNLISPLRRNARSLDFTTESVFTLTSHETFSTLGRDFT
NTDTEAPSALPLTPSTPHTPPPWLTVTHDRTDASVQTQTDAVTEDLTTEHPPVTPVLPATEVSADLSSDQITHVVRRLSPFTEY
KFSVSAKNIIGEGPSTEVTVKTNEQVPSSVQNVSYQNLSSTSIRVSWEPPLNPNGKITHYAVYAQNLLTNQELRQMTDTTTAVL
TGLDKYSSYKVRVAASTAVGESPLTNEDYIYVVTLEDVPDSPPRGLTIVKTTSSTATLSWSPPEKPNGIIRMYEISYTNGTYSN
TVNSTSASATLRYLKPHTHYNVTVRAFTLLGHGEQISETLQMLSGEDVPGSPPYDLSYESIGSSSVNVSWSPPLLANGVILFYN
VEYWNATQSLNQTTHMPYIVLSNLRKYAHYRISVQAATQVGMGNHTSEILNITTLEDVPSSPPKFLIARKLSDTEVELSWEAPE
EANSEILYYIVRVWNLSTEFVANVTETSVVVSVDGPGQYNASVSSWTRLGDGGLLIYITFSTIESAPSDPPQDVVYTLLTISTV
RLSWRPPNEPNGIIQYYTIYYTDNNTEYTERVPGSEHQLLLSDLQAGQDYSVWMSSSTSVGDGGLFSPSLNFTTLEDVPSGPVH

NLSATIYSSTAVVISWDPPLEPNGRVYYQLSLQEAGITHPSINRTVNMTITKTTTDTIYLFTKLRKYFPYIINVTPATSAGSAV
NHTTMLHLRTDEDVPSSPPLSGSNKNLSSTSIQVSWFPPVEANGEIIEYAVNLQGPSTSNKNYTSETHLVLSELTPFTPYNLSI
AAVTRVGTGPPVVLSLHTDEAGPMSPPRNLTIFNHTANSVWLQWEPSPEPNGVVQLYGFRILELNTDSFRYQNSSDASTQAELG
GFKPHNSYEISVCTFTRAGNGDQYSLPVLFTTNESVSDAVGNLSCSGLDWDSVYMEWELPDHPNGEILYYLIRSGDLEDEAHPV
VLTHTVAHTLTGLSPHAFYLITVAAVNSAGVGEEANCSAHTPPESVPGPSHSLTVTDVTSDSVSVSWQRPVHVPGLLQGYNVEI
EQLSRNCERQQQQVESCMEAQILEWVEGETTRATLTSLLKYRQYRIRVVAFTRAGAGEPSEWIYTQTLAGNPDAPPGAISVVPS
ANGLKIEWDKPSVISGPTSYIIDITALDGSGYNITLVRHSEEIRTVIVGNLSAFTLHSVTITAFTGPLSNARRDGKASEPVLIR
TLEDEPKDPPKNVTLTVIPEEVTRVYVTFSPPDEPNGNISAYRVDIYRNGQLDFFINSLSVISNPNNTMTAIIDGLKGGFNYSI
RIAAVNGAPGFGPSSEVHVTTGVKAPPKPKKTPRAALNSAGVIISTSKTITIEMPECFFTDDHGPIQKVQVIVSEPAVMDYGNL
SNWKSVFLHPTAPYLTDDGFLNPECPKNSERMSSSTKTYVIGEDEGCLSEDAETLCNGPLKPKTHYVFKFRATNIRGQFTDSEY
SDKVRTADDRLLTRDEQIILGVLLSFFLALFLILIIYGSVKIHRRKKEGGTYSPRQAEIIETKFKLDQLIAVADLELKEEKINR
LLSYRKSLKPISKKSFLQHVEDLCANDNAKFQEEFAELPKLLQDLATSDADLPWNRSKNRFTNIKPYNNSRVKLLSEPGMPGSD
YINASFVSGYLCPNEFIATQGPLPSTVADFWRMIWETGTKTIVMLTQCFEKGRIRCHQYWPEDNKPVTVFADIIITKLTEDVRP
DWTVRALKVERHGSYMIVHHFNYTSWPEHGVPESSSTLVQFVKAVRSNRGHENTTIVVHCSAGVGRTGVFIALDHLIQHLRDHE
FVDIYGLVAELRSERMCMVQNLAQYMFLHQSTLDLLSAKGNSQSIWFVNYSALEKMDSLDAMEGDVELEWEETTM

PTPRB.human PTPRB-001 ENST000003344142200 [Homo sapiens]

MEAEFYMVILTCLIFRNSEGFQIVHVQKQQCLFKNEKVVVGSCNRTIQNQQWMWTEDEKLLHVKSALCLAISNSSRGPSRSAIL
DRCSQAPRWTCYDQEGFLEVENASLFLQKQGSRVVVKKARKYLHSWMKIDVNKEGKLVNESLCLQKAGLGAEVSVRSTRNTAPP
QILTTFNAVPDGLVFLIRNTTEAFIRNAAENYSQNSSERQHPNLHMTGITDTSWVLSTTQPFSSTTEETGLAEPERCNFTLAES
KASSHSVSIQWRILGSPCNFSLIYSSDTLGAALCPTFRIDNTTYGCNLQDLQAGTIYNFRIISLDEERTVVLQTDPLPPARFGV
SKEKTTSTSLHVWWTPSSGKVTSYEVQLFDENNQKIQGVQIQESTSWNEYTFFNLTAGSKYNIAITAVSGGKRSFSVYTNGSTV
PSPVKDIGISTKANSLLISWSHGSGNVERYRLMLMDKGILVHGGVVDKHATSYAFHGLTPGYLYNLTVMTEAAGLQNYRWKLVR
TAPMEVSNLKVTNDGSLTSLKVKWQRPPGNVDSYNITLSHKGTIKESRVLAPWITETHFKELVPGRLYQVTVSCVSGELSAQKM
AVGRTFPDKVANLEANNNGRMRSLVVSWSPPAGDWEQYRILLFNDSVVLLNITVGKEETQYVMDDTGLVPGRQYEVEVIVESGN
LKNSERCQGRTVPLAVLQLRVKHANETSLSIMWQTPVAEWEKYIISLADRDLLLIHKSLSKDAKEFTFTDLVPGRKYMATVTSI
SGDLKNSSSVKGRTVPAQVTDLHVANQGMTSSLFTNWTQAQGDVEFYQVLLIHENVVIKNESISSETSRYSFHSLKSGSLYSVV
VTTVSGGISSRQVVVEGRTVPSSVSGVTVNNSGRNDYLSVSWLLAPGDVDNYEVTLSHDGKVVQSLVIAKSVRECSFSSLTPGR
LYTVTITTRSGKYENHSFSQERTVPDKVQGVSVSNSARSDYLRVSWVHATGDFDHYEVTIKNKNNFIQTKSIPKSENECVFVQL
VPGRLYSVTVTTKSGQYEANEQGNGRTIPEPVKDLTLRNRSTEDLHVTWSGANGDVDQYEIQLLFNDMKVFPPFHLVNTATEYR
FTSLTPGRQYKILVLTISGDVQQSAFIEGFTVPSAVKNIHISPNGATDSLTVNWTPGGGDVDSYTVSAFRHSQKVDSQTIPKHV
FEHTFHRLEAGEQYQIMIASVSGSLKNQINVVGRTVPASVQGVIADNAYSSYSLIVSWQKAAGVAERYDILLLTENGILLRNTS
EPATTKQHKFEDLTPGKKYKIQILTVSGGLFSKEAQTEGRTVPAAVTDLRITENSTRHLSFRWTASEGELSWYNIFLYNPDGNL
QERAQVDPLVQSFSFQNLLQGRMYKMVIVTHSGELSNESFIFGRTVPASVSHLRGSNRNTTDSLWFNWSPASGDFDFYELILYN
PNGTKKENWKDKDLTEWRFQGLVPGRKYVLWVVTHSGDLSNKVTAESRTAPSPPSLMSFADIANTSLAITWKGPPDWTDYNDFE
LQWLPRDALTVFNPYNNRKSEGRIVYGLRPGRSYQFNVKTVSGDSWKTYSKPIFGSVRTKPDKIQNLHCRPQNSTAIACSWIPP
DSDFDGYSIECRKMDTQEVEFSRKLEKEKSLLNIMMLVPHKRYLVSIKVQSAGMTSEVVEDSTITMIDRPPPPPPHIRVNEKDV
LISKSSINFTVNCSWFSDTNGAVKYFTVVVREADGSDELKPEQQHPLPSYLEYRHNASIRVYQTNYFASKCAENPNSNSKSFNI
KLGAEMESLGGKCDPTQQKFCDGPLKPHTAYRISIRAFTQLFDEDLKEFTKPLYSDTFFSLPITTESEPLFGAIEGVSAGLFLI
GMLVAVVALLICRQKVSHGRERPSARLSIRRDRPLSVHLNLGQKGNRKTSCPIKINQFEGHFMKLQADSNYLLSKEYEELKDVG
RNQSCDIALLPENRGKNRYNNILPYDATRVKLSNVDDDPCSDYINASYIPGNNFRREYIVTQGPLPGTKDDFWKMVWEQNVHNI
VMVTQCVEKGRVKCDHYWPADQDSLYYGDLILQMLSESVLPEWTIREFKICGEEQLDAHRLIRHFHYTVWPDHGVPETTQSLIQ
FVRTVRDYINRSPGAGPTVVHCSAGVGRTGTFIALDRILQQLDSKDSVDIYGAVHDLRLHRVHMVQTECQYVYLHQCVRDVLRA
RKLRSEQENPLFPIYENVNPEYHRDPVYSRH

PTPRB.mouse 201 ENSMUST00000092167 [Mus musculus]

MLRHGALTALWITLSVVQTGVAEQVKCNFTLLESRVSSLSASIQWRTFASPCNFSLIYSSDTSGPMWCHPIRIDNFTYGCNPKD
LQAGTVYNFRIVSLDGEESTLVLQTDPLPPARFEVNREKTASTTLQVRWTPSSGKVSWYEVQLFDHNNQKIQEVQVQESTTWSQ
YTFLNLTEGNSYKVAITAVSGEKRSFPVYINGSTVPSPVKDLGISPNPNSLLISWSRGSGNVEQYRLVLMDKGAIVQDTNVDRR
DTSYAFHELTPGHLYNLTIVTMASGLQNSRWKLVRTAPMEVSNLKVTNDGRLTSLNVKWQKPPGDVDSYSITLSHQGTIKESKT
LAPPVTETQFKDLVPGRLYQVTISCISGELSAEKSAAGRTVPEKVRNLVSYNEIWMKSFTVNWTPPAGDWEHYRIVLFNESLVL
LNTTVGKEETHYALDGLELIPGRQYEIEVIVESGNLRNSERCQGRTVPLAVLQLRVKHANETSLGITWRAPLGEWEKYIISLMD
RELLVIHKSLSKDAKEFTFTDLMPGRNYKATVTSMSGDLKQSSSIKGRTVPAQVTDLHVNNQGMTSSLFTNWTKALGDVEFYQV
LLIHENVVVKNESVSSDTSRYSFRALKPGSLYSVVVTTVSGGISSRQVVAEGRTVPSSVSGVTVNNSGRNDYLSVSWLPAPGEV
DHYVVSLSHEGKVDQFLIIAKSVSECSFSSLTPGRLYNVTVTTKSGNYASHSFTEERTVPDKVQGISVSNSARSDYLKVSWVHA
TGDFDHYEVTIKNRESFIQTKTIPKSENECEFIELVPGRLYSVTVSTKSGQYEASEQGTGRTIPEPVKDLTLLNRSTEDLHVTW
SRANGDVDQYEVQLLFNDMKVFPHIHLVNTATEYKFTALTPGRHYKILVLTISGDVQQSAFIEGLTVPSTVKNIHISANGATDR
LMVTWSPGGGDVDSYVVSAFRQDEKVDSQTIPKHASEHTFHRLEAGAKYRIAIVSVSGSLRNQIDALGQTVPASVQGVVAANAY
SSNSLTVSWQKALGVAERYDILLLNENGLLLSNVSEPATARQHKFEDLTPGKKYKMQILTVSGGLFSKESQAEGRTVPAAVTNL
RITENSSRYLSFGWTASEGELSWYNIFLYNPDRTLQERAQVDPLVQSFSFQNLLQGRMYKMVIVTHSGELSNESFIFGRTVPAA
VNHLKGSHRNTTDSLWFSWSPASGDFDFYELILYNPNGTKKENWKEKDVTEWRFQGLVPGRKYTLYVVTHSGDLSNKVTGEGRT

APSPPSLLSFADVANTSLAITWKGPPDWTDYNDFELQWFPGDALTIFNPYSSRKSEGRIVYGLHPGRSYQFSVKTVSGDSWKTY
SKPISGSVRTKPDKIQNLHCRPQNSTAIACSWIPPDSDFDGYSIECRKMDTQEIEFSRKLEKEKSLLNIMMLVPHKRYLVSIKV
QSAGMTSEVVEDSTITMIDRPPQPPPHIRVNEKDVLISKSSINFTVNCSWFSDTNGAVKYFAVVVREADSMDELKPEQQHPLPS
YLEYRHNASIRVYQTNYFASKCAESPDSSSKSFNIKLGAEMDSLGGKCDPSQQKFCDGPLKPHTAYRISIRAFTQLFDEDLKEF
TKPLYSDTFFSMPITTESEPLFGVIEGVSAGLFLIGMLVALVAFFICRQKASHSRERPSARLSIRRDRPLSVHLNLGQKGNRKT
SCPIKINQFEGHFMKLQADSNYLLSKEYEDLKDVGRSQSCDIALLPENRGKNRYNNILPYDASRVKLCNVDDDPCSDYINASYI
PGNNFRREYIATQGPLPGTKDDFWKMAWEQNVHNIVMVTQCVEKGRVKCDHYWPADQDPLYYGDLILQMVSESVLPEWTIREFK
ICSEEQLDAHRLIRHFHYTVWPDHGVPETTQSLIQFVRTVRDYINRSPGAGPTVVHCSAGVGRTGTFVALDRILQQLDSKDSVD
IYGAVHDLRLHRVHMVQTECQYVYLHQCVRDVLRAKKLRNEQENPLFPIYENVNPEYHRDAIYSRH

PTPRB.chicken PTPRB-201 ENSGALT00000016330 [Gallus gallus]

MSSPNQQWQWMADDKLFHMKSGQCLSISTRSALSSRSIIINCSQAVRWTCHEEDGLLKVANSSLFLTKQGQKVMAKQSKKYLHT
WMQLEASETGKPVYVNLCSKQAAHNSLSTGLPLPNFPDLWMGTMNSTTTTSSPLNTTTSRPDNLPASSSTQMSIRNVTEHFTKN
FIENLYFDLKSAVTEKPTVPTTPPQYSSTTEETKSAYLLTDRGKTHSCSFIYALFQSPNTHKQLWQRRGMQVPAPYTEETNSEH
SVPPVFRFHVDKPQLSNLRANLASSLAKCLQSVSVKDCTTHLLKAHLFSTTLEIIMLTILYTYITTNITSKSALLRWSAAAHAC
NFNLTGRSEDGRPAGCRPASAGNGSYGCTVRDLEAGTWYHLRIEPLADGEAANVTLQTDPLPPPRFEINKEKTTFTSLQVRWDP
SPGKVDLYNIVVFDHSNKKLQESSIPGRFSKTEETFTGLVPGNKYNIVLTAVAGNKSTPEFRINGSTAVPSPVKNIQVIVKTDS
IHASWSPGSGHVDLYKLVLFDNHGTVHESHQENPLTSYIFSGLTAGHLYNLSVITQAAELESTAFRIVRTAPAEVLDLTVTNDD
SFDTLKVKWRRPSGGVDFYNITLSHLRSVKEVKTLQPAVTETHFDKLTPGRLYQITARTISGELFTDKMATGRTFPQKVSELKA
GGGGWLRTLRVNWLPPAGDLERYHLLLWNHSALVLNTTLGKNVTEYLVRDVGLIPGRQYEVEVVVESGDLQSRASCTGRTAPEP
VLQLRVKHANESSLSVMWVTPVAEWDSYVVSLGDRDLTVIKKGLGKEAKEFTFNELVPGRKYTATVTTISGILSNWTSVEGRTV
PAQVTGLTVASQGSTNSLFTNWTKALGDVDSYQVLLIHENVVIKNETVPSETNEYHFYPLKPGGLYSVVVTTVSGGISSRQTIA
EGRTVPSSVTGVTVNNSGRSDYLSVSWLPASGDVDSYLVTLSHDDQIIQTLTISKSLSECSFSSLTPGTLYNVMITTKSGKYEN
HSFSRERTVPSGVQGLTVSNSARSDYLKVSWLHASGSFDSYEVIIKNNNDFIQTKSVPKDENECVFTNLVPGRQYSVTVSTRSG
KYETSERVFGRTMPESVKGLTLSNRSTEDLRVTWSKAEGDVDKYEIQLLYNDMKIFPPIFLGNTIEECWFTALTPGRLYKIVVL
TISGDAQRATFIEGLTIPSAVRNINVSPNGMTDSLKVSWTPGGGDVDSYTVTIFQQNHQLDSRSVSKDVSEHTFHDLEAGEQYR
VVVQSNSGALHNSLAAFGRTIPASVQELLADHAYSSHSLLVSWQKAPGVAERYDILLLNEQGVLLSNKSEPATAKQHKFEDLLP
GKKYKIHILTVSGGLFSKRAETVGRTVPAAVTNLKVTKNTTDQLSFSWTTSQGELDSYDIFLYNPDKSLHDRISGEQHLQQCSF
QNLRQGRMYRMVIVTHSGDLTNESSVFGRTAPAPVVGLKASNRNMTDSLWFTWNPAAGDVDFYEFNLYNPNGTQKESLQGKDLK
EWYFQGLVPGRKYTLVVVTHSGDLINTANAEGRTAPSPPNTVSFTDVANTSLSITWLGPPDWTDYDDFELQWLPKDPLTVFNPY
SSSKSKVRIIYGLRPGRLYKFSVRTVSGDSWKTYSQSQSESVRTRPDKIQSLHCRPQTSTAIACSWTPPDSDFDGYSVECKKLD
TREVEFSKRIEKDRTLLNIMTLVPHKRYLVSIKVHSADMTSEVVEDSTITMIDRPPQPPPDIRVNKKEVLITKSSINFTFNCSW
FSDTNGAVKYFTVVVREADGSEGPKPDEQHPLPSYLEYKHNDSIRIYQTNYFASRCAENPDSDYKSFDIKLGGEMENLGGKCDP
DQQKFCDGPLKPRTAYRISIRAFTQLFSEDPKELPQPLFADTFFSLPITTEAEPLFGVIEGVSAGLFLIVMLVAVTALFVCRQK
VSNGHERPTARLSIRRDKPLAVHLNLGQKGNRKTSSPIKVSHFEAHFTKLQADSNYLLSKEYEDLKDVGRNQTCDIALLPENRG
KNRYNNILPYDTSRVKLSNVDDDPCSDYINASYIPGNNFRREYIATQGPLPGTKDEFWKMAWEQNVHNIVMVTQCVEKGRVKCD
HYWPLDQDSLYYGDLIVEMLSESVLPEWTIREFKICSEEQLDSTRLIRHFHYTVWPDHGVPETTQSLIQFVRTVRDYINRTPDT
GPTVVHCSAGVGRTGTFIALDRILQQLDSKDTVDIYAAVHDLRLHRVHMVQTECQYVYLHQCVRDVLRARKLRSEQENPLFPIY
ENVNPEYHRDAVYSRH

PTPRB.frog xenpopus ptprb-201 ENSXETT00000024858 [Xenopus tropicalis]

MLRYAFGLSLWIVLNLIKNVEPVKCTINNTDVTVTSQSIYLEWTSPGSHCNFSLTCSSHNFWEITCNPIQKSNDSYECTLSGLE
AGTVFDLRIVSLQDGEMKNLSLQTDPLPPSNFVVNKNSVTSSSVQANWAPSSGKVDLYKVKLLDSKKEIVQEIEVSGISLTKEV
TFKNLTPGTKYSIEITAISGNKRSPTSVINASTVPSTVKSIDANSKTDTIDASWKPGHGNIDSYKLILLNEDRKITEVVLEQNI
TSYSFHGLLPGYVYNLTVISEAAGLRTWNSKLIRTAPAEVQAISVQSDGRTDALKVQWKEPRGKLDFYNVTLSDTGSIKYSRTV
HPGTPTEITFTGLTPGHLYQVGISTIAGELSTSRMATGQTIPEKVADLKAVSNGLRSLRLSWVPPSGDWDKYNIVLYNNTAVLV
NTTIDKKYREYVIQDIGLIPGRQYEAVVIVESGKFKNLARCKGRTAPPAVMDLRLKHANESSLTIMWVMPVAEWDNYVVSLADR
DLTPVNKVLSKEAKEFTFNHLAQGRKYIATVTSISGDLSSRASVEGRTVPAQVTNLNVENQGTTSSLHADWTKAPGDVDSYQVL
LIHEKIVIKNETVSNEINKYSFHSLKPGGLYSVVVTTVSGGISSRQTVSEGRTGRKLLSIISSNTGSLTPFLHQSWSSNSLRYI
GWTSLICQGKFLAKDLFFAWPGIECSFPCVIKGALYFVRMDYQQTVGTNRYPRDNEAIPLVVVNTCCINCLSSSYRKLSVIESI
SPINKRNNIKKRSVLSVSSEYTHPRLRHSGCVTTRANSGMLKLQLPGAGLLVPEAVKKLTLAERSSEELHVTWSRADGDVDHYE
IQLLFNDMKVFPPITLNNTADEYKFTSLTPGRLYKIVVLTFSGEAQRAMFVEALTVPSTVKNIHISPNGMTNSLKVNWTPGGGD
VDSYTVSIFHHNSPIGSHTTSKHIFEHTFQNLNPGELYKVDVQTNSGSLHKSLESFGRTIPATVHSLSADNAYSSHLLIVSWQS
AKGVTDRYDILLLSDHGIVITNKSVPIMAKSHKFEDLVPGKKYRIQVFTVSGGLFSAAVETEGQTVPAAVSDVKITGNSTDSLS
FTWNSSEGELDSYGIFLYNPDNSLHDRKSGNPDLRHCSFQGLLPGRLYKMVIVTHSGSLTNESSIHGRTVPAPVSSLQVSNRNT
SESLWFTWAPALGDVDIYELILYNPNGTEKEKIQDKDLTESHFQNLVPGRLYTLVIFTHSGDLFNKATAVGRTEAPQPPNSVSF
ADVKINSLSIMWLGPPDWTDYDDFELQWSPKDLLVVVNPYSIGRSKGRIINGLYPGRQYTFGVQSVSGNTGKTLSPAIFGTVRT
KPDKIHHLHCRPQTSTAISCSWTPPDSDFDGYSVECRRNGSDDVEFSKRIEKDKSSITISTLVPHKRYVVSIKVHSADMTSQVI
EDSAITMIDRPPPPPLLIRVNKKDTFISKSSIHFRFNCSWFSDTNGAVKYFTVIVSEADGNDNQRPEASLPLPSYADYKTNKST
KIYQTSYFPSRCAENPDYNIQSYKIKLGTGMELLGGKCDQNENKYCDGPLSPRTSYRISVRAFTQLFTEEMRTFPEPLYSDTFF
SLPITTEAGSLFFNKNITDLPLQIFPQTKAMILVVLCRYEQDPMTTHLSSQIERIPSVHLNVGHIQIGDRISSRPILTAQFEEH

FSKLQTDSNYLLSREYENLKDFGRDQSSDTALLPENRGKNRYSNILPYDSTRVKLANVDDDPCSDYINASYMPGINFRREYIAT
QGPLPATKDDFWKMVWEQNVHIIVMVTQCTERGRAKCDHYWPMDQDSYYYGDLIVQMLSESVLPEWTIREFKICSEDQIDAPRL
VRHFHYTVWPDHGVPETTQSLIQFVRTVRDYINRTPGSGPTVVHCSAGVGRTGTFIVLDRMLQQVDTVDSVDIFGAVRDLRIHR
MYMVQTECQYVYLYQCVRDVLRARKLRNEQDNPLFPIYENVNPEYHRDAVYLRH

PTPRB.fish Zebrafish ptprb-202 ENSDART00000127299 NP_001186063.1| FN428722 Danio rerio

MLRNKAFHAAVWAACGILSTMVVDATECSIEIVKVISSTESIRLTLDNANVKCQYTVSVKDRHTDSKGCRQDREHSKCQIESLD
PGTWYHLDVISTLDEKQQSQRAVTLQTRPSAVENLQVSGDANSLDVSWQPGKGKTERYWIVLIDSSGRDSAWNSTVASTATSYT
MKGLISGRLYNITVVTEVGELQNSASTQAQTAPASVSKLRTENNGDRNSIRVLWDKASGDVDSYLVSLTTPGSNSIEKVLPPDD
TYVVFENLSPGKGYQVSVSTRSGALSNKTWITGKADPGKVSDLVIENLSVRGALKISWTPPSGEWEHIRVALSNGSEVLRNQTV
GRTAKEILLSGLNLLPGRVYRMAVSVENGGLANTVIYEGEIGLPPVSQLHVRHSDETSLSALWAHAASSSSRDGYIIQLFQSNT
STVIQTRNLSRDMRECTFNVLTPGRLYDITVTTTAKIIRSSATLQGRTQPLKVNHLKLSNKGSTDSLNASWEKPLGDLDFYHML
LLRDQQTVHNITASANTTSRLLPFLRPGALHKILVTTISGSQTSKLAEAECRTVPAAVSDITVTNSGPDFLNVSWKAAEGDVDN
YVVMLKDQEKIVHTLATSKATTECVFRSLVSGRLYSISIATHSGSYRNQTLLQERTKPSTVQNPTAIHSARDDFLKLYWNHASG
DYDYYVVTIEHNGTRLQSQKLNRTQSDCAFSDLVPGRLYNLTVSTWSGQHYSAVSIYGRTFPGAVGNLSLTERGGTFLRVSWTS
APGDVDHYELQILFNDTQVSAAVNLSSAIGEHLFSALTPGRLYKIVLSTHSGSYQRAEILEGRTVPSQVQSVHLSAGTADGSLR
VSWSSADGDLDFYSVSLFQETHLQDSRRVPKHITQAEFYNLIPGQLYSVTVQSVSGSQTNNSTTTGRTEPSTVTGLRADNELST
HSLLVSWNPAVGVHDGYRLWLLDDGGTNIHNASVPAASRHHLFENLTPGRRYQAHVRTLSGTAESKDVVAEGQTRPSAVSALHV
CSNTSSDLSFCWASAVGWVDGYELYLYDRDETLHYHTTLGSDALGWSFTLLQPGTHYKMTITSKSGKLSNQSSVWAHTAPASVP
ELHVENQGQTDSLLLSWTPAPGGLTGYSVTVDGREQRVGPEVTQVVFHSLVAGRLYLATVQTWSEDLSNSTTAVGRTVPAAPSS
VSVSCSGGSVDMKWHVPDTGDYEDFEVTWFPQDTLYISHLRPTQRILEGLHPGRLYNISLRTVSGKRHSPVTYSRPVYHTIRTP
PLPAPSIHCFPLSSTSVSCSWTPPHSDYDGFVVQCHRQGSGKAVYTHTLGNHTLSQQFDRLEPFKNYTIYVAVMSGDKQSSTVK
NSVITMIDRPPVPPVTVRVNEHSAVITHFTIRFKFNCSWFSDANGAIRYFTIIATESNDVDNGLPEQRHPLPSYLDYRQNHSIK
AYQTGYFHSTCAEGSDGKVQVFEINLGAGMKHLGGACKLDPESIQHGSHLCDGPLRSRTSYRLSVRAFTQLFDEENREFPHPLY
TDTYLSLPLLTQSAPRSGLTGGITAALFLITMVLALTALLIYRKRAHKIAVQESPVMKMCKWKALPTSQMCLRIRSPVQAANFE
SHLAKLQSDSSYLLSEEFEGLKDVGRIQTQNAARLLGNRNKNRYNNILPYDSTRVRLSCLEDDPCSDYINANYIPGNNFRWEYI
ATQGPLPGTKDDFWRMVWEQNVHSLVMVTQCVERGMVKCDHYWPTDSEPLCYGDIVVQLLSEKVFPEWTIRDFKISCESQLRYP
RMVRQFHYTIWPDHGVPDTTQSLIQFVRTVRDFINRTNSPGISVVHCSAGVGRTGTFIVLDRALQQLDRNCTVDIYGCVFDLRL
HRSYMVQTECQYAYIHQCVRDVLRARKLGCERDNPLFPIYNNII

>PTPRJ.human ENSG00000149177 [Homo sapiens]

MKPAAREARLPPRSPGLRWALPLLLLLLRLGQILCAGGTPSPIPDPSVATVATGENGITQISSTAESFHKQNGTGTPQVETNTS
EDGESSGANDSLRTPEQGSNGTDGASQKTPSSTGPSPVFDIKAVSISPTNVILTWKSNDTAASEYKYVVKHKMENEKTITVVHQ
PWCNITGLRPATSYVFSITPGIGNETWGDPRVIKVITEPIPVSDLRVALTGVRKAALSWSNGNGTASCRVLLESIGSHEELTQD
SRLQVNISGLKPGVQYNINPYLLQSNKTKGDPLGTEGGLDASNTERSRAGSPTAPVHDESLVGPVDPSSGQQSRDTEVLLVGLE
PGTRYNATVYSQAANGTEGQPQAIEFRTNAIQVFDVTAVNISATSLTLIWKVSDNESSSNYTYKIHVAGETDSSNLNVSEPRAV
IPGLRSSTFYNITVCPVLGDIEGTPGFLQVHTPPVPVSDFRVTVVSTTEIGLAWSSHDAESFQMHITQEGAGNSRVEITTNQSI
IIGGLFPGTKYCFEIVPKGPNGTEGASRTVCNRTVPSAVFDIHVVYVTTTEMWLDWKSPDGASEYVYHLVIESKHGSNHTSTYD
KAITLQGLIPGTLYNITISPEVDHVWGDPNSTAQYTRPSNVSNIDVSTNTTAATLSWQNFDDASPTYSYCLLIEKAGNSSNATQ
VVTDIGITDATVTELIPGSSYTVEIFAQVGDGIKSLEPGRKSFCTDPASMASFDCEVVPKEPALVLKWTCPPGANAGFELEVSS
GAWNNATHLESCSSENGTEYRTEVTYLNFSTSYNISITTVSCGKMAAPTRNTCTTGITDPPPPDGSPNITSVSHNSVKVKFSGF
EASHGPIKAYAVILTTGEAGHPSADVLKYTYEDFKKGASDTYVTYLIRTEEKGRSQSLSEVLKYEIDVGNESTTLGYYNGKLEP
LGSYRACVAGFTNITFHPQNKGLIDGAESYVSFSRYSDAVSLPQDPGVICGAVFGCIFGALVIVTVGGFIFWRKKRKDAKNNEV
SFSQIKPKKSKLIRVENFEAYFKKQQADSNCGFAEEYEDLKLVGISQPKYAAELAENRGKNRYNNVLPYDISRVKLSVQTHSTD
DYINANYMPGYHSKKDFIATQGPLPNTLKDFWRMVWEKNVYAIIMLTKCVEQGRTKCEEYWPSKQAQDYGDITVAMTSEIVLPE
WTIRDFTVKNIQTSESHPLRQFHFTSWPDHGVPDTTDLLINFRYLVRDYMKQSPPESPILVHCSAGVGRTGTFIAIDRLIYQIE
NENTVDVYGIVYDLRMHRPLMVQTEDQYVFLNQCVLDIVRSQKDSKVDLIYQNTTAMTIYENLAPVTTFGKTNGYIA

>PTPRJ.mouse Ptprj-201 ENSMUST00000168621 [Mus musculus]

MTRGGGRGSSRGRGSRELGATRGGWAPLAPPREAPASLRPRPLRARRARLRRVAAAAAAAMSPGKPGAGGAGTRRTGWRRRRRR
RRLETETRAPGFGHTAGRVPGTFQGAQGMKPAARETRTPPRSPGLRWALLPLLLLLRQGQVLCAGAAPNPIFDIEAVVSPTSVL
LTWKHNDSGASECRIENKMESNLTFPVKNQTSCNITGLSPGTSYTFSIISVTTNETLNKTITTEPWPVSDLHVTSVGVTQARLT
WSNANGTASYRMLIEELTTHSSVNISGLKPGTNNSFAFPESNETQADFAVAEEVPDANGTKRIPVTNLSQLHKNSLVSVDPPSG
QDPSLTEILLTDLKPDTQYNATIYSQAANGTEGQPRNKVFKTNSTQVSDVRAMNISASSMTLTWKSNYDGSRTSIVYKIHVAGG
THSVNQTVNKTEAIILGLSSSTLYNITVHPFLGQTEGTPGFLQVYTSPDQVSDFRVTNVSTRAIGLAWRSNDSKSFEIFIKQDG
GEKHRNASTGNQSYMVEDLKPGTSYHFEIIPRGPDGTEGLSSTVNGSTDPSAVTDIRVVNISTTEMQLEWQNTDDASGYTYHLV
LESKSGSIIRTNSSQKWITVGSLTPGTLYNVTIFPEVDQIQGISNSITQYTRPSSVSHIEVNTTTTTAAIRWKNEDAASASYAY
SVLILKTGDGSNVTSNFTKDPSILIPELIPGVSYTVKILTQVGDGTTSLVPGWNLFCTEPEPVTSFHCEVVPKEPALVLKWACP
FGMYTGFELGVRSDSWDNMTRLENCTSDDDTECRTEVAYLNFSTSYNISIATLSCGKMALPAQNICTTGITDPPTPDGSPNITS
VSHNSVKVKFSGFEASHGPIKAYAVILTTGEAAQPSADVLKYTYEDFKRGASDTYVTYLIRIEEKGQSQGLSEVLNYEIDVGNQ

STTLGYYNGRLEPLGSYRACVAGFTNITYNLQNDGLINGDESYVSFSPYSEAVFLPQDPGVICGAVFGCIFGALAITAVGGFIF
WRKKRTDAKNNEVSFSQIKPKKSKLIRVENFEAYFKKQQADSNCGFAEEYEDLKLIGISLPKYTAEIAENRGKNRYNNVLPYDI
SRVKLSVQTHSTDDYINANYMPGYHSKKDFIATQGPLPNTLKDFWRMVWEKNVYAIVMLTKCVEQGRTKCEEYWPSKQAQDYGD
ITVAMTSEVVLPEWTIRDFVVKNMQNSESHPLRQFHFTSWPDHGVPDTTDLLINFRYLVRDYMKQIPPESPILVHCSAGVGRTG
TFIAIDRLIYQIENENTVDVYGIVYDLRMHRPLMVQTEDQYVFLNQCVLDIIRAQKDSKVDLIYQNTTAMTIYENLEPVSMFGK
TNGYIA

PTPRJ.chicken   PTPRJ-202 ENSGALT00000045335 NP_989952.1| [Gallus gallus]

MRRLPLLPPCPLLLLLLLPAEVRCTTACTDDCSLKNVTEEMGTSSNDELSVNATSGNRRLSEDVSLPGRAMSDQNSVAQPRAVL
DLKTEYVGVTSVNLTWTVNDTASDSYTYRIEVRNGSSINNKTSDITDAEITGLIPGTLYTFTVFAVAADGQTAGEGASISLYTK
PSPVLDLKAEYVGVTSVNLTWTVNDTASASYTYRIEVTSDSSIDSLTSSVTMAEITGLIPGTLYSFKVFAVAADNRTEADGASI
SLYTKPSPVLDLKAEYVGVTSVNLTWTVNDTASASYTYRIEVTSDSSIDSLTSNVTMAEITGLIPGTLYNFTVFAVAADNRTEA
DGAFTSLYTKPTPVTDLKAEHGVTSVSLNWMVNDTASDSYTYRIEVRNGHSVNNKTSNIPETEITGLNPGTLYTFTVFAVAADG
ETEGEGASISVYTKPRAVLHLKTEYVGVTSVNLTWTVNDTDSASYTYRIEVRNGSSINNKTSDITDAEITGLDPGTLYIFTVFA
VAADGQTAGEGASISLYTKPSMVLNLKAEYVTMTSVNLTWMVNDAESASYTYRIEVAHESLINETMSNVTKSIVTYLIPGTSYN
FTVFAIAADNQTEGEGASISQNTVPSSVNAFQCEAVANMSYLTLKWNCPYGGYSGFDIEIFNGTWTKKQQSQFCGREGSEEIFK
TEPLDYYKTYTVSVTTVSDGLTSLPVQKICKTSITDPPVPNKAPLVKAVSHNSLSVEFPDFESVNGPLKAYAVMIVTEAEGCLP
SKSDLDYTYNDFKQKMTATYVTYVIDVEEISSSSHSQNGHNIVDVGKGNTMYGYENGPLIPLHSYRASVAGFTNINFTVANKIM
GEQSYVSFSPCSEVVSLPQDPGVIAGAVIGCLLAILAVVAIGGYIFWRRRRKDKRNTEVSFSPIKSKMIKVENFESYFKKQQAD
SNCGFAEEYEELKSAGVHQPKFAAEIAENRGKNRYNNVLPYDISRVKLSNPSCTTDDYINANYMPGYSSKKAFIAAQGPLPNTI
EDFWRMIWEKNIYSIVMLTKCVEQARTKCEQYWPDKQSKSYGDIIVTMVSEVVLPEWTIRDFNVENADTMESHTVRQFHFTSWP
DHGVPETTDLLINFRHLVHEYSSQNPIDSPILVHCSAGVGRTGTFIAIDRLIQQIEMENTVDVYGVVYDLRMHRPLMVQTEDQY
VFLNQCVMDIIRSQKEKKTDLIYQNTTAMAIYENFTPGPAFGKANGYHA

PTPRJ.fish   FN428723 NP_001129714.1| [Danio rerio]

MGQHYRLQAIVSRTTVLFVVLVSIHHGHCTDCSLCQYKTSTTTSEINVFVGNSSFCIAQNASFTNSSQTNITVTGLLPGNTYFL
IISCTSVCCENFSSSPAQVQNITVVNYTNSSVTVAWTVPDGHVDSYTVNCSTQSIPNINDNNTTISSLPPGCLYNLTVTSISGV
LTNVSNIVQFATKPNPPEAVRVSGQTFSSISLLWSTPLSMSGVAVSYGVSYVPVQPGVNLPLTQTTSSNMTNITNLLSGTQYTI
TVVTNGVNNLSSSFINLIGYTVPKIVQNLAVSNVSTNSVSLTWLPPNGTSSLFSYNINISSLGQTFYTTSNSYQITQLQPGTQY
NCYVTTLIVAGNISGPSQFIQCNTKPLPVSNLKAIPVGTTVILLSWTPSTALYLYNVSANGTWSTYNSSEGANVTGLTPGNNYT
FTVITVANGLSSEPVTISAFTGLGKAIIISAIGNTQTMTVQWTPPAGTVSLYSARILLMDGTVLNTKNQTKTTSMMFSDLLPGT
QYSVIVSSISGPVKQDSDSVTNATLPTPPGEISTTQSTNTLNISWAQPLNMSSVSYYFLLQYNNSTLINCSQNYTSLTGLNAGF
PYNIIMWTVGAMNYSSSSKSLTAFTNPSVVTNVTVNEFTETSVTLSWQQNDPQQSGYSYLLNYKTINGTSNQTTVNNNTVKLEQ
LQSASQYNISIIALTPGGTKSDPQFITACSRPNRVNNVSSVALNVSTVRLSWARPLQYNQLVSYQVLVSNCTKNSMNMSTSTEM
ITVTNLQPGTLCQISIYSVACGLLGQPVNTSVLTMPSTVQPVVNSQGSNNSLLVLWSHPDGGLDMYILNISSDGWSDFSFLNST
ENNYTFTQLKAATLYTVTLTTVKGSFQETSGAVVSATYPNSPGMITVLFKNTHSVLLHWDIAQNMTPGSFNYSLSFWSSKNNSL
YLTPNNTLLLDGLQSGTPYNVSLATVGPLNLQSESMRYNVTTMPDPVKNLKVVSTTTNTISVTWKMVDVPKYVVSVNNAVKITS
SNNMTIHNLQPSTQYNISVQSSTFDNTEGEAVCLQDCTDAAPVENVTCVGPNLTLPMLNLFWNHPLGGYMNFEVNLSSSISEKT
KNLNYNFTGLKYNKTYSVTLRTLGCGKSSTVKNISCTTGFTNPVVPKITAASVSEQQYNKFALVIQSDAFNDSNGTVCSYGLLV
SSGSFDCLDNNQNYNQCLLKTYDDWKAKQSDTFLAVVKTNVSKSELETIIIGDGSKWNKYTNGELNAKGTYNFAIVAFTLLEVK
DGKVDVSKSYYSVSAYQSVTLPENPVVIGGAAAGVGVTVIIVVIIIGIVACTRKKRKEVSNSVPIHSLSDPIKVEDYEAYYKRQ
RADSFCGFAEEFEDLRPVGINQSKTVAVFPENKAKNRYNNVLPYDSSRVKLSVLSSPFDDYINANYMPGYITKKEFIAAQGPLP
GTVNDFWRLIWEKNVHTIVMLTKCNEQGRVKCEEYWPAEMKTFSNLTVTTISDIPLEDWTLRDFEVKNMKTAEIRSVRHFHFTA
WPDHGVPETTELLINFRHLVREHMDEYSRHSPTLVHCSAGVGRTGTFIAIDRLIFQIERDGVVDVYGIIHDLRMHRPLMVQTED
QYVFLNQCAMDIIKSRTGNNVDLIYQNTAALTIYENFEPLKKGKNGYHKA

PTPRO.human ENSP00000281171 [Homo sapiens]

MGHLPTGIHGARRLLPLLWLFVLFKNATAFHVTVQDDNNIVVSLEASDVISPASVYVVKITGESKNYFFEFEEFNSTLPPPVIF
KASYHGLYYIITLVVVNGNVVTKPSRSITVLTKPLPVTSVSIYDYKPSPETGVLFEIHYPEKYNVFTRVNISYWEGKDFRTMLY
KDFFKGKTVFNHWLPGMCYSNITFQLVSEATFNKSTLVEYSGVSHEPKQHRTAPYPPQNISVRIVNLNKNNWEEQSGNFPEESF
MRSQDTIGKEKLFHFTEETPEIPSGNISSGWPDFNSSDYETTSQPYWWDSASAAPESEDEFVSVLPMEYENNSTLSETEKSTSG
SFSFFPVQMILTWLPPKPPTAFDGFHIHIEREENFTEYLMVDEEAHEFVAELKEPGKYKLSVTTFSSSGSCETRKSQSAKSLSF
YISPSGEWIEELTEKPQHVSVHVLSSTTALMSWTSSQENYNSTIVSVVSLTCQKQKESQRLEKQYCTQVNSSKPIIENLVPGAQ
YQVVIYLRKGPLIGPPSDPVTFAIVPTGIKDLMLYPLGPTAVVLSWTRPYLGVFRKYVVEMFYFNPATMTSEWTTYYEIAATVS
LTASVRIANLLPAWYYNFRVTMVTWGDPELSCCDSSTISFITAPVAPEITSVEYFNSLLYISWTYGDDTTDLSHSRMLHWMVVA
EGKKKIKKSVTRNVMTAILSLPPGDIYNLSVTACTERGSNTSMLRLVKLEPAPPKSLFAVNKTQTSVTLLWVEEGVADFFEVFC
QQVGSSQKTKLQEPVAVSSHVVTISSLLPATAYNCSVTSFSHDSPSVPTFIAVSTMVTEMNPNVVVISVLAILSTLLIGLLLVT
LIILRKKHLQMARECGAGTFVNFASLERDGKLPYNWRRSIFAFLTLLPSCLWTDYLLAFYINPWSKNGLKKRKLTNPVQLDDFD
AYIKDMAKDSDYKFSLQFEELKLIGLDIPHFAADLPLNRCKNRYTNILPYDFSRVRLVSMNEEEGADYINANYIPGYNSPQEYI
ATQGPLPETRNDFWKMVLQQKSQIIVMLTQCNEKRRVKCDHYWPFTEEPIAYGDITVEMISEEEQDDWACRHFRINYADEMQDV

MHFNYTAWPDHGVPTANAAESILQFVHMVRQQATKSKGPMIIHCSAGVGRTGTFIALDRLLQHIRDHEFVDILGLVSEMRSYRM
SMVQTEEQYIFIHQCVQLMWMKKKQQFCISDVIYENVSKS

PTPRO.mouse Ptpro-201 ENSMUST00000077115 [Mus musculus]

MGHLPRGTLGGRRLLPLLGLFVLLKIVTTFHVAVQDDNNIVVSLEASDIVSPASVYVVRVAGESKNYFFEFEEFNSTLPPPVVF
KATYHGLYYIITLVVVNGNVVTKPSRSITVLTKPLPVTSVSIYDYKPSPETGVLFEIHYPEKYNVFSRVNISYWEGRDFRTMLY
KDFFKGKTVFNHWLPGLCYSNITFQLVSEATFNKSTLVEYSGVSHEPKQHRTAPYPPRNISVRFVNLNKNNWEEPSGSFPEDSF
IKPPQDSIGRDRRFHFPEETPETPPSNVSSGSPPSNVSSAWPDPNSTDYESTSQPFWWDSASAAPENEEDFVSALPADYDTETT
LDRTEKPTADPFSAFPVQMTLSWLPPKPPTAFDGFNILIEREENFTDYLTVDEEAHEFVAELKEPGKYKLSVTTFSSSGACETR
KSQSAKSLSFYISPTGEWIEELTEKPQHVSVHVLSSTTALMSWTSSQENYNSTIVSVVSLTCQKQKESQRLEKQYCTQVNSSKP
VIENLVPGAQYQVVMYLRKGPLIGPPSDPVTFAIVPTGIKDLMLYPLGPTAVVLSWTRPILGVFRKYVVEMFYFNPTTMTSEWT
TYYEIAATVSLTASVRIASLLPAWYYNFRVTMVTWGDPELSCCDSSTISFITAPVAPEITSVEYFNSLLYISWTYGDATTDLSH
SRMLHWMVVAEGRKKIKKSVTRNVMTAILSLPPGDIYNLSVTACTERGSNTSLPRLVKLEPAPPKSLFAVNKTQTSVTLLWVEE
GVADFFEVFCQQLGSGHNGKLQEPVAVSSHVVTISSLLPATAYNCSVTSFSHDTPSVPTFIAVSTMVTEVNPNVVVISVLAILS
TLLIGLLLVTLVILRKKHLQMARECGAGTFVNFASLEREGKLPYSWRRSVFALLTLLPSCLWTDYLLAFYINPWSKNGLKKRKL
TNPVQLDDFDSYIKDMAKDSDYKFSLQFEELKLIGLDIPHFAADLPLNRCKNRYTNILPYDFSRVRLVSMNEEEGADYINANYI
PGYNSPQEYIATQGPLPETRNDFWKMVLQQKSHIIVMLTQCNEKRRVKCDHYWPFTEEPIAYGDITVEMVSEEEEEDWASRHFR
INYADEAQDVMHFNYTAWPDHGVPPANAAESILQFVFTVRQQAAKSKGPMIIHCSAGVGRTGTFIALDRLLQHIRDHEFVDILG
LVSEMRSYRMSMVQTEEQYIFIHQCVQLMWLRKKQQFCISDVIYENVSKS

PTPRO.chicken PTPRO-201 ENSGALT00000037455 [Gallus gallus]

MLPQFTIAGHGSEPGGGRRGAAARGRGAEEPQPPPPGRSRGPRPPRSPMPDRRRRGARGGPGARSCLLWLFVLLKNAEPFQVIV
QEDNCIVVSLEAFDVVSSSSVYVVKIAGESKNYFFQFEEFNSTLPAPVIFNAKYHGLYYIVTLLVVNSNMVSKSARSITVLTKP
LPVSSVSIYDYKPSPETGVLFEIQYPEKYNVFTRVNISYWEGKDYRTMLYKDFFKGKTVFNHWLPGICYSNITFQLVSEATFNK
STLVEYSGVRHEPKQHRTVPYPPRNISVQIVPINRNNWEEHSGNFAEESFMGQQEIISKEKLSRFPSDPPEIPVVNTTAAWPDY
RSNSTEYETTSQPYWWSNEAESSEGEEEFVNAVPRDYGASEANASGKLTAEPAPFLPVQMVLTWLPPKPPTAFDGFHINIEREE
NSTELLTVSEDTHKFVAELKEPGKYKLSVTTFSSSGSCEVRGSQSAKTLSFYISPTGEWIEELTEKPQHVTVTVLSSTTALTSW
TASHESGSNGTIVSVVSLTCQKQKESQRLEKHYCTEVNSSSSLIENLVPGAQYQVVVYLRKGPLVGPPSDPVTFSIVPTGIKDL
TLYPLGPTAVVLSWNRPFLGVFRKYVVEMFYFNPSTMTSEWTTYYEIAATISLTASVRIANLLPAWYYNFRVTMVTWGDPELSC
CDSSTISFITAPVAPEITSIEYYNHLLYVTWTYGGDGTDLSHSRMLHWMVIAEGKKKIKKSVTRSVMTAVLSLPPGDIYNLSVT
ACTERGSNTSLPQLVKLEPAPPKSLFAVNKTQTSVTLLWVEEGVADFFEVYCQQAGSNQEVKVQEPVTVSSHVVTISSLTPATS
YNCSVTTFSHNSPSVPTYIAVSTMVTEMNPNIVVISVLAILSILLIGLLLVTLVVLRRKHLQMARECGAGTFVNFASLERDGKL
PYNWRRSVFAFLTLLPSCLWTDYLLAFYINPWSKNGLKKRKLTNPVQLDDFDGYIKDMAKDSDYKFSLQFEELKLIGLDIPHFA
ADLPMNRCKNRYTNILPYDFSRVRLVSMNEEEGSDYINANYIPGYNSPQEYIATQGPLPETRNDFWKMVLQQKSQIIVMLTQCN
EKRRVKCDHYWPFTEDPIAYGDITVEMLSEEEHTDWVYRNFRISYADEVQDVMHFNYTAWPDHGVPTANAAESILQFVQMVRQK
SVKSKGPMIIHCSAGVGRTGTFIALDWLLQHIRDHEFVDILGLVSDMRSYRMSMVQTEEQYIFIHQCVQLMWQKKKQQFCISDV
IYENVSKS

PTPRO.xenopus ptpro-202 ENSXETT00000048747 NM_001113813.1 PARTIAL [Xenopus tropicalis]

TFQVALTQDNCLVISLESPDVLSTKSVYLVKITGESKNYFFQFEEFNSTLPSPLVFNATYHGLYYIITLIAVNSNMASRSIKSI
TVLTKPLPVSHVVIHDYRPSPQTGVVFEVQYPEKFNVFSRVNISYWEGSHFRTVLYKDFFKGKTVFNHWLPGTCYGNITLQLVS
EASFNKSTLVEYSGVGHRPQFHRTVPFPPRNISVQILRLNLSFTDLLNGKNNEDSLKKYQDFTEPESSAEMFEDQNERSTFPSL
YNSTDPQYKNNTLPEATSQPFWWANETLPPDTDGFVNGVLPDYEDPPVGSSVPNSSSLSFKLLISWLPPKAPTAYDGFNIYIFR
DGNASAVTAVDESAHEFVAEMREPGKYWVEITSFSSTGSCEIRESSAAKAISFYIKLLASAGELTPWGPRSTEPYKLLLYSDVI
IIIILGLYELHKSHIQISPSCSLTCPKQKESQRLERHYCKEVNSPKPVDHIETPNRVLQKVGYLRVTPRKVAGQISDPPHFLVN
PPGVTDLLLYPLGPSAVVLGWRRPYLGAFRKYMVEMFYFNPAAMSSEWTPYYEVAATVSLTASVVPFAPRVPCAPHVPFAPRVP
FAPRVPCAPHVPFAPRVPCAPHVPFAPRVPCAPHVPCALXEVRGDINISRRILYTALFPSGSHEMLQGVSRSGTATVISVPPGD
IYNITVTACTERSSNTCAFTLLQSDPAPIRTLFAVNKTQTSVTLLWVEEGIADYFEVSCWQNREPGAETNQKDPVAVFSHIVTI
SSLQPDTSYNCSVTSISHRTPSAPTFLTVSTLVAELNPNVVVISVLALLSVLLIALLFLMLIVLRRKHLQMSRDCGAGTFVNFA
SLEKDGKLPYNWRRSIFAFLTVLPSCLWTDYLLAFYINPWNKNGLKKRKLTPVQLDDFDLYIKEMSKDSDYKFSLQFEELKMVG
LDIPHFAADLPSNRGKNRYTNILPYDCSRVKLISLEADEGADYINANYIPGYSAAQEYIATQGPLPETRNDFWKMILQQKCQVI
VMLTQCNEKRRIKCDHYWPFTPEPVNYGDITVEMASEEEQSDWAQRVFRVSYADETQCVTHFNFTAWPDHGVPTVNAAESVLQF
VQVVRQKALKSKGPVTVHCSAGVGRTGTFIALDWLMQHIRDHEFVDILGLVSELRSHRMSMVQTEEQFVFIHQCAQLMWKKKRQ
QFRISDVIYENVSKS

PTPRO.fish zebrafish ptpro-204 ptpro-204 ENSDART00000122021 [Danio rerio]

MSSARARFFFIIFIFIQSAAGFRVQLQDGVRMLMSLDDGDIGPGVSEYAVRVSGEPLTHTLLFQQAADAHTLPEPLLFNASYHG
LLYSISLMTDGRTHTTRSIITQPLPLDSVEMWDYAPSPETAVVFQIRSPDRNIFTRVNISYTEGHQRRYMLYKDFLHGKTVFKH
WLSGVCYSNITFQLISEASVNRSALLSRSDITHNPQQHRTVPNPPLNVSLKILHLSGRGPPGPVLTGAILKNSHNASVVRRERD
LPEYEEQEPTSDTLNTESNLTHTQLNQSESENESESEPVTAEPTLNSSTQSLWAWQTVSPAPTEEEEEGFVNALVPEYEDSNEP
GSALGIPLEPAVMPAVMPTPLAPVLLQLRWSPPAPHTAYDAFNIYIYRNGNSTETATVDENTHEFLAELSESGTYRIHVTTLSS
AGDCEARESSANTAFTFYLSPSGEWMEQPQERPQAVSVKMLDSSTAAVSWAPSTHTYNGSLISVQSLTCLRPSISQRMELNYCS
EENITSDIISSLTPGAQYRVVVYHTNGPLVSPASEPVIIDIEPTGVRDLVVYPLSPSAVILSWQRPYNVAFRKYVLQTFFFNSA
TQTAQWSTYYEIAATASVIASVRVTDLLPAWFYNFRVTMVTWGDPPLSCCDTSTVSFITAPEAPHISSVEFSHGSVFVRWTYGD
LFTDLTHSRMLHWQVVAEGKKSARRRFSVDVTRSVMKASLALPAGDIYNLTVTACTERSCNTSAPHIMKLDPAPPRSLYAVNAS
DTSVTLIWAEEGVVDHYLITCRALGAHAEQKKVREPLVTSAHVLTVSGLQASTTYNCSVRSSSYSSSSDPVHITVSTTVREMNP
SVAAISALAVLSVLLISLLVLFLLVLRKKHMELSRECGAETFVNFASFERDGKLPYNWRRSLFAFLTLLPSCLWTDYLLAFYIN
PWSKTALKKRKLTSPVQLSDFEAYLKDMGKDSAYKFSLQFEELKSVGLDLSHEAADLPINRPKNRYTNILPYDFSRVKLISLHN
DEGSDYINANYIPGYNSPREYIATQGPLPDTRNDFWKMVLQQKVHIIVMLTQCNERRRVKCDHYWPFSDEPVAYGEISVEMLAE
TDSPEWTIRSFRLAYADETQDVLHFNYTSWPDHGVPTVNAIESILQFVQIVRQQVNRSKGPIVVHCSAGVGRTGTFISLDRLMQ
HIQEHEYVDVLGLVSDMRSHRLSMVQTEEQYVFIHQCVLLMWKKKKQQSHTSDVIYENVSKS

PTPRH.human ENSP00000365528 [Homo sapiens]

MAGAGGGLGVWGNLVLLGLCSWTGARAPAPNPGRNLTVETQTTSSISLSWEVPDGLDSQNSNYWVQCTGDGGTTETRNTTATNV
TVDGLGPGSLYTCSVWVEKDGVNSSVGTVTTATAPNPVRNLRVEAQTNSSIALTWEVPDGPDPQNSTYGVEYTGDGGRAGTRST
AHTNITVDGLEPGCLYAFSMWVGKNGINSSRETRNATTAHNPVRNLRVEAQTTSSISLSWEVPDGTDPQNSTYCVQCTGDGGRT
ETRNTTDTRVTVDGLGPGSLYTCSVWVEKDGVNSSVEIVTSATAPNPVRNLTVEAQTNSSIALTWEVPDGPDPQNSTYGVEYTG
DGGRAGTRSTAHTNITVDRLEPGCLYVFSVWVGKNGINSSRETRNATTAPNPVRNLHMETQTNSSIALCWEVPDGPYPQDYTYW
VEYTGDGGGTETRNTTNTSVTAERLEPGTLYTFSVWAEKNGARGSRQNVSISTVPNAVTSLSKQDWTNSTIALRWTAPQGPGQS
SYSYWVSWVREGMTDPRTQSTSGTDITLKELEAGSLYHLTVWAERNEVRGYNSTLTAATAPNEVTDLQNETQTKNSVMLWWKAP
GDPHSQLYVYWVQWASKGHPRRGQDPQANWVNQTSRTNETWYKVEALEPGTLYNFTVWAERNDVASSTQSLCASTYPDTVTITS
CVSTSAGYGVNLIWSCPQGGYEAFELEVGGQRGSQDRSSCGEAVSVLGLGPARSYPATITTIWDGMKVVSHSVVCHTESAGVIA
GAFVGILLFLILVGLLIFFLKRRNKKKQQKPELRDLVFSSPGDIPAEDFADHVRKNERDSNCGFADKYQQLSLVGHSQSQMVAS
ASENNAKNRYRNVLPYDWSRVPLKPIHEEPGSDYINASFMPGLWSPQEFIATQGPLPQTVGDFWRLVWEQQSHTLVMLTNCMEA
GRVKCEHYWPLDSQPCTHGHLRVTLVGEEVMENWTVRELLLLQVEEQKTLSVRQFHYQAWPDHGVPSSPDTLLAFWRMLRQWLD
QTMEGGPPIVHCSAGVGRTGTLIALDVLLRQLQSEGLLGPFSFVRKMRESRPLMVQTEAQYVFLHQCILRFLQQSAQAPAEKEV
PYEDVENLIYENVAAIQAHKLEV

PTPRH.mouse Ptprh-202 ENSMUST00000166650 [Mus musculus]

MARAGGNCGVWRSLVLLGLYGCSVVRAAGTSVTVDRHAPASSYEFSMWVEKDGVSSSPQIPVTTAAPNPVRNLRVEGQNNISIS
LSWEPPDQSSLQGLTYWTQCSRHGGQTETRNTTDTSVTVDGLDPGSSYECSVWVEKDGLYSKNETLNTSTAPNPVRNLRVEGQN
NISISLSWEPPDQPSLQGLTYWAQCSRHGGQTETRNTADTSVTVDGLDPGSSYECSVWVEKDGVYSTNETLNTSTAPNPVRNLR
VEGQNNISISLSWEPPDQPSLQGLTYWAQCSRHGGQTETRNTTDTSITVDGLDPGSSYECSVWVEKDGVYSTNETLSNTTAPNP
VRNLRVKSQNNFSISLSWEPPDQPSLQGLIYWAQCSRHGGQTETRNTTDTSVTVDGLDPGFLYKCSVWVEKDGVYSTNETLNTS
TVPISASNPVRNLRVEGQNNFSISLSWEPPDQSSLQGLTYWAQCSRHGGQTETRNTADTSVTVDGLDPGSSYECSVWVEKDGVY
STNETLNTSTVPAAVNITSCISTSGGYGVLLTWSCPSGGYESFEVKVGRKWRSENGSLCGKGVTVSDLEPAQSYTATVTTVFKD
LKAQSLSTTCHTESAAIIAGAIVGILLLFILVGLLIVFLKRRRKKRQPKEVPKDLVCSCPGDILAKDFADHVRENEKDSNCGFA
EEYQQLALEGQGQSQITASALENRSKNRYRNVLPYDWSRVPLQPLQEEPGSDYINASFMPGLWSPKEFIATQGPLPNTVGDFWR
MVWEQQSHTLVMLTNCMESGRVKCEHYWPLDAQPCIHGQLQVMLISEEASENWTVRHLQLFHMKEQQTLSLRQFHYLAWPDHGV
PYSPDPLLAFRKMLRQWMDQTTDGGPPIVHCSAGVGRTGTLIALDVLLRQLECEGLVGPFSFVKKMRESRPLMVQTEAQYVFLH
QCILKSLQKPAPALVPEEAMYENVASLVYENASAIMAHESEFSASGC

PTPRH.xenopus XP_004916623.1| [Xenopus tropicalis]

MEYDEALTGICNVQILLKAMRTGCYVTLGYVPIRSHRFQKRLLSGVPSPVQSVSVSNITTTNVTLSWVPPNDINKDTYTYSITV
YNGSSPWDWPKNISGTSTQVTGLIPGVFYTFSVFTVTSNGTMSSQAGSTSGTTVPSPVQSVSISNIMTTNVTLSWVPPNDINKD
TYTYSITVYNGSTSWNGPTNISGTSTQVTGLIPGVYYTFSVFTVTSNGTKSSQAGSTSGTTVPSPVQSVSISNIMTTNVTLSWV
PPNDINKDTYTYSITVSNGSSPWNGPKNISGTSTLVTGLIPGVFYTFSVFTVTSNGTMSSQNGSTSGTTGPSLVTTVSIIKQTN
NSVTISWTPPQDINQATYMYSINVTNSAGSWNVNSSTNVSSKEVLDLLPGVTYTFCVYTVTSDGTKSSDCSPDSSTTRPTLVKD
VTVKSQTTDSVTLSWTTPNDINKGSYYYLITTACGTTTCNVNSTVNANGATVASLLPGLTYQFSVYTVTSDGTNSSAFASTSGT
TAPNAPTSVSGNPVNTTALSVNWTPPNDLNKNNYTYTVFWQRNNESLNQSITSNTTIIGGLTAGNQYNVSVISVINNVPSVKAV
AYLQTNPEVPGGFKIVTITNSSVNVSWTIPSGGSFSGIEINAVSGTSNLTKQFTGPSSEGILDGLIPGTAYNLILRSFSSYPSG
SSGRISRRATTNNIITYSNPIIEKVQLDPNTVTGITCTKVGGGYQLKVAFVCPSGSYSSIKILADGNEKETIPAQNCSQGATVQ
SLQPATKYIITVETVADSRQVSDFIICYTDNVGVIVGSIFGVLLFLLVVGLIAYFVLRKRRGSKNPFKKERGNMQKINIVRMPS
VMKSAFPDYYQRQHADSDFGFAEEYQQLSNVGINQSKLAAELSENRSKNRFTNVLPYDHSRVRLNRIDADETSDYINANYMPGY
NSSKEFIASQGPLPNTSADFWRMIWENQVSTIVMLTNCMENGRVKCEHYWPLDYTPCTYGDITVTVTSEMILPDWTVRDFTLKH

AKQQGNKHARHFHFTVWPDHGVPENTTTIVEFRNLVREYMDLKRSSGPTVVHCSAGVGRTGTLIALDYLIQKMEKEQRIGIYSF
VQKMRQNRPLMVQTESQYVFLNKCMLDLIQNPPDENIYENQIGGDLIYENAS

PTPRH.fish zebrafish ptprh-202 ENSDART00000114768 [Danio rerio]

MELVLAFALLCVLSVSGQATLPDVNDVRITHRTETELTFQWDKAGGSNSYSYILRRDGIDSDIITAGTVNQVTYKVSDLSSGTN
YNWTLFTVFGDQRSEGKNFSAATVPPNVESVSVIDQSETTITLQWKKDKAEYSYNLTYGDKVDTPPSPQSTVEYTVTSLSPGTN
YSFTLYTVFEGLKSSGFSFFAVTVPSKITGFSVIGRSETALTFQWTKEKDEYTYELDKSDANTQISFTDTTVVCLITSLSPGTE
YSYNLYAVFEGVKSQAKGISEVTVPSNVENVSVVEANDTDVILQWNAVPNEQNKYNYILKYNDVEESVDFTTENEIKHHVSGLI
PATNYNFTLHTEFYSQNSTGYNFNHTTFLSSVTEVRVNRSLTELTVEWNKLNQNNVYNYTLLRSDGAEIDFTGSAVVGDVIKHK
YSSLKPGTVYSFTLFTVSNNVRSSEHSFKSITALDCASFNWKVTNTSIVADVYGCTYVTAQNSSGSGKNASIEGDKVNLRDLYP
GESYTVSLFYNLESKMLTQCSQRLTLVPYVVSNLKCEYHSGGYGLAVMWDYPDGVVDVVEVDVNKKSFNHSHGSDEPTQQLVTG
LQAAQWYKVKATSFSGARRSETILINCQTDPAGVIAGVLVFFLLVIIICAGVFVWFRYKSARNNGPDASADLKVSKKNYKTIAC
DKFPEHFRNMSRDDNRAFSAEYDDLSSVGVEQSKVAALLPENKDKNRFSNVLPYDTSRVHLTINKAGDSDYINANYMPGYGNAS
REYIAAQGPLPSTVNDFWRMIWEKKSSTIVMVTNCTEGGRVKCEQYWPLDYTPCLYENLLVTVKSENKSQSWTLREFNVKNKMT
SETRTVRHFHFTAWPDHGVPRGTEELIQFRDLIRQHIESHFSTGPTVVHCSAGVGRTGTLIALDVLLQQLNREKAVGVAAFVQE
MRLNRPLMVQTESQYVFLHQCILDSLQTKFVQQSEPIYQNTDTIYVNAMALKDFENSSHT

>PTPRF.human ENSG00000142949 [Homo sapiens]

MAPEPAPGRTMVPLVPALVMLGLVAGAHGDSKPVFIKVPEDQTGLSGGVASFVCQATGEPKPRITWMKKGKKVSSQRFEVIEFD
DGAGSVLRIQPLRVQRDEAIYECTATNSLGEINTSAKLSVLEEEQLPPGFPSIDMGPQLKVVEKARTATMLCAAGGNPDPEISW
FKDFLPVDPATSNGRIKQLRSGALQIESSEESDQGKYECVATNSAGTRYSAPANLYVRVRRVAPRFSIPPSSQEVMPGGSVNLT
CVAVGAPMPYVKWMMGAEELTKEDEMPVGRNVLELSNVVRSANYTCVAISSLGMIEATAQVTVKALPKPPIDLVVTETTATSVT
LTWDSGNSEPVTYYGIQYRAAGTEGPFQEVDGVATTRYSIGGLSPFSEYAFRVLAVNSIGRGPPSEAVRARTGEQAPSSPPRRV
QARMLSASTMLVQWEPPEEPNGLVRGYRVYYTPDSRRPPNAWHKHNTDAGLLTTVGSLLPGITYSLRVLAFTAVGDGPPSPTIQ
VKTQQGVPAQPADFQAEVESDTRIQLSWLLPPQERIIMYELVYWAAEDEDQQHKVTFDPTSSYTLEDLKPDTLYRFQLAARSDM
GVGVFTPTIEARTAQSTPSAPPQKVMCVSMGSTTVRVSWVPPPADSRNGVITQYSVAYEAVDGEDRGRHVVDGISREHSSWDLV
GLEKWTEYRVWVRAHTDVGPGPESSPVLVRTDEDVPSGPPRKVEVEPLNSTAVHVYWKLPVPSKQHGQIRGYQVTYVRLENGEP
RGLPIIQDVMLAEAQWRPEESEDYETTISGLTPETTYSVTVAAYTTKGDGARSKPKIVTTTGAVPGRPTMMISTTAMNTALLQW
HPPKELPGELLGYRLQYCRADEARPNTIDFGKDDQHFTVTGLHKGTTYIFRLAAKNRAGLGEEFEKEIRTPEDLPSGFPQNLHV
TGLTTSTTELAWDPPVLAERNGRIISYTVVFRDINSQQELQNITTDTRFTLTGLKPDTTYDIKVRAWTSKGSGPLSPSIQSRTM
PVEQVFAKNFRVAAAMKTSVLLSWEVPDSYKSAVPFKILYNGQSVEVDGHSMRKLIADLQPNTEYSFVLMNRGSSAGGLQHLVS
IRTAPDLLPHKPLPASAYIEDGRFDLSMPHVQDPSLVRWFYIVVVPIDRVGGSMLTPRWSTPEELELDELLEAIEQGGEEQRRR
RRQAERLKPYVAAQLDVLPETFTLGDKKNYRGFYNRPLSPDLSYQCFVLASLKEPMDQKRYASSPYSDEIVVQVTPAQQQEEPE
MLWVTGPVLAVILIILIVIAILLFKRKRTHSPSSKDEQSIGLKDSLLAHSSDPVEMRRLNYQTPGMRDHPPIPITDLADNIERL
KANDGLKFSQEYESIDPGQQFTWENSNLEVNKPKNRYANVIAYDHSRVILTSIDGVPGSDYINANYIDGYRKQNAYIATQGPLP
ETMGDFWRMVWEQRTATVVMMTRLEEKSRVKCDQYWPARGTETCGLIQVTLLDTVELATYTVRTFALHKSGSSEKRELRQFQFM
AWPDHGVPEYPTPILAFLRRVKACNPLDAGPMVVHCSAGVGRTGCFIVIDAMLERMKHEKTVDIYGHVTCMRSQRNYMVQTEDQ
YVFIHEALLEAATCGHTEVPARNLYAHIQKLGQVPPGESVTAMELEFKLLASSKAHTSRFISANLPCNKFKNRLVNIMPYELTR
VCLQPIRGVEGSDYINASFLDGYRQQKAYIATQGPLAESTEDFWRMLWEHNSTIIVMLTKLREMGREKCHQYWPAERSARYQYF
VVDPMAEYNMPQYILREFKVTDARDGQSRTIRQFQFTDWPEQGVPKTGEGFIDFIGQVHKTKEQFGQDGPITVHCSAGVGRTGV
FITLSIVLERMRYEGVVDMFQTVKTLRTQRPAMVQTEDQYQLCYRAALEYLGSFDHYAT
