# Supplementary material for: The Tetraspanin-Associated Uroplakins Family (UPK2/3) Is Evolutionarily Related to PTPRQ, a Phosphotyrosine Phosphatase Receptor
Source: PLoS One. 2017 Jan 18;12(1):e0170196. doi: 10.1371/journal.pone.0170196 (PMC5242461; doi:10.1371/journal.pone.0170196)
Supplement: S2 File — (PDF) [file pone.0170196.s007.pdf]

```
#NEXUS

BEGIN data;
	DIMENSIONS Ntax=40 NCHAR=347;
	FORMAT DATATYPE = Protein GAP = - MISSING = ? interleave;
	MATRIX

upk3A_human		-----------------VNLQPQLASVTF-------ATNNPTLTTVALEKP--------
LCMFDSKEALTGTHEVYLYVL-----------------VDSAISRNASV-QDSTNTPLGSTFLQTE------------------------
GGRTGPYKAV--AF-------DLIPCSDLPSLDAIGDVSKA--SQILNAYLVRVGAN-----GTCLWD------
PNFQGLCNAPLSAATEYRFKYVLVNM----------STGL---VEDQ-
TLWSDPIRTNQLTPYSTIDTWPGRRSGGMIVITSILGSLPFFLLVGFAGAIALSL---------------
upk3A_mouse		-----------------VNLQPQLASVTF-------ATNNPTLTTVALEKP--------
LCMFDSSEPLSGSYEVYLYAM-----------------VDSAMSRNVSV-QDSAGVPLSTTFRQTQ-----------------------
-GGRSGPYKAA--AF-------DLTPCGDLPSLDAVGDVTQA--SEILNAYLVRVGNN-----GTCFWD------
PNFQGLCNPPLTAATEYRFKYVLVNM----------STGL---VQDQ-
TLWSDPIWTNRPIPYSAIDTWPGRRSGGMIVITSILGSLPFFLLVGFAGAIILSF---------------
Upk3A_chiken -----------------QSMKPQLAAPEL-------ATNNPTLTTVALEKP--------
FCMFDSSLHPNKSYAIYLYVM-----------------KSSANTISSVV-TDSSSKPLDSTFQQTH------------------------
GGHLGPYKAA--SF-------DVPNCVSPPRLADAGDINKV--SDVLKQYLFRVGDD-----GTCLYD------
PNFLDVCNPPLAPDTTYRFKYVLVDN----------TEGI---VKDQ-
TLWSDPIKTRKAKLPMKIDIWPGRRSGSMIVITSILSVSVFLLLAGLLASVFSAL---------------
upk3A_frog		-----------------GADMPLLANSDF-------FSLNPTQTTIALEQP--------ICMFKSA-------
-VNVYLI-----------------GIVAGAPNTPL-YDGNKKVNASTYSGTQ------------------------GGKTGPYIVA--KL----
---PNQQCINIQALSNMADPTQV--QSILSKYVVRVGAD-----VTCLTN------
PNFVGYCNAPLQGNTQYSFKYLFTDS----------GD-I---VQSE-
TSWSLGITTVNGKASSTIDTWPGRRSGGMIVLTSILSTLMFFVFIAYVIGFAYSI---------------
UPK3A_fish		---------------EALKVKPEAVSPRL-------LRFNPTQSTVSLAKP--------LCVFDSVK-
PTNEMMVDVYVV-------------------HSLSATLT-------FETGKTYKETN------------------------GGTETPYKAT--
SF-------GIPNCTSPPNPADLSVPQRI--DKTLDEYLVRIGSN-----PTCVGE------
PEAEAFCNAPLSDGTSYRFKYLLVNG----------TT-----TIAE-
TEWSESILTRKALSPDEIDTWIGKRSGGGIVVTVILSLLLFLLLGAAIFMGVLDV---------------
lamprey1		-----------------SGTKPQLVSSTA-------VPYNPTETTIVWSKP--------FCVFQKPV-
PTTQYVVDVY---------------------ASITNNSYA-FDNSIGAVLSSYWTN-------------------------AVSPSPYLAA--
TF-------KVPDCASQPSIYDA--------MAVKTNATFRLGGD-----TACVNSI-----
GPSTSVCNGPLVPGMKYRVKYTLSEE----------SPQFPRTIVDQ-
TPWSDPVSTKKSPAASTINTWPGKRTGGMVVVTAVLSTLLFLLLAALLLVVIFKA--------------C
lamprey2		-----------------SQVVPTVVNPN--------
LLGAVTQTTVALQAPFCSALDAEVVALSSVSADLRLFVMATAQR-------------------NVSNEMIT---
SSSTIGLDKGYAGSG------------------------AGTSSWYLAL-GGR-------PLQNCTISTP------------
SLSTPTSYYRVGAD-----SKC----------SVAVTCNGPLNAGTIYWFKYIMGTVAT--------NGAIDR-SYLE-
SSWSKPIRLNKAGELNAIGVTPGPRSGGMVVVTVILVVLLFIAV------------DRARPALG------
Upk3B_human			----------------MVPYTPQITAWD--------LEGKVTATTFSLEQP--------
RCVFDGLASASDTVWLVVAFS-------------------NASRGFQNP-ETLADIPASPQLLTDGH------------------------
```

-----YMTLPLSP-------DQLPCGDPM-------------AGSGGAPVLRVGHD-----HGC----------
HQQPFCNAPLPGPGPYRVKFLLMDT----------RGS----PRAE-
TKWSDPITLHQGKTPGSIDTWPGRRSGSMIVITSILSSLAGLLLLAFLAASTMR----------------
Upk3B_mouse ----------------MIAYVPQITAWD--------LEGKITATTFSLEQP--------
RCVFDEHVSTKDTIWLVVAFS-------------------NASRDFQNP-QTAAKIPTFPQLLTDGH-----------------------
-----YMTLPLSL-------DQLPCEDLT-------------GGSGGVPVLRVGND-----FGC----------
YQRPYCNAPLPSQGPYSVKFLVMDA----------AGP----PKAE-
TKWSNPIYLHQGKNPNSIDTWPGRRSGCMIVITSILSALAGLLLLAFLAASTTR----------------
upk3B_chiken ----------------MLPYVPRVAPGA--------MPGKVTATTFVLERP--------
RCIFDPFANASDAVWLAVAFA-------------------DAPR-----------CPHDEGLPAARA-----------------------------
YMTLQMAA-------AAYGC------------------SAPGAAVLRVGGD-----TAC----------
HGRAPCNGPLPSPGPYRVKFLLMGC----------GG-----PKAE-
TKWSDPILLRRARSLSTIDPTPARRSSTAVVIAAILASLGAALAMAVLGAV-------------------
Upk3B_frog ----------------ITSYVPQLTLSP--------IVGTVTSTTFVLDKP--------QCVFGNTG---
NQVWLLVARS-------------------NVSANVVL-----TPPSMYSSFATK-------------------------GYYHVPFGTE------
-----SLYHC-------------------SNTAEYIRVGDT-----AQC---------NDNTNCNGPLPDPGPYRVKYLVMNN-------
---NA-----LVSQ-SLWSQQITLLTGKSSSQLDTWPGRRSGGMIVLTSILSVLMGILTLCLFAAFFVG---------------
-
PTPRB_human -----------------RPPPPPPHIRV------NEKDVLISKSSINFTVN--------
CSWFSDTNGAVKYFTVVVREA-------------------DGSDELKP-----EQQHPLPSYLEYRH----------------------
NASIRVYQTN---Y-------FASKC--------AENPN-----SNSKSFNIKLGAEMESLGGKC--D------
PTQQKFCDGPLKPHTAYRISIRAFTQLFDEDLKEFTKP-----LYSD-TFFSLPITTESEPLF-------------
GAIEGVSAGLFLIGMLVAVVALLIC-----------------
PTPRB_mouse -----------------RPPQPPPHIRV------NEKDVLISKSSINFTVN--------
CSWFSDTNGAVKYFAVVVREA-------------------DSMDELKP-----EQQHPLPSYLEYRH----------------------
NASIRVYQTN---Y-------FASKC--------AESPD-----SSSKSFNIKLGAEMDSLGGKC--D------
PSQQKFCDGPLKPHTAYRISIRAFTQLFDEDLKEFTKP-----LYSD-TFFSMPITTESEPLF-------------
GVIEGVSAGLFLIGMLVALVAFF-------------------
PTPRB_chiken -----------------RPPQPPPDIRV------NKKEVLITKSSINFTFN--------
CSWFSDTNGAVKYFTVVVREA-------------------DGSEGPKP-----DEQHPLPSYLEYKH----------------------
NDSIRIYQTN---Y-------FASRC--------AENPD-----SDYKSFDIKLGGEMENLGGKC--D------
PDQQKFCDGPLKPRTAYRISIRAFTQLFSEDPKELPQP-----LFAD-TFFSLPITTEAEPLF-------------
GVIEGVSAGLFLIVMLVAVTALFV------------------
PTPRB_frog -----------------RPPPPPLLIRV------NKKDTFISKSSIHFRFN--------
CSWFSDTNGAVKYFTVIVSEA-------------------DGNDNQRP-----EASLPLPSYADYKT----------------------
NKSTKIYQTS---Y-------FPSRC--------AENPD-----YNIQSYKIKLGTGMELLGGKC--D------
QNENKYCDGPLSPRTSYRISVRAFTQLFTEEMRTFPEP-----LYSD-TFFSLPITTEAGSLFFNKNITDLP----
LQIFPQTKAMILV----------------------------
PTPRB_fish -----------------RPPVPPVTVRV------NEHSAVITHFTIRFKFN--------
CSWFSDANGAIRYFTIIATES-------------------NDVDNGLP-----EQRHPLPSYLDYRQ----------------------
NHSIKAYQTG---Y-------FHSTC--------AEGSD-----GKVQVFEINLGAGMKHLGGAC--
KLDPESIQHGSHLCDGPLRSRTSYRLSVRAFTQLFDEENREFPHP-----LYTD-TYLSLPLLTQSAPRS-------------
GLTGGITAALFLITMVLALTALLI------------------

PTPRQ_human -----------------APARPKTKPTPIY---DATGKLLVTSTTITIRMP--------
ICYYSDDHGPIKNVQVLVTET-------------------GAQHDGNV-----------TKWYDAY-----------------------
FNKARPYFTN-EGF-------PNPPC--------TEGKTK----FSGNEEIYIIGAD-----NACMIP------
GNEDKICNGPLKPKKQYLFKFRATNI----------MG-----QFTD-SDYSDPVKTLGEGLSERTV---------
EIILSVTLCILSIILLGTAIFA--------------------
PTPRQ_mouse -----------------APARPKTKPIPIH---DATGKLLVTSTTITIRMP--------
ICYYNDDHGPIRNVQVLVAEA-------------------GAQQDGNV-----------TKWYDAY-----------------------
FNKARPYFTN-EGF-------PNPPC--------IEGKTK----FSGNEEIYVIGAD-----NACMIP------
GNEEKICNGPLKPKKQYLFKFRATNV----------MG-----QFTD-SEYSDPIKTLGEGLSERTV---------
EIILSVTLCILSIILLGTAIFAF-------------------
PTPRQ_chiken -----------------EPPRPKKKPAPVY---DTNGALLVTATTITIRMP--------
VCYYSDDHGPIKKIQVLVVEA-------------------GAQHDGNV-----------TKWHDAY-----------------------
FNRPRPYFTN-EGF-------PNPPC--------IEGKED----LSGKEEIYVIGAD-----TTCMIS------
GSQDKICNGPLKPRKQYLFKFRATNV----------KG-----QFTD-SDYSDPVKTLGEGRSGGSV---------
EVILAVTLCILSVVLLVAAVYAF-----AR------------
PTPRQ_fish -----------------APPKPKKTPRAAL---NSAGVIISTSKTITIEMP--------
ECFFTDDHGPIQKVQVIVSEP-------------------AVMDYGNL-----------SNWKSVF------------------------
LHPTAPYLTD-DGF-------LNPEC--------PKNSER----MSSSTKTYVIGED-----EGCL-S------
EDAETLCNGPLKPKTHYVFKFRATNI----------RG-----QFTD-SEYSDKVRTADDRLLTRDE---------
QIILGVLLSFFLALFLILIIYGS-------------------
CionaNocat_1 ------------PTIISGAPGPILSPTQA-------LFGASPTHAFQIENP----
CNRKSSLFSEIGGVITAIEVIVWQT-------------------GAASSKWE-------AATPAIWADAIN-----------------------
QNPIPPYVAG------------TIQCS-----TASGRKKR---ALTPNGSGYVIGAE------TC--T------
AANRVTCNGPLISGRQYNVAYRGVNG----------LG-----TPSDMTASNGPFSTSTQRGLEA-----------
GEIAAIVISSIVVLLLIICLVYYCV---KR------------
CionaNocat_2 -----------APTLIAGAAPPTAGPTTN-------ALGVSPTVAFQVENP----
CLRKAQLFSEIGGTINQIQLIVWQI-------------------QAPTPTTN----------ANVWANVIN-----------------------
QNPIVAYQAG------------TITCTG----TASGRKKR---ALTANNDGYVVGAD-----STC--T------
TTTVAVCNGPLPSGRQFNVSYIGVNG----------GG-----QTSGMTAPGGPFSTSTPTGLEA-----------
GEIAAIVISCIVVLLLIISLIYYCV---KR------------
Sponge -----------------PPTVPPNVTIGTPP--TGGTSDPTTATTIRIEVT--------
IPEELNANGPLTRIRILIRIF-------------------------------LSRNDTISTWYESQKFP---------------------
NSVAPPWQATQLPLN-----------------QGNRRKRQ----AGGETVAETIGTN-----NSC---------
GPNDIVCNGPLKPGTQYQFKYRVYNS--------DDDD-----SYVE-SQYSGPIRTGNPIAEENNT-------
GTTIVIAVVVVLLVIILLIAILVIVVIIVLKRRKRKAYSFAA--
Nematode_2 ----------------MAPPVPTVAPMI--------MKESVGSHNMIVRFP--------
TTMFDNRNGEIKQFAIIVSET-------------------TADESINR---WIESDNGTYTWQQVQR-----------------------
FDVWPSYVAK---------------------LQDIQKVKQ----DVDVSIFEELGED-----ETCL-E------
VRADRICNGPLRSASKYRVRIRLFTS----------PT-----LFTD-SPPSQVMTTGSATPA-------------
IPLLTVVAVLIVIAFVGIVGTIFLFFW-NRTKKAR-------
annelid --------------PIVMPNSPPPQAIT----------TAISHDKIRIILT---------
NPFLNTNGDVVAFSVFVTTDPNER------------F--------------MANSPLRTWADVKG----------------------
PSPMASYFAV---YKCANLFDGNDQCS-----SGPARKRRV--AQPRNTVEFTVGGD-----SSC--T------

TNADDYCNGPLDAESTYYVALVGYTE----------ND-----LYSS-GPSSEPIRTDTAPTNLL-----------
LIIIVVVVVLVLAIVAAIGVIVYM----RKRSSDNNE-----
ly_DPTP4e ---------------ILAPPVPEPSVTP--------LEVSRTSSTIEISFR--------
QGYFSNAHGMVRSYTIIIAED-------------------VGKNASGL---------EMPSWQDVQA-----------------------
YTVWLPYQAI-EPY--------NPFLT-------SNGSRK----SSLEAEHFTIGTA------NC--D------
KHQAGYCNGPLRAGTTYRIKIRAFTD----------ED-----KFTD-TVYSSPITTERSDT---------------
VIVAATVSAVLLVAMVLVVVYC-----QHRCQ---------
fly_PTP10D ---------------ILAPPRPATQVVP--------TEVYRSSSTIQIRFR--------
KNYFSDQNGQVRMYTIIVAED-------------------DAKNASGL---------EMPSWLDVQS-----------------------
YSVWLPYQAI-DPY--------YPF-------------------ENRSVEDFTIGTE------NC--D------
NHKIGYCNGPLKSGTTYRVKVRAFTG----------AD-----KFTD-TAYSFPIQTDQDNT--------------
SLIVAITVPLTIILVLLVTLLFY-----KRRR----------
fly_Ptp52F -----------------MPTIPSDELIKQMR--ANVEETSNPTKTAIVRLP--------
ADIMTSASGDIKWMALMISQK-------------------NCAGVPHL------KYDVSSDWPKVLSYQ-----EAGADGT----
-----GDCSLEYQTTEERW--------HPEP--------VQRQRRDGEVTSDEEIVFTIGLD------KC--S------
EVQKTYCNGPLLPDTDYNVVVRLFTA----------------------SGYSDAAVLNFKTKAAIKVT--------
LILVSVCSCLLLAFVLGLTVLWV-----RKRLA--------W
SeaUrchin1 E----------------PSPVPEDYPYE-----ANTVFSETSTTSFAVVLP--------
DDLFSHENGELLTFAVIITID---------------------DNDPTV------SSTELTYAARAEN--------------A------
YITAIEIPYP-----Y--------SPSF-------GSNRRRR----ATDPPGTVVIGDG------TC--A------
GSQNEYCNGDLDDNTEYYYAFRAYNG----------MG-----NVTS-STFG-PVKTVKDNTA-------------
GIAAGVSVSLIIIIVAVVVVVVFL----KRRQPKEPSPRPS-
SeaUrchin3 -----------------APPTPPADRVV--------RLVSHTGTTMSVSFD--------
SSIFSDVYGPVTRYALLVAQS-------------------SPDTIPST------PSDPAINWQ--------------------------
VGSGQPYQTTLLTY--------APFT------------------NGNTRGFYTVGAN-----NSC--T------
QDSVGYCNGPVVPLTDYRFALRAYGS----------DG-----KFTD-SAWSPLYNSGINTT--------------
WYAAAVVSIIIITLVLLLVMLLI-----SRQC----------
SeaUrchin4 -----------------APPSPPRGATP---------SLVSRGSTITITFF---------
NLFDTAFGRIVRFAIIVQER-----------------VDGGTVVITA----KRQTTSSLTWAEARR-----------------------
TRPVPSYQTTPDDY--------NPFA------------------DGAGVTTFRVGSQ------SC--D--P---
DDLTAYCNGALYPGTSYRFAIRAYGV----------DG-----KFVD-TEFSSPFRTDPDRGI-------------
FVIPAIVLAIMVVIILIIFMAMGFGC----------------
facornWorm -----------------PPPKPTASRIT-----ANVRFVETTAYTLRIHLS--------
NNLFDDRFGIIVAYLVIIAED-------------------GGEYSPLP--------EVLPSYDEVID-----------------------
SSPWPPYQTS-QPF--------NPFET-----LSIGRRRR----NTLQTVNYIIGAK-----ENC---------
DVSLVYCNGALRPVTDYRYVIRGYNE----------LG-----NYTD-TDWSLPQKTDLDPF--------------
WILYGVIAGLILIGIIILICICYCCCC-RRRRSSTQDSDK--
acorworm1 -----------------PPPPPRNNIVPS----SSESDIEVSQTTITVLFT--------
DDYFNDSNGPLLNFTVIVTEDGTEEVDKHLNGEIPRVYCKDCENVPGI------EEGEVKTWAEVQN-------------
------------TPARYQID-YPY-------DYPDL---------SRRKR-----DTTGIEITIGSD-----DSC--S------
TKDEGFCNGPLKDGTSYRYHFRAYTD----------VG------YSD-TVLSGPIRTSTNMSWL------------
WLLLGVLLGVLIAVAIILAIIYY-----KRRYYEETT-----
ciona -----------------APPVPDTWPEVMNKIDDTSTMASVTSSTITVKKP---------
CMFSNENGPIASLSVIIAED-------------------GGNIEAEP-----------TYWSQAFP-----------------------

LQPSPPYKVL-VSEN------PTDYC--------NTRKRR-----SINEKGFVIGTS------DC--P------
SELNTHCNGPLKSSTDYRVKFRAETS----------NG-----LKTD-TEFSEIIRTSPSFLATHM----------
TLLIGIGAAIGLFTILVVISAVFLRLVMQYSIQYGFLNNV-F
Ciona2 -----------------APPVPALLPEVIIQQ----DKAIVTSSSVIVKKP---------
CVFSDDNGPLKSLSVIVAQE-------------------GATLDAEP-----------TYWAKAYS----------------------
EEPSPPYKVI-VTEE------PQSYC--------NSRNKR----STQTNNGFVIGTS------DC--S------
RLSTSQCNGPLKSNTEYRFKYRAEAN----------NG-----LMTD-TEYSEVFRTNPSFIEAHM----------
TLLVSIGAALGIFIILLTISVALLR-----------------
PTPRF_human ----------HLVSIRTAPDLLPHKPLP--------ASAYIEDGRFDLSMP--------H---
VQDPSLVRWFYIVVVPI----------------------------
DRVGGSMLTPRWSTPEELELDELLEAIEQGGEEQRRRRRQAERLKPYVAA--------------------------------
QLDVLPETFTLGDK-----------------KNYRGFYNRPLSPDLSYQCFVLASLKEP------MDQK-----RYAS-
SPYSDEIVVQVTPAQQQEE--PEM----LWVTGPVLAVILIILIVIAILLF-----KR------------
PTPRF_mouse ----------HLVSIRTAPDLLPQKPLP--------ASAFIEDGRFSLSMP--------Q---
VQDPSLVRWFYIVVVPI----------------------------
DRVGGNLLAPRWNTPEELELDELLEAIEQGEEKQRRRRRQAERLKPYVAA--------------------------------
QVDVLPDTFTLGDK-----------------KSYRGFYNRPLSPDLSYQCFVLASLKEP------MDQK-----RYAS-
SPYSDEIVVQVTPAQQQEE--PEM----LWVTGPVLAVILIILIVIAILLF-----KR------------
PTPRF_chiken ---------QHLVSIRTAPDVLQSKPIA--------TNKYIQEGKFTLTLP--------K---
VQTTVPVRWYYIVVVPA----------------------------EQ-SPSSPTARWRTPDEMELDQLLEAISQGSQ-
SRRQRRQADRLKPYIAA--------------------------------QVDVLPETFTLGDE-----------------
KNYKGFYNKPLSQDLSYRCFVLASLE-D------GDTK-----RYAA-SPYSDEIVMELASAKQQDE--PEM----
LWVMGPVLAVILIIIIVIAILLF-----KR------------
PTPRF_fish ---------QQVVSIRTAPDLLKSKPVI--------YRGQEEDGKLTISLN--------
RVCISDRSSCLKLIITCFFIT-----------------------------------ESYQWTDGVSQPYNKGLKSSKEGAC-----
PRVKESRKPYSAPLSSL----------------------------------QLVTAGPE-----NTFP-SL---
SRRNYLKFCNNKVKTNHFKSLEILKKNL---PYFYTLFQK-----TFAA-SPYSDPITVEVVNRMPRQPEEPEM----
LWVMGPVLAVILIIIIVIAILLF-----K-------------
PTPRF_monosiga VILRGPGRVAERRTLSARPEAPPAVTSAVL------PDGYSSQSAFAVSWVAP----------
STYSGPIRRLSLVVEPQ-------------------GSSTHAVASLDCASSSCDFGTWSEAQA----------------------
ASSPMAYIAYSQTF-------TESEG-----------------LGDLSRSSFVIGAER----EKV--D------
ADGDGYVNGPLKAGATYTFRLLTCTQ----------NGND---ELCQ-AASLNPTTALASTAAAPAD-------
NTGIIVGVVIVIIVLIVAVLVFVMW-----RRRQNKAAAPDEDF

upk3A_human GGGGGG
upk3A_mouse GGGGGG
Upk3A_chiken GGGGGG
upk3A_frog GGGGGG
UPK3A_fish GGGGGG
lamprey1 GGGGGG
lamprey2 GGGGGG
Upk3B_human GGGGGG
Upk3B_mouse GGGGGG
upk3B_chiken GGGGGG

Upk3B_frog	GGGGGG
PTPRB_human	GGGGGG
PTPRB_mouse	GGGGGG
PTPRB_chiken	GGGGGG
PTPRB_frog	GGGGGG
PTPRB_fish	GGGGGG
PTPRQ_human	GGGGGG
PTPRQ_mouse	GGGGGG
PTPRQ_chiken	GGGGGG
PTPRQ_fish	GGGGGG
CionaNocat_1	GGGTGG
CionaNocat_2	GGGTGG
Sponge	TGTTGG
Nematode_2	TGTTTG
annelid	TGTTGG
ly_DPTP4e	TGTTTT
fly_PTP10D	TGTTTG
fly_Ptp52F	TGTTGT
SeaUrchin1	TGGGGG
SeaUrchin3	TGGGGG
SeaUrchin4	TGGGGG
facornWorm	TGGGGG
acorworm1	TGGGGG
ciona	GGGGGT
Ciona2	GGGGGT
PTPRF_human	TTTTTT
PTPRF_mouse	TTTTTT
PTPRF_chiken	TTTTTT
PTPRF_fish	TTTTTT
PTPRF_monosiga	TTTTTT

;

END;

[DNA sequence matrix follows; to use copy and paste from this file to a new file and remove brackets.
#NEXUS

BEGIN DATA;
DIMENSIONS NTAX=40 NCHAR=949;
FORMAT DATATYPE = Protein GAP = - MISSING = ? interleave;
MATRIX

upk3A_human --
gtgaacctgcagccccaactggccagtgtgactttcgccac--------------
caacaaccccacacttaccactgtggccttggaaaagcctct-------------------------
ctgcatgtttgacagcaaagaggccctcactggcacccacgaggtctacctgtatgtcctggtcgactcagccatttccaggaatgcctca
gtgcaagacagcaccaacaccccactgggctcaacgttcctac-----------------------------
aaacagagggtgggaggacaggtccctacaaagctgtggcctttg---------------------------acctgatcccctgcagt-----------
-----------------gacctgcccagcctggatgccatt--------------------------
ggggatgtgtccaaggcctcacagatcctgaatgcctacctggtcagggtgggtgccaacgggacctgcc------
tgtgggatcccaacttccagggcctctgtaacgcacccctgtcggcagccacggagtacaggttcaagtatgtcctggtcaatatgtccac
gggcttgg-----------------------tagaggaccaga---ccctgtggtcagaccccatccgcacca-
accagctcaccccatactcgacgatcgacacgtggccaggccggcggagcggaggcat-
gatcgtcatcacttccatcctgggctccctgcccttctttctacttgtgggttttgct-ggcgccattgccctcagcctc--------------------
----
upk3A_mouse --
gtgaacctccagccccaactggccagtgtgacctttgccac--------------
caacaaccctaccctcaccaccgtggccttggagaagcctct-------------------------
gtgcatgtttgatagctcagagccactcagcggctcttacgaggtttacctctatgctatggtcgactcagccatgtccaggaatgtgtctg
tacaggacagcgctggcgtcccactgagcaccactttccggc-----------------------------
aaacccagggtgggaggtcaggcccctataaagctgcggcctttg---------------------------acctgaccccttgtggt------------
----------------gacttgcccagcctggatgctgtt--------------------------
ggagatgtgacccaggcctcagagatcctgaacgcatacctagtcagggtgggcaacaacgggacctgtt------
tttgggaccccaacttccagggcctctgcaacccacccctgacagcggccactgagtacagattcaagtatgtcctggtcaacatgtccac
aggcttgg-----------------------tgcaggaccaga---cactatggtcagatcccatctggacca-
accggcccatcccctactcggccatcgacacgtggcccggccggcggagtggaggcat-
gattgtcatcacgtccattctgggctccctgcccttcttcctgctcgtgggtttcgct-ggagccatcatcctcagcttt-------------------
----
Upk3A_chiken --
cagagcatgaaacctcagcttgcagcccccgagcttgcaac--------------
aaacaatcccactctcactacagttgccctagagaaaccttt-------------------------
ctgtatgtttgatagctcactacatccaaacaaatcttatgccatctacttgtatgtgatgaaaagttcagccaacacaataagctccgtgg
tgactgacagcagcagcaagccactggacagcacgttccagc-----------------------------
aaacacatgggggacatcttggaccttacaaggctgcctcattcg---------------------------atgtacccaactgtgtg------------
----------------tcaccccccaggcttgctgatgca--------------------------
ggagacatcaacaaagtttccgatgtcctgaaacaatacctcttcagagttggggatgatgggacttgtt------
tgtatgacccaaacttcctagatgtctgcaacccacctcttgcaccagacacaacatacaggtttaaatacgtgttggtcgataacactga
aggtatcg-----------------------tgaaagaccaaa---ctctttggtctgatccaatcaaaacca-
gaaaagctaaacttcccatgaaaattgatatctggcctggtcgaaggagtggaagcat-
gattgtcattacatcaattctaagtgtgtcagtgttccttctgcttgctggcttgctt-gcttctgtgttttctgctctc----------------------
----
upk3A_frog --gga------
gcagacatgccgttgctggccaactcggatttctttag--------------
cctcaatcccacccagactacgattgcgctggagcagccgat-------------------------ctgcatgtttaaatcggccg-------------

----
ttaacgtctatctaatcgggatcgtggcaggtgccccaaacaccccgctttatgacggcaataagaaggttaatgcctcaacctacagcg-
-----------------------------gaacccagggggggcaagacgggaccctatatcgtggccaaacttc----------------------------
ccaaccaacaatgtatc-----------------------------aacatacaggccctgagcaatatg---------------------------
gccgaccccacgcaggtgcagtccatccttagcaaatacgtcgtgagagtgggggccgacgtgacctgct------
taaccaatcccaactttgtggggtactgcaacgccccccctgcaggggaacactcaatacagtttcaagtatctgtttacagacagtgggg
atat---------------------------tgtgcagtccgaga---ccagctggtccctgggcatcactaccg-
tcaatggcaaagcttctagtaccatagacacctggccgggcaggaggagtggtggcat-
gattgtcctgacctccattctcagcactctgatgttcttcgttttcattgcctacgtc-attggctttgcttactccata--------------------
----
UPK3A_fish --
gaggctctgaaggtgaagcccgaggctgtgtctcccaggctgctgcg---------------
gtttaacccgacacagagcacggtgtccctcgcgaagccgct--------------------------
ttgcgttttcgacagcgtgaagccaacgaatgaaat---
gatggtagatgtatacgttgtccacagcctctcggccactctgacctttgagactgggaa------------------------aacgtacaagg---
---------------------------agacgaatggagggacagagactccttataaggctacatccttcg----------------------------
ggatcccaaactgcacc-----------------------------tctccaccaaaccctgcagacctg---------------------------
tctgttccccagagaatcgataagactttagatgagtacttggtccgtattggcagcaacccgacatgcg------
tgggggaaccggaggctgaagccttttgtaatgcgccgctctccgatggcacttcgtacaggtttaaatatttgcttgtaaatgggactac
aac-------------------------------tatagcagaaa---ccgaatggtcagagtcaatcctaacaa-
gaaaggctctgagccctgatgaaattgacacctggattgggaagagaagtggaggcgg-
gatagttgtcactgtcatcctgagtctcctgctgtttctgctgcttggggctgccatt-ttcatgggagtactggatgtg--------------------
----
lamprey1 ------------agc--
ggcaccaagcctcaactcgtgagctcgacggccgttcc---------------
gtacaacccgacggagacaacgatcgtgtggtccaagcccttt--------------------------
ctgcgtgttccaaaagcccgttcccaccactc---------------
agtacgtcgtggacgtctacgcgtccatcaccaacaacagttatgcattcgacaactcaatcggcgcggtgctctcctcctact----------
-----------------------ggactaacgcggtgtcgcccagtccgtacctggcagccacattca----------------------------
aagttccggactgtgca-----------------------------agtcaacccagcatttacgacgccatggctgtca----------------------
-------------------aaacgaacgcgacattccgtctgggcggcgacacggcctgcg---
tgaacagcatagggcccagtacgtccgtatgcaacggaccgctcgtacccggaatgaagtacagagtgaaatatacactttcggaagaa
tctcccca---------------atttcccagaactattgtggatcaaa---ctccctggtcagaccctgtctccacaa-
agaaatcgccagcggccagcaccatcaacacgtggcccggcaaacgcacgggtgggat-
ggtggtggttaccgccgtcctcagcacgctgctcttcctgctgctcgccgcactcctc-ctggtggtcatcttcaaggcg------------------
-----------------------------tgc
lamprey2 --
tcacaggtggtgcccaccgtggtgaaccccaacctgctggg------------------
agccgtgacgcagacgacggtagcgctccaggcgcccttt--------------------------
ctgcagcgccctggatgcggaggtggtggctctcag---
ctcggtctccgccgacctgcggctcttcgtgatggccacggcgcaacgcaacgtgagtaacgagatgatcacgagctcgtcgacgatt---
---------------------------------------gggctcgacaagggatacgcgggctcgggtgcggggaccagcagctgg-------------
tacttggctctgggaggccgcccc------------------------ctacaaaactgcaccatctccacgccgtctctgtcga------------------
-----------------------------cgcccaccagctactaccgcgtgggcgccgactccaagtgca------------------

gcgtggcggtcacgtgcaacgggccactcaatgccggcaccatctactggttcaagtacatcatgggaacggtggctaccaacggggcc
at-----------cgaccgctcttacctcgagt---cttcctggtcgaaacccatccggctga-
acaaagccggtgaactcaacgcgattggcgtgaccccctgggccgcg?tcgggcggcat-
ggtggtggtgacagtcatcctggtcgtgctcctcttcatcgcggttgatcgggcccgg-cctgctcttggt------------------------------
----
Upk3B_human --
ctggtgccctacacaccacagataacagcttgggacctggaagg-----------------
gaaggtcacagccaccaccttctccctggagcagccgcg-------------------------ctgtgtcttcgatgggcttg------------------
-----ccagcgccagcgataccgtctggctcgtggtggccttcagcaatgcctccaggggcttccagaacccggagacac----------------
----------------------------------tggctgacattccggcctccccacagctgctga--------------------------------
ccgatggccactacatg----------------------------acgctgcccctgtctccggaccagctgccctg-----------------
tggcgaccccatggcgggcagcg------gaggcgcccccgtgctgcgggtgggccatgaccacggctgcc-----------------
accagcagcccttctgcaacgcgcccctccctggccctggaccctatcgggtgaagttcctcctgatggacaccaggggctc------------
--------------acccagggctgaga---ccaagtggtcagaccccatcactctcc-
accaagggaagacccccggatccatcgacacctggccagggcggcgaagtggcagcat-
gatcgtcattacctccatcctctcttctctggccggcctcctactcttggccttcttg-gcagcctctaccatgcgc-----------------------
----
Upk3B_mouse --
ctgattgcctacgtgccgcagataacagcctgggacctggaagg-----------------
gaagatcacagccactacattctctctggagcagcctcg-------------------------gtgcgtctttgatgagcatg------------------
-----tctcaactaaggacaccatctggctagtggtggctttcagcaatgcctccagggactttcagaacccacagactg-----------------
----------------------------------ctgctaagatcccgaccttcccacagctgctga--------------------------------
ctgacggccactatatg----------------------------acattacccctgtccctggatcagctgccatg-----------------
tgaggacctgaccggtggcagtg------gaggtgtccccgtgcttcgggtgggcaatgattttggctgtt-----------------
accagcgaccctattgcaacgcccccctccccagccagggcccttacagtgtgaagttccttgtaatggatgccgccggccc------------
--------------acccaaggctgaga---cgaagtggtccaaccccatttatctcc-
accaaggaaagaatcccaactccattgacacatggcctggccgacggagcggctgtat-
gatcgtcataacttccatcctctctgccctggccggcctcttgctcctggctttcctg-gcagcttccactacgcgt-----------------------
----
upk3B_chiken --
ctgctgccctacgtgccccgcgtggcccccggtgccatgccggg-----------------
gaaggtgacggccaccaccttcgtgctggagagaccccg-------------------------ctgcatcttcgacccctttg------------------
-----ccaacgcctccgatgccgtctggctggcggtggcttttgccgacgcgccgaggtgcccccatgacgaggggctgc----------------
---ctgccgcccgcgc--------------------------
ctacatgacactgcaga----------------------------tggcggcagccgcctacggatgctcagctc------------------------------
------------------ccggtgcagccgtgctgcgggtcggcggggacacggcgtgcc-----------------
acggccgggcaccctgcaatgggccgctgccctccccggggccctacagggtgaagttcctgctgatgggctgcggtggccc-----------
-------------------caaagcggaga---cgaagtggtccgaccccatcctcctgc-
ggagagctcgcagcctgagcaccatcgaccccacacccgcacgtcgcagcagcaccgc-
ggtcgtcatcgccgccatcctggccagcctgggtgccgcgctggccatggccgtgctg-ggagctgtg--------------------------------
----
Upk3B_frog --
atcacttcatatgttccacaattgaccctctcgcctatcgtggg-----------------
cacagtaacaagtacaacatttgtactagacaaaccaca-------------------------atgcgtatttggtaacacag-----------------

----------------ggaatcaggtttggttgcttgttgctagaagtaacgtgtcagcgaatg--
-------------------------tggtgctcactccaccctccatgtattcatcatttgccacta--------------------
aaggatactaccatgtt---------------------ccctttggcactgaatctttataccactgct--
---------------ctaacacagctgaatatatccgagttggagatactgcgcagt---------------
gtaatgataatacaaactgcaatggtcccttacctgatcctgggccttaccgggtgaaatatcttgtgatgaacaataatgctct---------
---------------------agtctcacaat---cactttggtctcaacaaataactttgc-
tgacagggaaaagttcatctcaactcgatacttggcctgggaggagaagtggaggaat-
gattgttctaaccagcattctatcagttcttatgggaattttgacactttgcctattt-gctgctttctttgttgga--------------------------
----
PTPRB_human --
cgcccccctcctccacccccacacattcgtgtgaatgaaaagga---------------
tgtgctaattagcaagtcttccatcaactttactgtcaactg--------------------------cagctggttcagcgacacca----------------
----
atggagctgtgaaatacttcacagtggtggtgagagaggctgatggcagtgatgagctgaagccagaacagcagcaccctctcc-------
---cttcctacctggagtacaggcacaatgc---------------------------
ctccattcgggtgtatc-----------------------------agactaattattttgccagcaaatgtgccgaaaat----------
cctaacagcaactccaagagttttaacattaagcttggagcagagatggagagcctaggtggaa------------------
aatgcgatcccactcagcaaaaattctgtgatggaccactgaagccacacactgcctacagaatcagcattcgagcttttacacagctctt
tgatgaggacctgaaggaattcacaaagccactctattcagaca---catttttttctttacccatcactactg----------------------
aatcagagcccttgtttgg-------------------agctattgaaggtgtgagtgctggtctgtttttaattggcatgctagtggctgttgtt----
---gccttattgatctgc------------
PTPRB_mouse --
cgcccgcctcaaccgcctccacacatccgtgtgaatgaaaagga---------------
tgtgctaatcagcaaatcttccatcaactttactgtcaactg--------------------------cagctggttcagcgacacca---------------
----
acggagcggttaaatactttgctgtggtggtgagagaggccgacagcatggatgagttgaagccagaacagcagcaccctctcc-------
---cttcctacctggagtacagacacaacgc------------------------
ctccatccgagtctacc-----------------------------agaccaattattttgccagcaaatgtgctgaaagt----------
cccgacagcagttctaaaagtttcaacattaagcttggagcagagatggacagcctcggtggca------------------
aatgtgatcccagtcagcagaaattctgtgatggaccgctgaagccacacaccgcctacagaatcagcatccgggcttttacacagctat
ttgacgaggacttgaaagagttcaccaaacctctctactcggata---cgttcttctctatgcccatcaccacag---------------------
agtcagagcccttgtttgg-------------------agttattgaaggtgtgagtgctggcctgtttctaattggcatgctggtggcccttgtt----
---gccttcttc---------------
PTPRB_chiken ---
cgtccccctcagccacctccagacatacgagtaaacaaaaaaga---------------
ggtgctgatcaccaaatcctccattaactttacttttaactg--------------------------cagctggtttagtgatacta----------------
----
atggagctgtgaagtacttcactgtggttgtcagggaggctgatggtagtgaaggaccaaagcctgatgagcaacacccgttac--------
--cttcctacctggagtacaaacacaatga------------------------
ctccatacgcatctacc-----------------------------agacaaattatttcgccagtagatgtgctgaaaac----------
cctgacagtgactataaaagctttgacattaagcttggaggagaaatggaaaatctgggaggaa------------------
agtgtgatccggatcagcaaaaattctgcgatggacccctgaagcctcggactgcttataggatcagcatacgggcttttacccagctctt
cagtgaagacccaaaggaacttcctcaaccgctctttgcagaca---ccttcttctccttaccaatcactacag----------------------

aggcagagcctctctttgg-------------------agttattgaaggtgtgagtgctggtttgtttctgatcgtgatgttagtggctgttact----
---gctttatttgtc---------------
PTPRB_frog --
cgccctcctccgccacctcttttaatcagagtgaataaaaaaga---------------
tacattcattagcaagtcatcaatccacttcagatttaattg--------------------------cagttggttcagtgacacca----------------
----
atggagctgtcaaatatttcactgtgattgtttcggaagcagatggcaatgacaaccagaggcctgaggcaagccttcctttgc----------
--catcatatgcagattataaaacaaataa-------------------------
gtccactaaaatttacc-----------------------------agactagctactttccaagtcgatgtgcagagaac----------
cctgactacaatatccagagttacaaaattaagctgggtacaggaatggaacttttgggtggca------------------
aatgtgatcagaatgaaaataaatactgtgatggaccactgagtccaaggacatcctacaggataagtgtcagagcttttacccaactat
ttactgaagaaatgaggacattccctgagccactgtatagtgaca---ctttcttctccttgccaatcacaacgg---------------------
aagcaggttctttattt----------------------tttaacaaaaacataacagatttgcctttacagattttcccacagacaaaagccatg---
--atcctggta-------------------
PTPRB_fish --
aggccccctgtcccaccagtgaccgtacgggtcaatgagcattc---------------
agcggtcatcacccatttcaccatccggtttaagttcaactg--------------------------cagctggttcagtgatgcta----------------
----
acggagccattcgctacttcacaataattgccacggagtcaaatgatgttgataatggcttgccggaacagagacatcctctgc---------
--catcttatctggactatagacagaacca------------------------
ctccatcaaagcctacc-----------------------------agacgggctactttcacagcacgtgtgccgagggg----------
tccgacggcaaggttcaggttttcgagataaacctgggagcaggaatgaaacatttgggcggagcttgcaaattggatccagaatctatc
cagcacggatcacatctttgtgacgggccccttagatccaggacatcgtatcgattaagcgtccgtgccttcactcaactgtttgatgaag
agaacagagaatttccacacccgctttatacagaca---cctatctctcgctccctttattaactc---------------------
aatcagcaccacgaagcgg-------------------
cctgacaggagggatcacggctgccctgtttctcatcactatggtgctcgccctgaca---
gccttactgatt---------------
PTPRQ_human ---
gctccagcacgaccaaaaaccaaaccaaccccctatttatgatgccacaggaaa---------------
actgcttgtgacttcaacaacaattacaatcagaatgccaat--------------------------atgttactacagtgatgatc---------------
------------atggaccaataaaaaatgtacaagtgcttgtgacagaaacaggagctcagcatgatggaaatgtaa--------------------
---caaagtggtatgatgcatattttaataa------------------------
agcaaggccatatttta-----------------------------caaatgaaggctttcctaaccctccatgtaca-------------------
gaaggaaagacaaagtttagtg------gcaatgaagaaatctacatcataggtgctgataatgcatgca------
tgattcctggcaatgaagacaaaatttgcaatggaccactgaaaccaaaaaagcaatacttatttaaatttagagctacaaatattatgg
gaca-----------------------------atttactgact---ctgattattctgaccctgttaagactt----------------------
taggggaaggactttcagaaagaaccgtaga-------
gatcattctttccgtcactttgtgtatcctttcaataattctccttggaacagctatt-tttgca--
----
PTPRQ_mouse ---
gctccagcacgaccaaaaaccaagccaattcctattcatgatgccacaggaaa---------------
actgcttgtgacgtcaacaacaattacaatcagaatgccaat--------------------------ctgctactacaatgatgacc--------------
-------------acgggccaatcagaaatgtgcaagtccttgtggcagaagcaggagctcagcaagacggaaatgtga------------------
---caaagtggtatgatgcatattttaacaa------------------------

agcaaggccatatttta---------------------------caaacgaaggattccctaatcccccgtgtata------------------
gagggaaagactaagttcagcg------gtaatgaagaaatctatgtgataggtgctgataatgcctgca------
tgatccctggaaatgaggagaaaatttgcaatggacctctgaaacccaaaaagcagtatttgtttaaatttagagccacaaatgtcatgg
gaca----------------------------atttactgact---ctgagtactctgaccctattaaaactt--------------------
taggtgaaggactttcagaaagaaccgtgga-------
gatcatactgtcagtcactttgtgtatcctctcaatcatcctccttggaacagctatt-tttgcattt----------------------------------
----
PTPRQ_chiken ---------------------------------------
gaaccaccacggcctaaaaagaaaccagcacctgtttatgataccaatggggc--------------
cttacttgtcacagcaacaacaattacaatcagaatgccagt------------------------atgttattacagcgatgatc--------------
-----------atggacctatcaagaagatacaagttcttgttgtggaagctggagctcagcatgatggaaacgtta--------------------
--ctaagtggcatgatgcatacttcaacag----------------------
accaaggccatatttta---------------------------caaatgaaggctttccaaacccaccatgtata------------------
gaaggaaaggaagatctgagtg------gtaaagaagaaatatatgttataggtgctgatactacatgta------
tgatatcaggcagtcaagacaaaatatgtaatggcccacttaaaccaagaaagcagtacctattcaagttcagagcaactaatgttaaa
ggaca----------------------------gtttacagatt---ctgactattctgaccctgtcaaaactt--------------------
taggtgaagggcggtcaggaggatctgtgga-------
agttatccttgcagttactttgtgtatactttcagttgttcttctggtggctgcggtc-tatgcttttgcaa------------------------------
---------------------ga
PTPRQ_fish ---------------------------------------
gctcctcccaaaccgaagaagacgccgcgggcagctctgaacagtgctggtgt--------------
catcatctcaacctccaagaccatcaccatcgaaatgcctga------------------------atgtttcttcactgatgacc--------------
-----------atggaccaattcagaaagtccaggtcatagtttctgaacccgcagtgatggactacggtaatctat---------------------
---ccaactggaagagtgttttcctccaccc----------------------
cactgctccatacctga---------------------------cagacgatggcttcctgaacccagagtgccct------------------
aaaaattctgagcgcatgagct------caagcactaagacgtatgttataggagaagacgagggctgcc------tg---
tctgaagatgcagagacgctttgtaacggacctctgaaacccaaaacacattatgtgttcaaatttcgagcgacaaacatacgtggcca-
---------------------------gttcacagact---ctgagtactcagacaaagtcagaactg--------------------
cagatgaccgcttgctgaccagagatgagca-------
gatcatccttggagtcttgctgtcatttttctggcgttgtttttaattctgattata-tatggatct------------------------------------
----
CionaNocat_1 ---------------------------------------
ccaactattatatctggtgcacccggcccaatacttagcccaacccaagcactctt-----------
tggggcttccccaacccatgctttccaaatagagaacccttgtaac-------------agaaagtcgagtttgttcagtgagattg---------
----------------gtggagtgataaccgcgatagaagtcatcgtgtggcagactggagctgcaagcagta--------------------------
------------------------------agtgggaggctgccacacctgcaatatgggctgatgccatcaaccaaaa----------------------
cccaattcctccatacg---------------------------ttgctggcactatacaatgttcta---ctgcatct------------
ggtcgtaagaagagagctttaacac------ccaacggatctggatatgtgattggtgcggaaa-------------
cttgcactgcggcaaacagagttacatgcaatggaccacttatatctggtcgacaatataatgttgcttatcgtggggttaacggtctcgg
cac----------------------------accctcagatatgactgcctccaacggccccttttcaacaa--------------------------
gtacgcaacgaggtttggaggctgggga----aattgcagcgattgttatctcaagcattgtggtgttacttcttataatttgccttgtc------
-------------------------------------tactactgtgtgaaa--------cgg
CionaNocat_2 ------------------------------------
gctcctacattaattgctggagctgccccccctactgctggccccacaacaaacgctct-----------

tggagtttcacccacagttgccttccaagttgaaaacccatgcctc-------------agaaaagcgcagttattttcagaaatag---------
----------------gcggcactattaatcaaatccaacttattgtttggcaaattcaagcacctact-----------------------------------
------------------------------ccaacaactaacgccaacgtttgggcaaatgttatcaatcagaa-----------------------
tcctattgttgcatacc----------------------------aagctggcactataacatgcactggaacagcgagt------------
ggtagaaagaagagagctttaactg------ctaacaatgatggctatgttgttggtgctgattcca-----------
catgtacaacaacaactgtagccgtttgtaatggcccgcttcctagtggtcgtcagttcaatgtcagctacataggagttaatggtggtggt
ca-----------------------------aacctcagggatgactgcaccaggtggtcccttttcaacaa--------------------------
gcaccccaactggattggaagctggcga----aattgctgctattgtcatttcttgcattgtagttttgttgctaatcatatcacttatt-------
-------------------------------------tactactgcgtgaaa--------agg
Sponge --
cctccaacagtaccacctaatgttactataggaacaccccctac---
----------------tggtggcaccagtgatcccacaa-----------------------
ctgccacaacgataagaatagaagtcacaataccagaggaacttaatgctaatggtccactgacgaggattcgtatattgatacgtattt-
----------------------ttctatctcgaaatgatacaatatccacctggtatgagagtcaaaaattc-------------------------
ccaaattctgtggcacc-------------------------accatggcaggcgactcaactaccattaaatcaagga-------------
aataggaggaagagacaggctggcg------gtgagactgtagctgagaccataggtactaaca--------------
actcgtgtggtcctaatgacatcgtttgtaacggcccattaaaaccagggacccagtaccagttcaagtacagggtgtataatagtgatg
atgatga-----------------------ttcctatgtggaga---gtcagtactctggccccattagaacag----
gtaatccaatagctgaggaaaataatactggtacaac------------------
aatagtgattgcagtggttgtggttctattggtcattatattattgattgctattctt-
gttattgttgttattattgtccttaagagaaggaaaagaaaagcatactcttttgctgca-----------
Nematode_2 -------------------------------------
atggcaccaccagtgcctacagtagctccaatgattatgaaggaaagtgttggaagtca----------------------------
taatatgattgtgagatttccaac-------------------------aacaatgttcgataatagaa--------------------------
acggagaaattaaacaatttgctataattgtctcggaaaccacagctgacgaatctataaatagatggatagagagc-----------------
-----------------------------gacaacggaacttatacatggcaacaagtacaacgatttga-----------------------
tgtttggccatcttacgtggcaaaactt--
caagatattcagaaagtgaaac------
aagatgtagatgtgtcgattttcgaagaattaggggaggatgagacttgtctagaagtgagagccgatcgtatatgtaacggaccacttc
gttccgcatcgaaatatcgtgtcagaatccgcttgttcacgtctccgacttt-----------------------------gttcacagatt---
cacctcccagccaagtgatgaccaccg---------------------------gctcagcaactcc-------
agcaatcccattgctaacagttgtcgctgttctgattgtgatcgcattcgtcgggatcgtcggaaccatattcctcttcttctggaatcgaac
aaagaaagcgaga-----------------------------------
annelid --
cccattgtaatgcccaactcgccaccaccacaagctattacaactgcgatttc--------------
acacgacaaaatccgaatcattctgac---------------------------------------aaatccgtttttgaatacta--------------------
------atggggatgtcgtcgcgttttcggtttttgtgaccaccgatccgaatgaacgttttatggctaatagtccgc----------------------
--------------------------------------tacgaacatgggcagacgtcaagggtccatc-----------------------
tccgatggcttcgtatttcgctgtctata----------------
aatgcgccaacttatttgatggcaatgatcagtgctcaagcggcccggcgaggaaacgccgcgtagctcaaccaa----
gaaacaccgttgagttcactgtgggtggcgactcaa-----------
gctgcacaacaaatgctgatgactactgcaatggacccttggatgctgagtctacgtactatgttgcacttgttggttataccgagaatgat
ct----------------------------atattcaagtg---gtccctcttccgaacccattcgcactg---------------------------

acactgcgcctac-------gaacttgttgctgattataattgtggtcgtggttgttctggtgttggccatcgtggctgccattggtgtc--
attgtttacatgagaaagcgatcttcagacaataatgag------------------------------
ly_DPTP4e ---
atactagcacctccggttccggagccaagtgtaacaccactgga---------------
agtgagcaggaccagtagcaccatcgagattagtttccgtca-------------------------gggttacttctccaacgctc-------------
------------atggcatggtaagatcctatacgataatcatagccgaggatgtgggcaaaaatgcctccggactggagatgc-----------
--ccagctggcaggatgtgcaggcatatac-----------------------
cgtgtggctgccttatc----------------------------aagccatagagccatacaatccattcctgaccagc----------------
aatggcagcagaaagagcagcc------tggaggcagagcattttacgataggaacggcga---------------
actgcgataaacatcaggcgggctactgcaatggtccgctgcgggctggaaccacctataggattaagattcgtgcctttacggacgagg
acaa-----------------------------gttcacggaca---cggtgtacagttcaccgataaccaccg---------------------
aacgcagtgataccgtcat-------------------agtggcggctaccgtttcggctgtgttactggtggcaatggtgcttgtggtgg------
tgtactgtcagcaccgctgccaa--
fly_PTP10D ---
atattagcgccaccacgtcctgccacccaagtggtgcccaccga---------------
ggtctatcgcagctcatcgaccatccagattcggtttaggaa-------------------------gaactacttctcggatcaaa--------------
------------acggccaggtgcgcatgtacacgatcatcgtggccgaggatgatgccaagaatgcatccggcctggagatgc------------
--ccagctggctggatgtgcagtcgtacag-----------------------
cgtttggttgccctatc----------------------------aggccatagatccgtactatccattcgagaat-------------------------------
---------------cgatccgtagaggacttcaccatcggtacggaga---------------
actgtgacaaccacaagatcggctactgcaacggaccactgaaatcgggaaccacctatcgggttaaggtgcgggcgttcaccggagcg
gataa------------------------------gttcacggata---ccgcctacagttttcccattcagacag---------------------
atcaagacaacacctc---------------------
actgattgtggccattacggtgccgttaactatcatcttggtgctcctggtgacacttttgttctacaaacgacgtcgc-------------------
----
fly_Ptp52F ---------------------------------------
ctgcccaccattccgagtgatgagcttatcaagcaaatgcgcgccaatgtcgaggagac-----------
atctaatccaacgaaaacggccattgt--tcgccttccagccgacatcatgacatccgcat----
-----------ccggcgacatcaagtggatggcactgatgatctcgcaaaagaactgtgctggagttccacacctcaaatacgat-----------
----------------------------gtcagcagcgattggccaaaggttctatcctatcaagaggccggcgcagatgg---------------
cacaggtgactgcagtctggagtacc-------------------aaaccaccgaggagcgctggcatcccgaacccgttcaacgtcagc------
-----------gaagggatggagaggtgacatccg---atgaggaaattgtttttaccatcggattggaca---------------
agtgttcggaggttcagaaaacgtattgcaatggacctttgttaccagacacggattacaatgttgtggtgagactgttcaccgcatctgg
ttatag---------------------------------------cgatgccgccgtactcaactttaagac-----------------
caaggcggccatcaaggtgaccct-------------------
gatcctggtgagcgtttgcagttgcctgctgctggccttcgtacttggtttgacggtt---
ctctgggtgcgcaagcgattggcctgg
SeaUrchin1 ---------------------------------------
gagccatccccagttccggaagactatccctatgaagccaatacagttttctctga---------------------
gacctctaccacctcgttcgctgtggtgcttccaga--------------------------cgatttattcagccatgaaa------------------------
---acggtgaacttttgacctttgctgtcatcatcacaatagatgataatgatccaactgttagta-------------------------------------
----
gcacagaactgacttatgcggccagagccgagaatgcttaca--tcacagccattgaaatcccatatccatattcaccatcg----------
tttggctctaatcgtagaagacgagcaacgg---atcctccaggtacggtagtcataggtgatggga---------------

cctgtgcaggtagtcaaaatgaatattgcaatggagatttggatgacaacacagaatactactatgcgttcagagcatacaatggcatgg
gtaatgtga---------------------cat-------------ccagtacatttggacctgtgaaaacag---------------------
ttaaagataacacagcggg------------------gattgctgctggggtatccgtctcactgatcatcatcatcgtggctgtagtggtagtt--
gtcgtgttcttaaaaagaagacaaccaaaagaaccttcac----cacgcccaagc--------------
SeaUrchin3 --
gcacctccaactcctcctgcagacagggtggtacgtct--------------tgtttctcatacaggtactaccatgagtgtatcctttgacag--
----------------------tagcatattcagcgatgtct-------------------------
acggtcctgtgactagatatgccctcctggtagcacaaagcagtccagacaccattccttcgacaccaagtgaccctgccatcaac------
----
tggcaagttggctcaggccagccatacc---------------------------aaaccacgctccttacttacgctcctttcacaaacggaa----
---------------------------------acacgagggggttctataccgttggcgctaacaaca-----------
gctgcacgcaggattcagtgggatactgtaacggacctgtcgtccctctcactgattacaggtttgctctacgtgcctacgggtctgatggg
aa----------------------------gttcactgaca---gtgcttggtcaccactctacaactcag---------------------
gaattaacaccacttg---------------------
gtacgctgctgctgtggtttccatcatcatcatcactctcgttctcttactcgtcatgcttctcatctcccgtcaatgc--------------------
----
SeaUrchin4 --
gctccaccgtctccacctcgtggtgcaactccctcgttggtc------tcccgaggcagcactatcaccatcactttttt-------------------
------------------caatctctttgatactgcgt-------------------------
ttggacgaatcgtgcgctttgcaatcattgtgcaggaacgtgttgatggcggtaccgtcgtcatcacagcca-------------------------
-----------------aacgtcagaccacatcttcactcacctgggctgaagccagaaggacacg----------------------
ccctgttccctcctacc---------------------------agaccacgcccgatgactataacccttttgctg----------------------------
---------------atggggcaggtgtaaccaccttcagagttgggtcacagtcct------
gtgatcctgatgatcttactgcctattgcaatggggctctctaccctggtacctcttacagatttgctattagagcgtatggtgtggacggca
a----------------------------gtttgtggata---ctgaattcagctctccatttagaacag---------------------
atcccgacagaggaatatttgt---------------
catcccggcaattgtccttgccatcatggtagtcatcatcctcatcatcttcatggccatgggatttggatgc--------------------------
----
facornWorm --
cctccaccgaaaccaactgcttcacgtattacggcaaatgttcggtt--------------
tgttgagaccaccgcctacacattgcgtattcacctaagtaa------------------------taatttatttgatgacagat---------------
----------ttggaataattgttgcttacttagttatcattgctgaggacggaggtgagtattcaccattgccagaggttc-----------------
---ttccatcatatgatgaagtcattgacagctc----------------------
gccgtggccaccatacc---------------------------aaaccagtcagccattcaatccatttgagacttta---------
tcgattggtagacggcgacgcaatacac------tgcagactgttaattacatcataggcgccaagg--------------
agaattgtgatgtcagtcttgtttactgtaatggggccttaagaccagttacagactacaggtatgtcattcgtggctacaatgaacttggt
aa----------------------------ctacaccgata---cggactggtcacttccacaaaaaacag---------------------------------
----
atttagatccattctggattctgtatggtgtcatagcaggattaatattaattggcatcattatactgatatgtatatgttattgttgctgttgt
agaagaagacgatcatcga----cgcaagactcagacaag----------
acorworm1 ---
ccaccacctcctcccagaaataacatagttccttcatcctctgagtctgacattgaagtttctcaaacaacaatcacagtattattcacaga
------------------------tgattattttaatgattcca-------------------------
atggaccactcttaaatttcactgtcattgtgacagaagatggcactgaggaagttgataagcatctgaatggtgaaataccacgtgtata

ttgtaaagattgtgaaaatgtaccaggtatagaagaaggagaagtgaagacctgggcagaagtacaaaa--------------------------
---cactccagccagatatc----------------------------agattgattatccttatgactatccagattta-------------------
tccagaagaaaacgtgaca---------caacagggattgagattactattggttctgacgata------------
gctgttctacaaaggatgaaggtttctgcaatggtcctttgaaggacggcacatcatatagatatcattttagagcatacacagatgttgg-
-------------------------------atactcagata---ctgtactttcaggtccaattcgaacaa---------------------
gcactaacatgtcatggttgtg----------------
gttacttcttggtgtattgcttggtgtattgatagctgttgctatcattcttgctatcatttattataaacgaagatattatgaagaaactact-
----

ciona --
gcgcccccagtgcctgacacttggcccgaggttatgaacaaaatcgatgatacttca------
acaatggcatctgtgacatcatcgacaataactgttaagaaacc----------------------------atgcatgttctccaatgaga--------
------------------atggaccgattgcaagtctcagtgttataatagcggaggacggaggaaacattgaagccgaaccga-------------
--cgtattggtcgcaggctttccctctgca-----------------------
accttctccaccataca----------------------------aagtcctagtgagtgaaaacccgactgattattgc-------------
aacacaagaaaacgtcgatcaa---------taaatgagaaaggcttcgtcattggtacttcag--------------
actgtccgagtgaactcaacacacattgtaacggcccgcttaaatcaagcacggattacagggtaaaattccgagccgaaactagtaat
ggatt-----------------------------aaagacagata---cggaattttccgaaattatccgaactt-------
ctccgagtttcctcgcaacccatatgacgttgtt------------------
gatcgggattggggctgcaattggattgtttacaattcttgtcgtgatatcagctgta--
tttctacggttagttatgcaatattcgattcaatatg-------gatttcttaacaatgtattc------

Ciona2 --
gcgcctccagtgccggcgttgctaccggaagttattatccaacaagacaaagc-----------------
gattgtgacgtcatcatctgttattgtgaagaaacc----------------------------atgcgttttctccgatgaca---------------------
-----atggaccgcttaagagtctaagtgtaatagttgcacaagagggggcgacgcttgacgctgaaccaa---------------------------
---cttattgggcgaaggcttatagtgagga-----------------------
gccttcaccaccgtata----------------------------aggttattgtaactgaagaacctcaaagttattgc-------------
aactccagaaacaaacgatcaacac------aaaccaacaatggattcgtcatcggaacttccg---------------
attgttctcgtttgtcgacgtcacaatgcaatggaccactgaaatcaaacacagaatatcggtttaaatatcgagccgaggccaacaacg
gcct-----------------------------gatgacagaca---ccgaatattccgaagttttccgaacta-------
atccgagctttatcgaagcgcatatgacgttgtt------------------
agtgtcgattggggcagcacttgggatatttattatacttctgactatatcggtcgca--ttgttacga----------------------------------
----

PTPRF_human --------------------
cacctggtgtccatccgcacagcccccgacctcctgcctcacaagccgctgcctgcctctgcctacatagagga---------------
cggccgcttcgatctctccatgccccatgtgcaagacccc---------------------------tcgcttgtcaggtggttcta-----------------
-------------------------cattgttgtggtgcccattgaccgtgtgggcgggagcatgctgacgccaaggtggagcacaccc-----------
------------------------------gaggaactggagctggacgagcttctagaagccatcgagcaaggcggagaggagcagcggc------
-ggcggcggcggcaggca-------------------------gaacgtctgaagccatatgtggctgctcaactggatg----------------------
----------------------tgctcccggagacctttaccttgggggacaaga-----------------------
agaactaccggggcttctacaaccggcccctgtctccggacttgagctaccagtgctttgtgcttgcctccttgaaggaa----------------
----------------cccatggaccagaagcgctatgcctccagcccctactcggatga------
gatcgtggtccaggtgacaccagcccagcagcaggaggagccggagatgctgtgggtgacgggtcccgtgctggcagtcatcctcatca
tcctcattgtcatcgccatcctc---ttgttcaaaagg------------

PTPRF_mouse --------------------
cacctggtgtccatccgcactgccccggacctcctaccccagaagccactgcctgcctccgcctttatagagga--------------
tggccgcttctccctctccatgcctcaagtgcaggacccc---------------------------tcgctagtcaggtggttcta----------------
-------------------------cattgtggtggtgcccattgaccgtgtgggcgggaacttgctggcaccaagatggaacacacca----------
-------------------------------gaggagttggagctggacgagcttctggaggccatcgagcagggcgaggagaaacagcgga-----
--ggcgccggcgccaagca-------------------------gagcggctgaagccttatgtggcggcccaagtggatg--------------------
-----------------------tgctccctgacaccttcaccctgggggacaaga-----------------------
agagctaccgcggcttctacaaccggcccctgtctccggatctgagttaccagtgcttcgttctcgcctccctcaaggaa----------------
----------------cccatggaccagaagcgctacgcctccagcccctactcggacga------
gattgtagtccaggtgacgccagcacagcagcaggaggagcccgagatgctgtgggtgacaggccctgtcctggcggtcattctcatcat
actcattgtcatcgccatcctc--ctgttcaagagg------------
PTPRF_chiken cag------------------
cacctcgtctccatccgcactgcgcctgacgtcttgcaaagcaaacccattgccaccaacaagtatatccagga--------------
aggaaagttcacgcttacccttcccaaagtgcagaccact---------------------------gtgccagttcggtggtacta---------------
--------------------------cattgtggtcgtgccggcagagca---gagccccagcagcccgacagcgcggtggcggacgcct--------
---------------------------------gatgagatggagctggaccagctgctggaggccataagccagggcagtcagagcaggc--------
--gccagagacgccaagca-------------------------gacagactcaagccctacattgctgcccaagtggacg--------------------
-----------------------tgctgcccgagaccttcaccctgggggacgaga-----------------------
agaactacaagggtttctacaacaagcccctctctcaggacctgagctaccgctgcttcgtgctggcctcgctggaggat--------------
-------------------ggggacacgaagagatacgcagccagcccctactcagatga------
gattgtgatggaattggcttcagcgaagcagcaggatgagccagagatgctgtgggtgatgggacctgtcctggctgtgatattaatcat
catcatcgtcatcgctatactc--ctcttcaaaagg------------
PTPRF_fish cag-----------------
caggtggtgtccatccgcaccgcgcccgacttgctcaaatccaagccggtcatctacagggggcaggaggagga--------------
cggcaagctcaccatctctctgaacagagtttgtatctctgatcgctccagttgcttaaaattaattataacatgttttttataacagaa---
---------------------tcgtatcagtggacagatggcgtgtcacaaccttacaacaaaggtctaaagtcatca-------------------------
---aaagaaggcgcttgcccacgggtgaaagagagcagaaag-------------------
ccttactctgctcctct------------------------ctctagcctgcaacttgtcacagcaggacctgaaaatacatttccttcac-----------
----
tttcccgcaggaattatttaaagttttgcaataataaagttaaaactaatcattttaaaagcctggaaatcctgaaaaagaacttgccata
ttttta-----------------------
caccctcttccagaagacgtttgcggccagcccttactcagaccccataacggtggaggtggtcaacaggatgccgagacagccggagg
agccggagatgttgtgggtgatggggccggtcttggccgtcatcctcatcatcatcattgtcatcgccatcctg------------------------
----------------------ctgtttaag--------------
PTPRF_monosiga
gtgatcttgcgtggtccagggcgcgtggcagagcgccgcacgctttcagcgcggcctgaagcaccgcccgcggtcacgagtg
ccgttctgcctga---------------tggatatagttcacaatcagcctttg---
ctgtcagctgggtcgcaccc--------------tc---
cacgtactcgggtcccatccgtcggctctcgctcgtggtcgagccacaaggcagtagcacccatgccgtcgcctcgttggactgcgcctct
agc--------------------------------------agctgtgatttcgggacgtggtccgaagcccaggctgccag-----------------------
cagcccgatggcttaca---------------------ttgcgtacagccagacctttaccgagtcggagggattgggggatc-----------------
-----------------------tgtctcgaagctcttttgtcattggtgccgagcgag---------
agaaggtcgacgctgacggtgatggttatgtcaatggtcctctcaaagcaggtgccacctacaccttccgtctcctcacctgcacgcaaaa
tggcaacgatgaactgtgccagg--------------------------ccgccagcctcaacccgaccacggcct-

tggcctcgacggcggctgcccccgctgacaatactggcat-------------------
catcgttggagttgtcattgtcatcatcgttctcattgtggccgttcttgtctttgtcatgtggcggcgtcgccagaacaaagc-------------
----cgctgcgcctgatgaagatttctg-----

;

end;]
